# Comparative Study of Toxic Terpenoidal Aldehydes and Lactone Derivatives from the European Polypore *Bondarzewia mesenterica*

**DOI:** 10.1021/acsomega.4c02011

**Published:** 2024-04-11

**Authors:** Winnie Chemutai Sum, Sherif S. Ebada, Harald Kellner, Marc Stadler

**Affiliations:** † Department of Microbial Drugs, Helmholtz Centre for Infection Research GmbH (HZI), Inhoffenstraße 7, 38124 Braunschweig, Germany; ‡ Institute of Microbiology, Technische Universität Braunschweig, Spielmannstraße 7, 38106 Braunschweig, Germany; § Department of Pharmacognosy, Faculty of Pharmacy, Ain Shams University, 11566 Cairo, Egypt; ∥ Department of Bio- and Environmental Sciences, Technische Universität Dresden-International Institute Zittau, Markt 23, 02763 Zittau, Germany

## Abstract

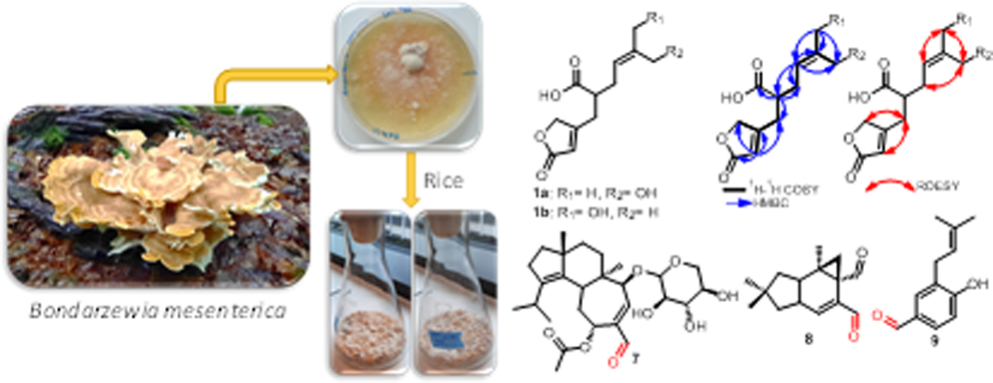

Two unprecedented isomeric secondary metabolites named vibralactones Z_5_ (**1a**) and Z_6_ (**1b**), in addition to eleven known compounds (**2**–**12**), were isolated from solid-state rice culture medium of *Bondarzewia mesenterica* (Bondarzewiaceae). Chemical structures of the isolated compounds were established via spectral analyses. The new lactone derivatives were weakly active against *Staphylococcus aureus* without any significant cytotoxicity, while the molecules containing an aldehyde functionality showed significant antimicrobial and cytotoxic effects. For instance, erinacine P (**7**) and (+)-isovelleral (**8**) and erinacine P (**7**) were cytotoxic against all tested cell lines at IC_50_ values in the ranges of 3.5–14.2 and 2.8–30.2 μM, respectively. In addition, they revealed moderate antimicrobial activity with the lowest minimum inhibitory concentration (MIC) values recorded against *Mucor hiemalis* (8.3 μg/mL), *Pichia anomala*, and *Rhodotorula glutinis* at 16.6 μg/mL.

## 1 Introduction

Basidiomycota continue to be a bountiful source of secondary metabolites that can be used as templates for development of drugs and agrochemicals.^1^ The genus *Bondarzewia* is a remarkable poroid basidiomycete genus whose species are characterized by large imbricate basidiomes. Some of the species are saprotrophs regarded as edible and/or medicinal especially in Asia, whereas others are tree pathogens.^2,3^ The genus was first coined in 1940 by Singer and typified by *Bondarzewia montana*, which is now regarded as a synonym of *Bondarzewia mesenterica*. This species shows apparent host specificity for *Abies* and mainly occurs in old forests.^3^ Currently, there are eleven species accepted in the genus, recorded from Asia, Europe, America, and Oceania, but with no records from Africa thus far.^2^ Phylogenetically, it is closely related to *Heterobasidion*.^2^ Both genera belong to the order Russulales, and an own family Bondarzewiaceae has eventually been created to accommodate them and some other genera.^4^

The genus *Bondarzewia* remains understudied concerning the chemistry of its secondary metabolites, except for a report of the weakly cytotoxic fruiting body constituent montadial and dihydrobenzofuran derivatives from cultures of a Chinese collection of *Bondarzewia berkeleyi*.^5,6^ In the latter study, no biological activity for the isolated metabolites has been reported and no proof was given as to whether the study culture was authentic, even though the specimen was identified by a specialist. In our ongoing attempts to study rare and hitherto untapped members of Basidiomycota, we recently came across a culture of *B. mesenterica* and decided to conduct an in-depth study on its secondary metabolism. The results are the subject of the current paper.

## 2 Results and Discussion

### 2.1 Strain Identification

The fungal isolate IHI 766 was collected by one of the authors (H.K.) at the Bavarian Forest National Park in Bavaria, Germany. Morphologically, it was identified as similar to another *B. mesenterica* isolate (IHI 668) collected at the same location in 2016 and deposited at the International Institute Zittau, Technical University of Dresden (Germany) as DSM 108281. A draft genome of the latter is assembled and available under NCBI Bioproject PRJNA521458.

### 2.2 Structure Elucidation of Secondary Metabolites

**Figure 1:**
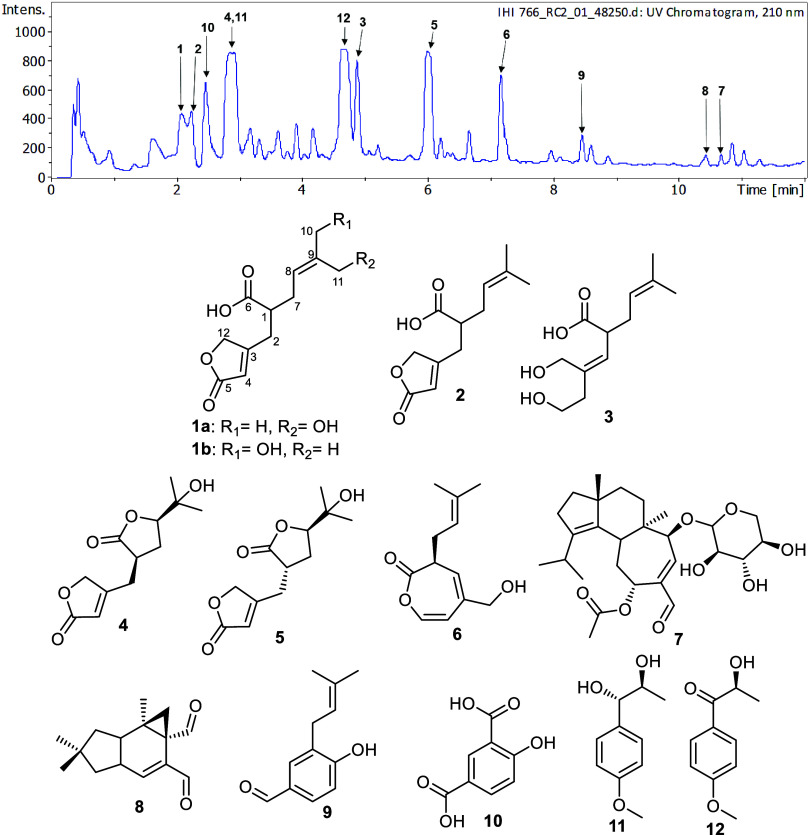
HPLC chromatogram of EtOAc extract derived from*B. mesenterica* rice culture and chemical structures of **1**–**12**.

Screening of the ethyl acetate (EtOAc) extract derived from *B. mesenterica* using high-performance liquid chromatography equipped with diode-array detection-mass spectrometry (HPLC-DAD-MS) in conjunction with secondary metabolite database searches on the dictionary of natural products (https://dnp.chemnetbase.com/chemical/ChemicalSearch.xhtml?dswid=-621) showed that new and known compounds were at hand (Figure 1). Chromatographic fractionation and purification of the total extract yielded two previously undescribed metabolites **1a** and **1b** (Figure 1), given trivial names vibralactones Z_5_ and Z_6_. In addition, chemical exploration of the extract afforded eleven known derivatives (Figure 1) that were identified based on high-resolution electrospray ionization-mass spectrometry (HR-ESI-MS), one-/two-dimensional (1/2D) NMR spectroscopic analyses, and the comparisons with the reported literature to be recognized as vibralactones J (**2**),^7^ Z_1_ (**4**) and Z_2_ (**5**),^8^ sterepinic acid A (**3**),^9^ 1,5-secovibralactone (**6**),^10^ erinacine P (**7**),^11^ (+)-isovelleral (**8**),^12−14^ 4-hydroxy-3(3-methylbut-2-enyl)benzaldehyde (**9**),^15^ 4-hydroxyisophthalic acid (**10**),^16^ (4-methoxyphenyl)-1,2-propanediol (**11**),^17,18^ and 2-hydroxy-(4′-methoxy)-propiophenone (**12**).^17^ All of the isolated compounds were assessed for their cytotoxic and antimicrobial activities against a panel of cell lines and microbes, respectively.

Compound **1** was obtained as a yellow oil that revealed a single peak during both analytical and preparative chromatographic separations. The molecular formula of **1** was established as C_12_H_16_O_5_, indicating five degrees of unsaturation based on HR-ESI-MS that revealed a protonated molecule at *m*/*z* 241.1065 [M + H]^+^ (calculated 241.1071) and a sodium adduct at *m*/*z* 263.0885 [M + Na]^+^ (calculated 263.0890). The ^1^H NMR spectrum of **1** (Table 1, Figure S3) revealed the presence of two sets of comparable proton resonances in a ratio of 5:6 according to their integration indices indicating its possible existence as an inseparable mixture of two isomeric derivatives (**1a** and **1b**). The same notion was also obvious in the ^13^C NMR spectrum (Table 1, Figure S4) that revealed twined peaks of comparable resonances and can be recognized into four unprotonated sp^2^ carbon pairs namely two carbonyl carbon pairs at δ_C_ 177.69/177.73 (C-6) and 176.6 (C-5), two olefinic carbon pairs at δ_C_ 171.97/172.03 (C-3) and 139.05/139.16 (C-9). In addition, the ^13^C NMR spectral data of **1a** and **1b** (Table 1) revealed the presence of two tertiary sp^2^ carbon atoms each corresponding to an isomer at δ_C_ 124.4 and 121.9 that were directly correlated via the heteronuclear single quantum coherence (HSQC) spectrum (Figure S7) to two olefinic protons at δ_H_ 5.29 (*td*, *J* = 7.7, 1.5 Hz, 1H) and at δ_H_ 5.43 (*td*, *J* = 7.3, 1.4 Hz, 1H). The ^13^C NMR spectral data of **1** unveiled the presence of one pair of electromagnetically equivalent methine sp^2^ carbon atoms at δ_C_ 116.5 (C-4) directly correlated via the HSQC spectrum to two electromagnetically equivalent olefinic protons at δ_H_ 5.91 (*q*, *J* = 1.7 Hz, 2H), one pair of methine sp^3^ carbon atoms at δ_C_ 45.2/45.0 (C-1), four pairs of methylene sp^3^ carbons at δ_C_ 31.00/30.89 (C-2), δ_C_ 31.27/31.34 (C-7), an electromagnetically equivalent pair at δ_C_ 75.0 (C-12), and an oxygenated methylene pair at δ_C_ 61.2 (C-11 in **1a**)/68.5 (C-10 in **1b**). The interpreted spectral data accounted for four degrees of unsaturation and thus suggesting that **1a** and **1b** comprisemonocyclic structures. A literature search based on the obtained results revealed that **1a** and **1b** are closely related to vibralactone J (**2**)^7^ with a clear difference of the absence of two allylic methyl groups and the emergence of two hydroxymethylene moieties instead. Apart from that difference, the ^1^H and ^13^C NMR data of both **1a**/**1b** and **2** came in close accordance.

**Table 1:** ^1^H and ^13^C NMR Spectral Data of Vibralactones Z_5_ (**1a**) and Z_6_ (**1b**)

	**1a**		**1b**	
pos.	δ_C_,^a^,^c^ type	δ_H_^b^ (multi, *J* [Hz])	δ_C_,^a^,^c^ type	δ_H_^b^ (multi, *J* [Hz])
1	45.2, CH	2.76 m (overlapped)	45.0, CH	2.78 m (overlapped)
2	31.00, CH_2_	α 2.64 ddt (12.7, 7.5, 2.0)	30.89, CH_2_	α 2.66 ddt (12.7, 7.5, 2.0)
		β 2.76 m (overlapped)		β 2.78 m (overlapped)
3	171.97, CO		172.03, CO	
4	116.5, CH	5.91 q (1.7)	116.5, CH	5.91 q (1.7)
5	176.6, CO		176.6, CO	
6	177.69, CO		177.73, CO	
7	31.27, CH_2_	α 2.39 m (overlapped)	31.34, CH_2_	α 2.39 m (overlapped)
		β 2.47 dt (14.3, 6.7)		β 2.47 dt (14.3, 6.7)
8	124.4, CH	5.29 td (7.7, 1.5)	121.9, CH	5.43 tq (7.3, 1.4)
9	139.05, C		139.16, C	
10	21.7, CH_3_	1.79 d (1.5)	68.5, CH_2_	3.94 d (1.4)
11	61.2, CH_2_	α 4.05 d (12.2)	13.90, CH_3_	1.66 d (1.4)
		β 4.09 d (12.2)		
12	75.0, CH_2_	α 4.84 dd (17.8, 2.0)	75.0, CH_2_	α 4.84 dd (17.8, 2.0)
		β 4.88 dd (17.8, 1.7)		β 4.88 dd (17.8, 1.7)

a Measured in methanol-*d*_4_ at 125 MHz.

b At 500 MHz.

c Assignment confirmed by HMBC and HSQC spectra.

**Figure 2:**
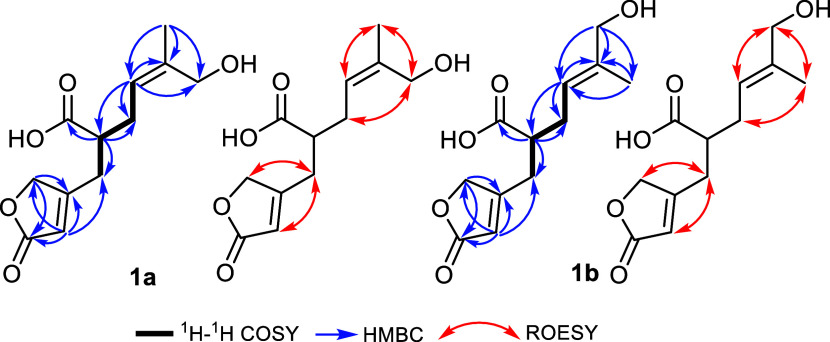
Key ^1^H–^1^H COSY, HMBC, and ROESY correlations of **1a** and **1b**.

Further confirmation to the depicted structures of **1a** and **1b** was obtained by a ^1^H–^1^H COrrelated SpectroscopY (COSY) spectrum (Figure 2) that revealed the presence of two comparable spin systems extending over H-8/H_2_-7/H-1/H_2_-2, whereas the heteronuclear multiple bond correlation (HMBC) spectrum (Figure 2) confirmed the presence of one lactone ring in each isomer by revealing key correlations from diastereotopic methylene protons at δ_H_ 4.84/4.88 (*dd*, *J* = 17.9, 1.7 Hz, H_2_-12) and an olefinic methine proton at δ_H_ 5.91 (*q*, *J* = 1.7 Hz, H-4) to one carbonyl carbon at C-5 (δ_C_ 176.6) and a deshielded quaternary carbon pair at δ_C_ 171.97/172.03 (C-3), thus confirming the presence of comparable α,β-unsaturated lactone rings in **1a** and **1b**, respectively. To distinguish the depicted orientation of terminal hydroxymethylene moieties in **1a** and **1b**, the rotating-frame Overhauser spectroscopy (ROESY) spectrum (Figure 2) was recorded and revealed key ROE correlations from diastereotopic methylene protons at δ_H_ 4.05/4.09 (*d*, *J* = 12.2 Hz, H_2_-11) to diastereotopic methylene protons at δ_H_ 2.39/2.47 (H_2_-7) in **1a**, whereas the hydroxymethylene protons in **1b** at δ_H_ 3.94 (*d*, *J* = 1.4 Hz, H_2_-10) revealed key ROE correlation to an olefinic proton at δ_H_ 5.43 (*tq*, *J* = 7.3, 1.4 Hz, H-8), confirming the presence of hydroxymethylene groups at C-11 and C-10 in **1a** and **1b**, respectively. Both compounds **1a** and **1b** revealed a single secondary carboxylic acid chiral carbon (C-1) resembling vibralactone J (**2**) and sterepinic acid A (**3**). Being isolated as an inseparable mixture hindered determining their absolute configuration by the phenylglycine methyl ester (PGME) method that previously established (*S*) configuration for sterepinic acid A.^9^ By comparing the measured optical rotation values of **1a**/**1b** ([α]_D_^20^ + 12°, *c* 0.1 in methanol) and **3** ([α]_D_^20^ + 37°, *c* 0.1 in methanol) to that reported for sterepinic acid A ([α]_D_^22^ + 58°, *c* 0.34 in acetonitrile)^9^ in addition to be of a common biosynthetic origin, then at least one of the compounds **1a**/**1b** could presumably feature a similar (*S*) configuration at C-1. Based on the aforementioned results, compound **1** was unambiguously confirmed to be a mixture of two isomeric vibralactone derivatives and hence were trivially named as vibralactones Z_5_ (**1a**) and Z_6_ (**1b**).

To the best of our knowledge, (+)-isovelleral (**8**) was unprecedented in fungal cultures and this encouraged us to investigate whether it is a genuine product or evolved from an enzymatic conversion of stearyl-velutinal as previously reported in the literature.^13^ In order to define its source, two different mycelial portions of equal age were separately extracted by ethyl acetate. One portion was directly extracted without any mechanical treatment while the other was crushed and kept for 30 min before extraction. Both extracts were individually analyzed using HPLC-DAD-MS to screen for the molecular weight of 530 g/mol ascribed to stearyl-velutinal. The obtained results revealed the presence of (+)-isovelleral (**8**) in comparable abundances in both extracts as that obtained in the main extract of this study. Intriguingly, no traces of stearyl-velutinal could be detected in all of the analyses supporting its probable production during an advanced growth phase of the fungus in its natural habitat as a defense mechanism against competing predators or parasites, rather than being induced by lab cultivation. However, upon trying the same extraction scheme on dry fruiting bodies of *B. mesenterica*, we could detect neither stearyl-velutinal nor isovelleral.

### 2.3 Biological Activities of Compounds **1**–**12**

The isolated compounds demonstrated various biological effects in the antimicrobial and cytotoxicity assays based on our standardized methods.^19^ The obtained results (Table 2) unveiled that the new vibralactones Z_5_/Z_6_ (**1**) together with Z_2_ (**5**) and sterepinic acid A (**3**) had weak antibacterial effects against *Staphylococcus aureus* at minimum inhibitory concentration (MIC) values of 66.6 μg/mL. The compounds were non-cytotoxic to cell lines L929 and KB3.1; hence, they were not further tested.

**Table 2:** Cytotoxicity (IC_50_) and Antimicrobial Activity (MIC) of **1**–**12^a^**

	IC_50_ (μM)											positive control
test cell line	**1**	**2**	**3**	**4**	**5**	**6**	**7**	**8**	**9**	**11**	**12**	epothilone B (nM)
mouse fibroblast (L929)	n.a.	n.a.	n.a.	n.a.	n.a.	n.a.	13.0	10.3	n.a.	n.a.	n.a.	0.65
human endocervical adenocarcinoma (KB3.1)	n.a.	n.a.	n.a.	n.a.	n.a.	n.a.	5.1	10.3	n.a.	n.a.	n.a.	0.17
human prostate carcinoma (PC-3)	n.d.	n.d.	n.d.	n.d.	n.d.	n.d.	4.9	3.9	n.d.	n.d.	n.d.	0.09
human breast adenocarcinoma (MCF-7)	n.d.	n.d.	n.d.	n.d.	n.d.	n.d.	3.3	2.8	n.d.	n.d.	n.d.	0.07
human ovarian cancer (SKOV-3)	n.d.	n.d.	n.d.	n.d.	n.d.	n.d.	7.7	5.2	n.d.	n.d.	n.d.	0.09
human epidermoid carcinoma (A431)	n.d.	n.d.	n.d.	n.d.	n.d.	n.d.	3.5	3.4	n.d.	n.d.	n.d.	0.06
human lung carcinoma (A549)	n.d.	n.d.	n.d.	n.d.	n.d.	n.d.	14.2	30.2	n.d.	n.d.	n.d.	0.05
test microorganism	MIC (μg/mL)											positive control (μg/mL)
*Staphylococcus aureus*	66.6	n.i.	66.6	n.i.	66.6	n.i.	66.6	33.3	n.i.	n.i.	n.i.	0.42^G^
*Escherichia coli*	n.i.	n.i.	n.i.	n.i.	n.i.	n.i.	n.i.	n.i.	n.i.	n.i.	n.i.	0.83^G^
*Bacillus subtilis*	n.i.	n.i.	n.i.	n.i.	n.i.	n.i.	33.3	33.3	n.i.	n.i.	n.i.	16.6°
*Pseudomonas aeruginosa*	n.i.	n.i.	n.i.	n.i.	n.i.	n.i.	n.i.	n.i.	n.i.	n.i.	n.i.	0.42^G^
*Pichia anomala*	n.i.	n.i.	n.i.	n.i.	n.i.	n.i.	n.i.	16.6	33.3	n.i.	n.i.	8.30^N^
*Candida albicans*	n.i.	n.i.	n.i.	n.i.	n.i.	n.i.	n.i.	33.3	n.i.	n.i.	n.i.	8.30^N^
*Acinetobacter baumanii*	n.i.	n.i.	n.i.	n.i.	n.i.	n.i.	n.i.	n.i.	n.i.	n.i.	n.i.	1.04^C^
*Chromobacterium violaceum*	n.i.	n.i.	n.i.	n.i.	n.i.	n.i.	n.i.	n.i.	n.i.	n.i.	n.i.	1.67^G^
*Schizosaccharomyces pombe*	n.i.	n.i.	n.i.	n.i.	n.i.	n.i.	n.i.	33.3	n.i.	n.i.	n.i.	8.30^N^
*Mucor hiemalis*	n.i.	n.i.	n.i.	n.i.	n.i.	66.6	66.6	8.3	66.6	n.i.	n.i.	8.30^N^
*Rhodotorula glutinis*	n.i.	n.i.	n.i.	n.i.	n.i.	n.i.	16.6	16.6	66.6	n.i.	n.i.	4.20^N^
*Mycobacterium smegmatis*	n.i.	n.i.	n.i.	n.i.	n.i.	n.i.	n.i.	33.3	n.i.	n.i.	n.i.	1.70^K^

a n.a.: no activity; n.i.: no inhibition up to 67 μg/mL; n.d.: not determined. G: gentamycin; O: oxytetracycline; N: nystatin; C: ciprofloxacin; K: kanamycin.

Notably, erinacine P (**7**) and (+)-isovelleral (**8**), comprising an aldehyde functionality in their structures, were the most active compounds against all tested cell lines with IC_50_ values within the ranges of 3.3–14.2 and 2.8–30.2 μM, respectively. Unlike the other compounds, **7** and **8** also exhibited antimicrobial effects to some extent, albeit weakly (Table 2) with (+)-isovelleral (**8**) being the most active against *M. hiemalis* at MIC value of 8.3 μg/mL. According to a previous report, (+)-isovelleral (**8**) revealed mutagenicity alongside its antimicrobial effects.^13^ In addition, the unsaturated monoaldehyde compound **9** (Table 2) had weak inhibitory activities against *P. anomala*, *M. hiemalis*, and *Rhodotorula glutinis* (MIC values of 33.3–66.6 μg/mL), with no apparent cytotoxic effects.

## 3 Conclusions

To this end, we have studied secondary metabolites, mostly consisting of lactone moieties from *B. mesenterica*. It is noteworthy that similar derivatives have been widely reported from the basidiomycete *Boreostereum vibrans* (formerly *Stereum vibrans*),^20,21^ a fungus considered as a “talented strain” due to its ability to produce a vast array of natural products.^8^ Nevertheless, isolation of similar compounds from different fungi belonging to the same class in the phylum Basidiomycota is a commonly encountered scenario. Notably, the aldehyde functionality has been shown to affect the antimicrobial and cytotoxic effects similar to the isolated cyathane-xyloside (**7**)^19,22^ and sesquiterpenoid (**8**).^23^ The presence of two aldehyde groups in (+)-isovelleral (**8**) has been demonstrated to be responsible for its mutagenic, antimicrobial, cytotoxic, and antifeedant properties.^13,23^

## 4 Materials and Methods

### 4.1 General Experimental Procedures

HPLC-DAD-MS analyses were performed on an amaZon speed ETD ion trap mass spectrometer (Bruker Daltonics, Bremen, Germany) on positive and negative ionization modes. The HPLC system consisted of a Dionex UltiMate 3000 UHPLC (Thermo Fisher Scientific Inc., Waltham, MA) equipped with a C_18_ Acquity UPLC BEH column (Waters, Milford). The solvent phase consisted of solvent A (deionized H_2_O + 0.1% formic acid) and solvent B (MeCN + 0.1% formic acid). The separation gradient was as follows: 5% solvent B for 0.5 min, 5–100% solvent B over 20 min, and holding the gradient isocratically at 100% solvent B for 10 min. The flow rate employed was 0.6 mL/min, and UV–Vis detections were recorded at 190–600 nm. HR-(+)ESI-MS data were recorded on a maXis ESI-TOF (Time-Of-Flight) mass spectrometer (Bruker Daltonics, Bremen, Germany) connected to an Agilent 1260 series HPLC-UV system (Agilent Technologies, Santa Clara, CA) equipped with a C_18_ Acquity UPLC BEH column (Waters). The solvent system consisted of solvent A (deionized H_2_O + 0.1% formic acid) and solvent B (MeCN + 0.1% formic acid). The separation gradient was as follows: 5% solvent B for 0.5 min, 5–100% solvent B over a period of 19.5 min, and eventually holding solvent B at 100% for 5 min. The flow rate employed was 0.6 mL/min at 40 °C and the UV–Vis detection was recorded at 200–600 nm. Molecular formulas of the detected compounds were calculated using the Smart Formula algorithm of the Compass Data Analysis software (Bruker, version 6.1).

An Avance III 500 (^1^H 500 MHz, ^13^C 125 MHz, Bruker Daltonics, Bremen, Germany) spectrometer was used to record 1D and 2D NMR spectra of compounds dissolved in methanol-*d*_4_. Chemical shifts were recorded in parts per million (ppm), and coupling constants were calculated in Hertz (Hz). UV–Vis spectra were obtained using a Shimadzu UV–Vis 2450 spectrophotometer (Kyoto, Japan) at a concentration of 0.02 mg/mL in methanol Uvasol (Merck, Darmstadt, Germany). Optical rotations were recorded on an Anton Paar MCP-150 Polarimeter (Graz, Austria) with a sodium D line at 589 nm and 100 mm path length at a concentration of 1.0 mg/mL in methanol.

The solvents and chemicals (analytical and HPLC grades) used were sourced from AppliChem GmbH (Darmstadt, Germany), Avantor Performance Materials (Deventer, Netherlands), Carl Roth GmbH & Co. KG (Karlsruhe, Germany), and Merck (Darmstadt, Germany). The Purelab flex water purification system (Veolia Water Technologies, Celle, Germany) was used to prepare deionized water.

### 4.2 Collection of the Fungal Specimen and Preparation of Cultures

Basidiocarps of *B. mesenterica* were collected at Bavarian Forest National Park (location: Mittelsteighütte) in Bavaria, Germany, in August 2021, on the base (putatively emerging from the buried root) of an old-growth *Abies alba* by one of the authors (H.K.). The mycelial cultures were established from the fresh basidiomes and maintained on YMG (4 g of yeast extract, 10 g of malt extract, 4 g of glucose, and 20 g of agar in 1 L of deionized water) medium.

### 4.3 Fermentation of Cultures and Extraction of Metabolites

YMG agar plates were prepared and used to inoculate fresh pieces of basidiomes to obtain axenic cultures as previously described.^19^ Subsequently, YMG agar plates with fully grown mycelia of *B. mesenterica* were used to inoculate 6 × 500 mL Erlenmeyer culture flasks containing sterile rice medium. After 55 days of static incubation at 24 °C in the dark, secondary metabolites were extracted as initially reported.^24^ In brief, the fermented cultures were initially soaked overnight using 3 × 500 mL of acetone per flask and sonicated at 40 °C. The filterate was evaporated until an aqueous phase was attained and then partitioned twice against EtOAc (1:1). The aqueous phase was discarded, and the EtOAc phase was dried under vacuum to yield the total extract (1.5 g).

### 4.4 Isolation of Compounds and Their Physicochemical Properties

The EtOAc extract (1.5 g) was fractionated on a preparative HPLC (PLC 2020; Gilson, Middleton, WI) system. The eluents consisted of deionized H_2_O + 0.1% formic acid (solvent A) and MeCN + 0.1% formic acid (solvent B) using a C_18_ VP-Nucleodur column 100-5 (250 × 40 mm, 7 μm: Macherey-Nagel, Düren, Germany). The flow rate was maintained at 40 mL/min and the UV detections made at 190, 210, and 280 nm wavelengths.

The implemented gradient to elute the compounds was as follows: an initial isocratic condition at 5% solvent B for 10 min, followed by an increase from 5 to 50% of solvent B within 60 min, thereafter from 50 to 100% solvent B in 20 min, and a final holding of the gradient at 100% solvent B for 20 min. This procedure yielded **2** (10.2 mg, *t*_R_ = 23 min), **5** (17.1 mg, *t*_R_ = 25 min), **1a**/**1b** (5.0 mg, *t*_R_ = 27 min), **4** (5.5 mg, *t*_R_ = 30 min), **3** (7.3 mg, *t*_R_ = 31 min), **6** (10.9 mg, *t*_R_ = 39 min), **11** (2.9 mg, *t*_R_ = 42 min), **7** (3.9 mg, *t*_R_ = 44 min), **12** (11.5 mg, *t*_R_ = 45 min), **10** (0.7 mg, *t*_R_ = 52 min), **8** (2.2 mg, *t*_R_ = 87 min), and **9** (2.9 mg, *t*_R_ = 96 min).

Vibralactones Z_5_ (**1a**) and Z_6_ (**1b**): yellow oil; [α]_D_^20^ + 12° (*c* 0.1, methanol); UV–Vis (MeOH): λ_max_ (log ε) = 204.0 (1.37) nm; NMR data (^1^H NMR: 500 MHz, ^13^C NMR: 125 MHz, methanol-*d*_4_) see Table 1; HR-(+)ESI-MS: *m*/*z* 223.0958 [M – H_2_O + H]^+^ (calcd. 223.0965 for C_12_H_15_O_4_^+^), 241.1065 [M + H]^+^ (calcd. 241.1071 for C_12_H_17_O_5_^+^), 263.0885 [M + Na]^+^ (calcd. 263.0890 for C_12_H_16_NaO_5_^+^), 481.2066 [2M + H]^+^ (calcd. 481.2068 for C_24_H_33_O_10_^+^), 503.1884 [2M + Na]^+^ (calcd. 503.1888 for C_24_H_32_NaO_10_^+^); *t*_R_ = 3.15 min (LR-ESI-MS).

### 4.5 Antimicrobial Assay

The antimicrobial effects of the isolated compounds were determined using our established protocols.^19^ Generally, an array of clinically relevant microorganisms consisting of bacteria, namely, *S. aureus* (DSM 346), *A. baumanii* (DSM 30008), *B. subtilis* (DSM 10), *E. coli* (DSM 1116), *P. aeruginosa* (PA14), *C. violaceum* (DSM 30191), and *M. smegmatis* (ATCC 700084), and fungi including *M. hiemalis* (DSM 2656), *C. albicans* (DSM 1665), *R. glutinis* (DSM 10134), *S. pombe* (DSM 70572), and *P. anomala* (DSM 6766), were used. Gentamycin, oxytetracycline, ciprofloxacin, or kanamycin was used as positive control against tested bacterial strains, while nystatin was used as a positive control against fungal pathogens as indicated in Table 2. The minimum inhibitory concentrations (MIC) were recorded after an overnight incubation as the lowest concentration under which no microbial growth was visualized. The microorganisms were obtained from the German Collection of Microorganisms and Cell Cultures (DSMZ, Braunschweig, Germany).

### 4.6 Cytotoxicity (MTT) Assay

MTT (3-(4,5-dimethylthiayol-2-yl)-2,5-diphenyltetrazolium bromide)-based assay was used to determine *in vitro* cytotoxicity as previously reported.^24^ The tested mammalian cell lines included mouse fibroblasts (L929), endocervical adenocarcinoma (KB3.1), human lung carcinoma (A549), breast adenocarcinoma (MCF-7), prostate carcinoma (PC-3), and epidermoid carcinoma cells (A431). Epothilone B was used as a positive control.

## Supporting Information Available

All HPLC-ESI-MS, HRESIMS, 1D (^1^H/^13^C), and 2D (^1^H–^1^H COSY, HMBC, HSQC, and ROESY) spectra of **1**–**12** (PDF)
