# Supplementary material for: Comparative Study of Toxic Terpenoidal Aldehydes and Lactone Derivatives from the European Polypore Bondarzewia mesenterica
Source: ACS Omega. 2024 Apr 11;9(16):18668–73. doi: 10.1021/acsomega.4c02011 (PMC11044139; doi:10.1021/acsomega.4c02011)
Supplement: Supplementary file 1 — ao4c02011_si_001.pdf [file ao4c02011_si_001.pdf]

SUPPORTING INFORMATION FOR

Comparative study of toxic terpenoidal aldehydes and lactone derivatives from the European polypore *Bondarzewia mesenterica*

Winnie Chemutai Sum,[†, ‡] Sherif S. Ebada,*[†,⊥] Harald Kellner,[§] and Marc Stadler*[†, ‡]

[†] Department of Microbial Drugs, Helmholtz Centre for Infection Research GmbH (HZI), Inhoffenstraße 7, 38124 Braunschweig, Germany

[‡] Institute of Microbiology, Technische Universität Braunschweig, Spielmannstraße 7, 38106 Braunschweig, Germany

[⊥] Department of Pharmacognosy, Faculty of Pharmacy, Ain Shams University, 11566 Cairo, Egypt

[§] Department of Bio- and Environmental Sciences, Technische Universität Dresden-International Institute Zittau, Markt 23, 02763 Zittau, Germany

* Correspondence: sherif.elsayed@helmholtz-hzi.de; sherif_elsayed@pharma.asu.edu.eg (S.S.E.); Marc.Stadler@helmholtz-hzi.de (M.S.); Tel.: +49-531-6181-4240; Fax +49-531-6181-9499

TABLE OF CONTENTS

#	Contents	Page
**1**	Figure S1. LRESIMS of **1**.	**S4**
**2**	Figure S2. HRESIMS of **1**.	**S5**
**3**	Figure S3. ^{1}H NMR spectrum of **1** in methanol-d_4 at 500 MHz.	**S6**
**4**	Figure S4. ^{13}C NMR spectrum of **1** in methanol-d_4 at 125 MHz.	**S7**
**5**	Figure S5. ^{1}H-^{1}H COSY spectrum of **1** in methanol-d_4 at 500 MHz.	**S8**
**6**	Figure S6. HMBC spectrum of **1** in methanol-d_4 at 500 MHz.	**S9**
**7**	Figure S7. HSQC spectrum of **1** in methanol-d_4 at 500 MHz.	**S10**
**8**	Figure S8. ROESY spectrum of **1** in methanol-d_4 at 500 MHz.	**S11**
**9**	Figure S9. LRESIMS of **2**.	**S12**
**10**	Figure S10. HRESIMS of **2**.	**S13**
**11**	Figure S11. ^{1}H NMR spectrum of **2** in methanol-d_4 at 500 MHz.	**S14**
**12**	Figure S12. ^{13}C NMR spectrum of **2** in methanol-d_4 at 125 MHz.	**S15**
**13**	Figure S13. ^{1}H-^{1}H COSY spectrum of **2** in methanol-d_4 at 500 MHz.	**S16**
**14**	Figure S14. HMBC spectrum of **2** in methanol-d_4 at 500 MHz.	**S17**
**15**	Figure S15. HSQC spectrum of **2** in methanol-d_4 at 500 MHz.	**S18**
**16**	Figure S16. ROESY spectrum of **2** in methanol-d_4 at 500 MHz.	**S19**
**17**	Figure S17. LRESIMS of **3**.	**S20**
**18**	Figure S18. HRESIMS of **3**.	**S21**
**19**	Figure S19. ^{1}H NMR spectrum of **3** in methanol-d_4 at 500 MHz.	**S22**
**20**	Figure S20. ^{13}C NMR spectrum of **3** in methanol-d_4 at 125 MHz.	**S23**
**21**	Figure S21. ^{1}H-^{1}H COSY spectrum of **3** in methanol-d_4 at 500 MHz.	**S24**
**22**	Figure S22. HMBC spectrum of **3** in methanol-d_4 at 500 MHz.	**S25**
**23**	Figure S23. HSQC spectrum of **3** in methanol-d_4 at 500 MHz.	**S26**
**24**	Figure S24. ROESY spectrum of **3** in methanol-d_4 at 500 MHz.	**S27**
**25**	Figure S25. LRESIMS of **4**.	**S28**
**26**	Figure S26. HRESIMS of **4**.	**S29**
**27**	Figure S27. ^{1}H NMR spectrum of **4** in methanol-d_4 at 500 MHz.	**S30**
**28**	Figure S28. ^{13}C NMR spectrum of **4** in methanol-d_4 at 125 MHz.	**S31**
**29**	Figure S29. ^{1}H-^{1}H COSY spectrum of **4** in methanol-d_4 at 500 MHz.	**S32**
**30**	Figure S30. HMBC spectrum of **4** in methanol-d_4 at 500 MHz.	**S33**
**31**	Figure S31. HSQC spectrum of **4** in methanol-d_4 at 500 MHz.	**S34**
**32**	Figure S32. ROESY spectrum of **4** in methanol-d_4 at 500 MHz.	**S35**
**33**	Figure S33. LRESIMS of **5**.	**S36**
**34**	Figure S34. HRESIMS of **5**.	**S37**
**35**	Figure S35. ^{1}H NMR spectrum of **5** in methanol-d_4 at 500 MHz.	**S38**
**36**	Figure S36. ^{13}C NMR spectrum of **5** in methanol-d_4 at 125 MHz.	**S39**
**37**	Figure S37. ^{1}H-^{1}H COSY spectrum of **5** in methanol-d_4 at 500 MHz.	**S40**
**38**	Figure S38. HMBC spectrum of **5** in methanol-d_4 at 500 MHz.	**S41**
**39**	Figure S39. HSQC spectrum of **5** in methanol-d_4 at 500 MHz.	**S42**
**40**	Figure S40. ROESY spectrum of **5** in methanol-d_4 at 500 MHz.	**S43**
**41**	Figure S41. LRESIMS of **6**.	**S44**
**42**	Figure S42. HRESIMS of **6**.	**S45**
**43**	Figure S43. ^{1}H NMR spectrum of **6** in methanol-d_4 at 500 MHz.	**S46**
**44**	Figure S44. ^{13}C NMR spectrum of **6** in methanol-d_4 at 125 MHz.	**S47**
**45**	Figure S45. ^{1}H-^{1}H COSY spectrum of **6** in methanol-d_4 at 500 MHz.	**S48**
**46**	Figure S46. HMBC spectrum of **6** in methanol-d_4 at 500 MHz.	**S49**
**47**	Figure S47. HSQC spectrum of **6** in methanol-d_4 at 500 MHz.	**S50**
**48**	Figure S48. ROESY spectrum of **6** in methanol-d_4 at 500 MHz.	**S51**

**49**	Figure S49. LRESIMS of **7**.	**S52**
**50**	Figure S50. HRESIMS of **7**.	**S53**
**51**	Figure S51. ^{1}H NMR spectrum of **7** in methanol-d_4 at 500 MHz.	**S54**
**52**	Figure S52. ^{13}C NMR spectrum of **7** in methanol-d_4 at 125 MHz.	**S55**
**53**	Figure S53. ^{1}H-^{1}H COSY spectrum of **7** in methanol-d_4 at 500 MHz.	**S56**
**54**	Figure S54. HMBC spectrum of **7** in methanol-d_4 at 500 MHz.	**S57**
**55**	Figure S55. HSQC spectrum of **7** in methanol-d_4 at 500 MHz.	**S58**
**56**	Figure S56. ROESY spectrum of **7** in methanol-d_4 at 500 MHz.	**S59**
**57**	Figure S57. LRESIMS of **8**.	**S60**
**58**	Figure S58. HRESIMS of **8**.	**S61**
**59**	Figure S59. ^{1}H NMR spectrum of **8** in methanol-d_4 at 500 MHz.	**S62**
**60**	Figure S60. ^{13}C NMR spectrum of **8** in methanol-d_4 at 125 MHz.	**S63**
**61**	Figure S61. ^{1}H-^{1}H COSY spectrum of **8** in methanol-d_4 at 500 MHz.	**S64**
**62**	Figure S62. HMBC spectrum of **8** in methanol-d_4 at 500 MHz.	**S65**
**63**	Figure S63. HSQC spectrum of **8** in methanol-d_4 at 500 MHz.	**S66**
**64**	Figure S64. ROESY spectrum of **8** in methanol-d_4 at 500 MHz.	**S67**
**65**	Figure S65. LRESIMS of **9**.	**S68**
**66**	Figure S66. HRESIMS of **9**.	**S69**
**67**	Figure S67. ^{1}H NMR spectrum of **9** in methanol-d_4 at 500 MHz.	**S70**
**68**	Figure S68. ^{13}C NMR spectrum of **9** in methanol-d_4 at 125 MHz.	**S71**
**69**	Figure S69. ^{1}H-^{1}H COSY spectrum of **9** in methanol-d_4 at 500 MHz.	**S72**
**70**	Figure S70. HMBC spectrum of **9** in methanol-d_4 at 500 MHz.	**S73**
**71**	Figure S71. HSQC spectrum of **9** in methanol-d_4 at 500 MHz.	**S74**
**72**	Figure S72. ROESY spectrum of **9** in methanol-d_4 at 500 MHz.	**S75**
**73**	Figure S73. LRESIMS of **10**.	**S76**
**74**	Figure S74. HRESIMS of **10**.	**S77**
**75**	Figure S75. ^{1}H NMR spectrum of **10** in methanol-d_4 at 500 MHz.	**S78**
**76**	Figure S76. ^{13}C NMR spectrum of **10** in methanol-d_4 at 125 MHz.	**S79**
**77**	Figure S77. ^{1}H-^{1}H COSY spectrum of **10** in methanol-d_4 at 500 MHz.	**S80**
**78**	Figure S78. HMBC spectrum of **10** in methanol-d_4 at 500 MHz.	**S81**
**79**	Figure S79. HSQC spectrum of **10** in methanol-d_4 at 500 MHz.	**S82**
**80**	Figure S80. LRESIMS of **11**.	**S83**
**81**	Figure S81. HRESIMS of **11**.	**S84**
**82**	Figure S82. ^{1}H NMR spectrum of **11** in methanol-d_4 at 500 MHz.	**S85**
**83**	Figure S83. ^{13}C NMR spectrum of **11** in methanol-d_4 at 125 MHz.	**S86**
**84**	Figure S84. ^{1}H-^{1}H COSY spectrum of **11** in methanol-d_4 at 500 MHz.	**S87**
**85**	Figure S85. HMBC spectrum of **11** in methanol-d_4 at 500 MHz.	**S88**
**86**	Figure S86. HSQC spectrum of **11** in methanol-d_4 at 500 MHz.	**S89**
**87**	Figure S87. ROESY spectrum of **11** in methanol-d_4 at 500 MHz.	**S90**
**88**	Figure S88. LRESIMS of **12**.	**S91**
**89**	Figure S89. HRESIMS of **12**.	**S92**
**90**	Figure S90. ^{1}H NMR spectrum of **12** in methanol-d_4 at 500 MHz.	**S93**
**91**	Figure S91. ^{13}C NMR spectrum of **12** in methanol-d_4 at 125 MHz.	**S94**
**92**	Figure S92. ^{1}H-^{1}H COSY spectrum of **12** in methanol-d_4 at 500 MHz.	**S95**
**93**	Figure S93. HMBC spectrum of **12** in methanol-d_4 at 500 MHz.	**S96**
**94**	Figure S94. HSQC spectrum of **12** in methanol-d_4 at 500 MHz.	**S97**
**95**	Figure S95. ROESY spectrum of **12** in methanol-d_4 at 500 MHz.	**S98**

Generic Display Report

Analysis Info

Analysis Name	S:\PEOPLE\sel22_Sherif Elsayed\Bondarzewia\AmaZon\IHI 766R2F4_GD4_01_50336.d	Acquisition Date	02.09.2023 04:52:29
Method	50336.m	Operator	tti
Sample Name	IHI 766R2F4	Instrument	amaZon speed
Comment			

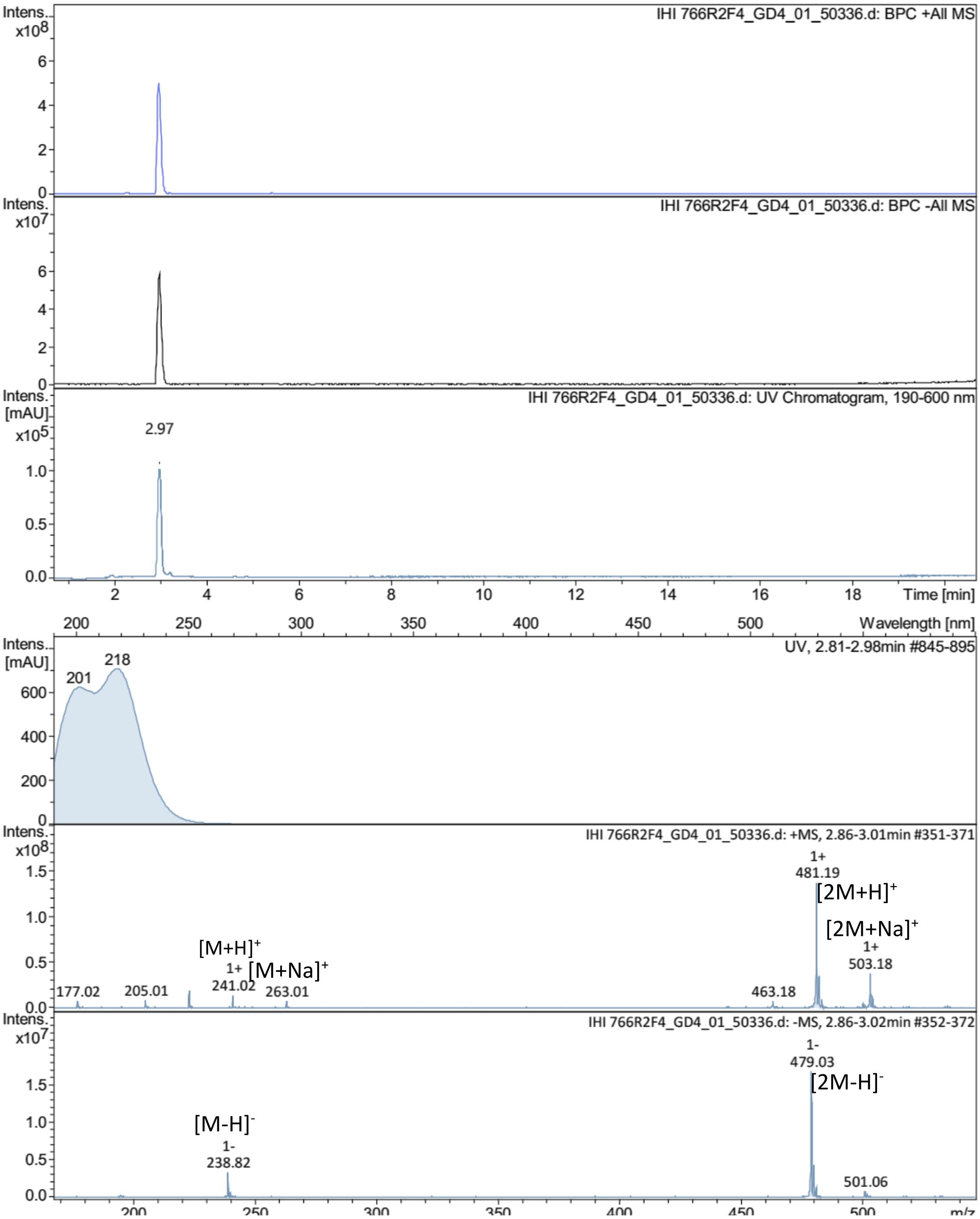

Figure S1. LRESIMS of **1**.

Generic Display Report

Analysis Info

Analysis Name	F:\Volume D\HZI Projects\Winnie\8-Bondarzewia mesenterica\Bondarzewia\MaXis\IHI 766 R2F4_22_01_13118.d	Acquisition Date	05.09.2023 17:47:42
Method	pos_screening_100_2500_line.m	Operator	ate06
Sample Name	IHI 766 R2F4	Instrument	maXis
Comment	Screening01<br>Waters Acquity UPLC BEH C_18 1,7um 2.1x50mm		

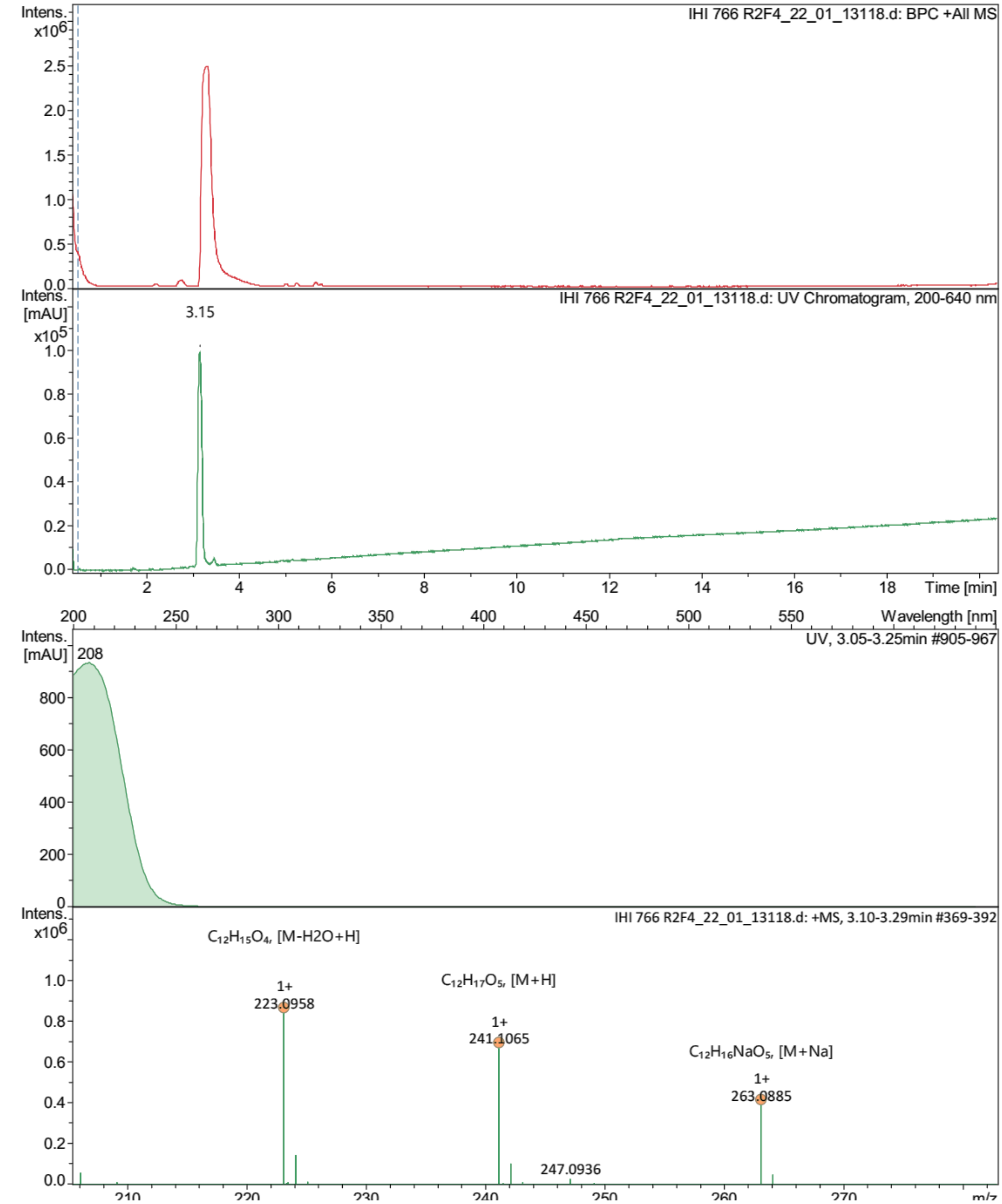

Figure S2. HRESIMS of **1**.

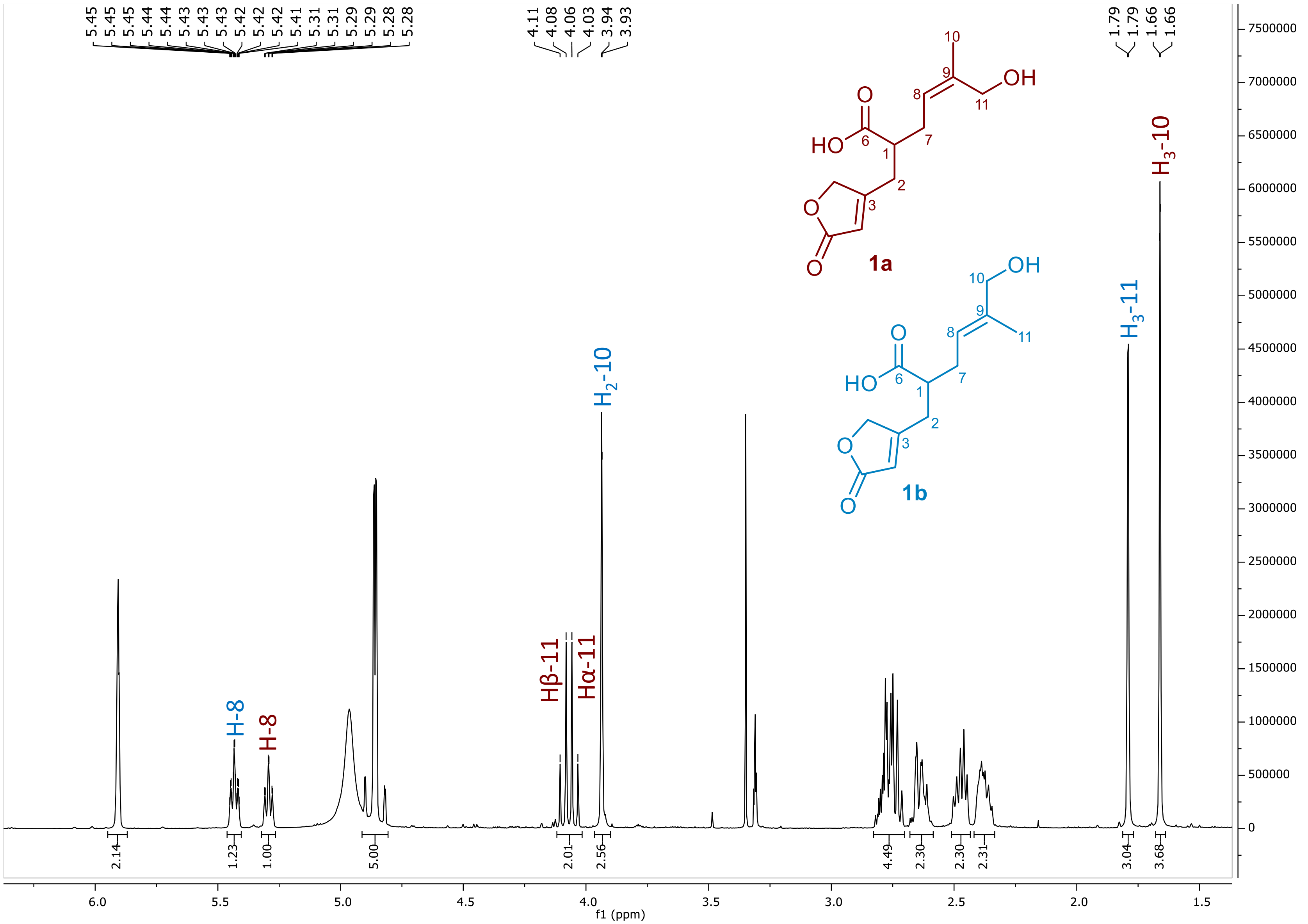

Figure S3. ^{1}H NMR spectrum of **1** in methanol-*d*$_4$ at 500 MHz.

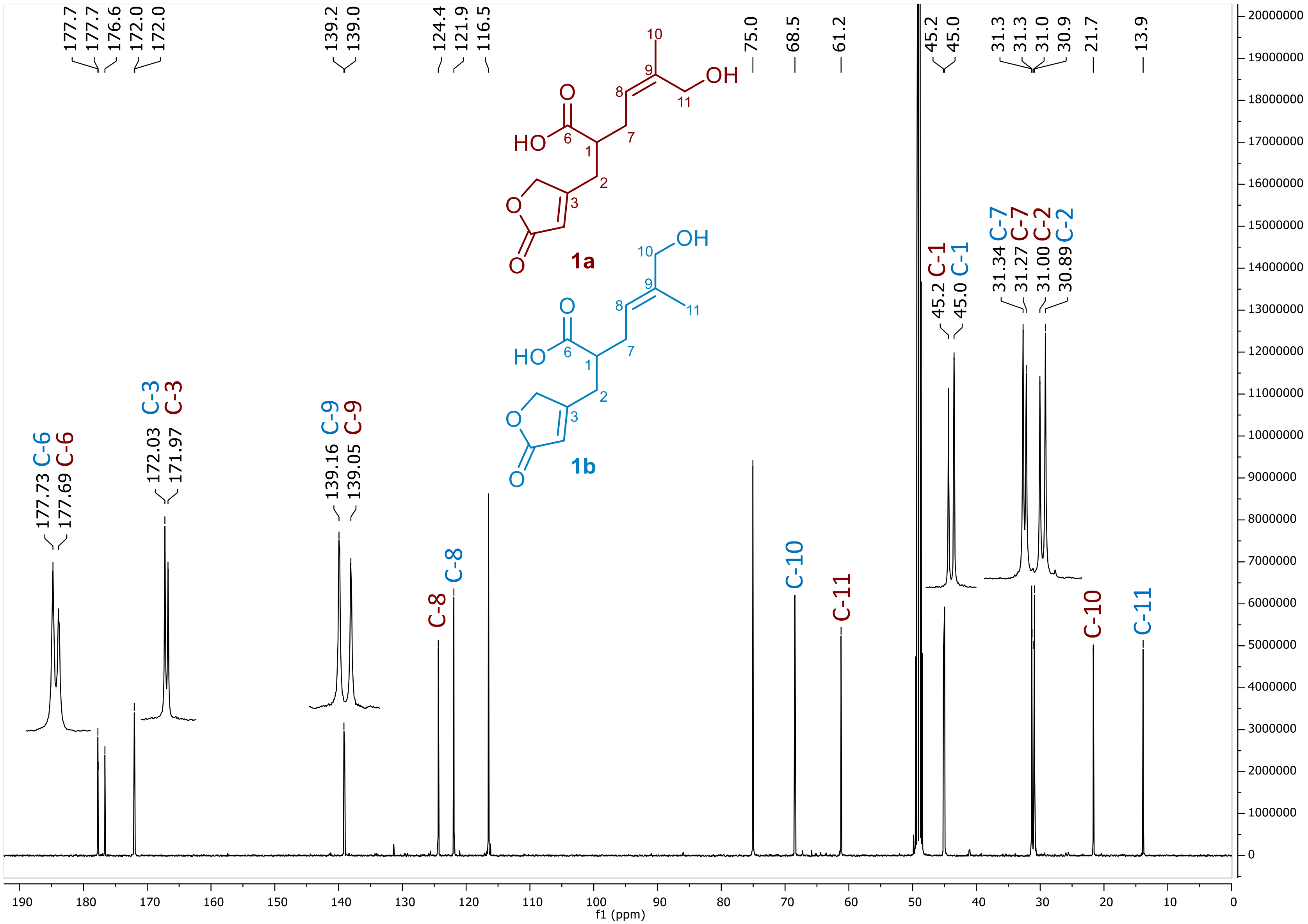

Figure S4. ^{13}C NMR spectrum of **1** in methanol-d_4 at 125 MHz.

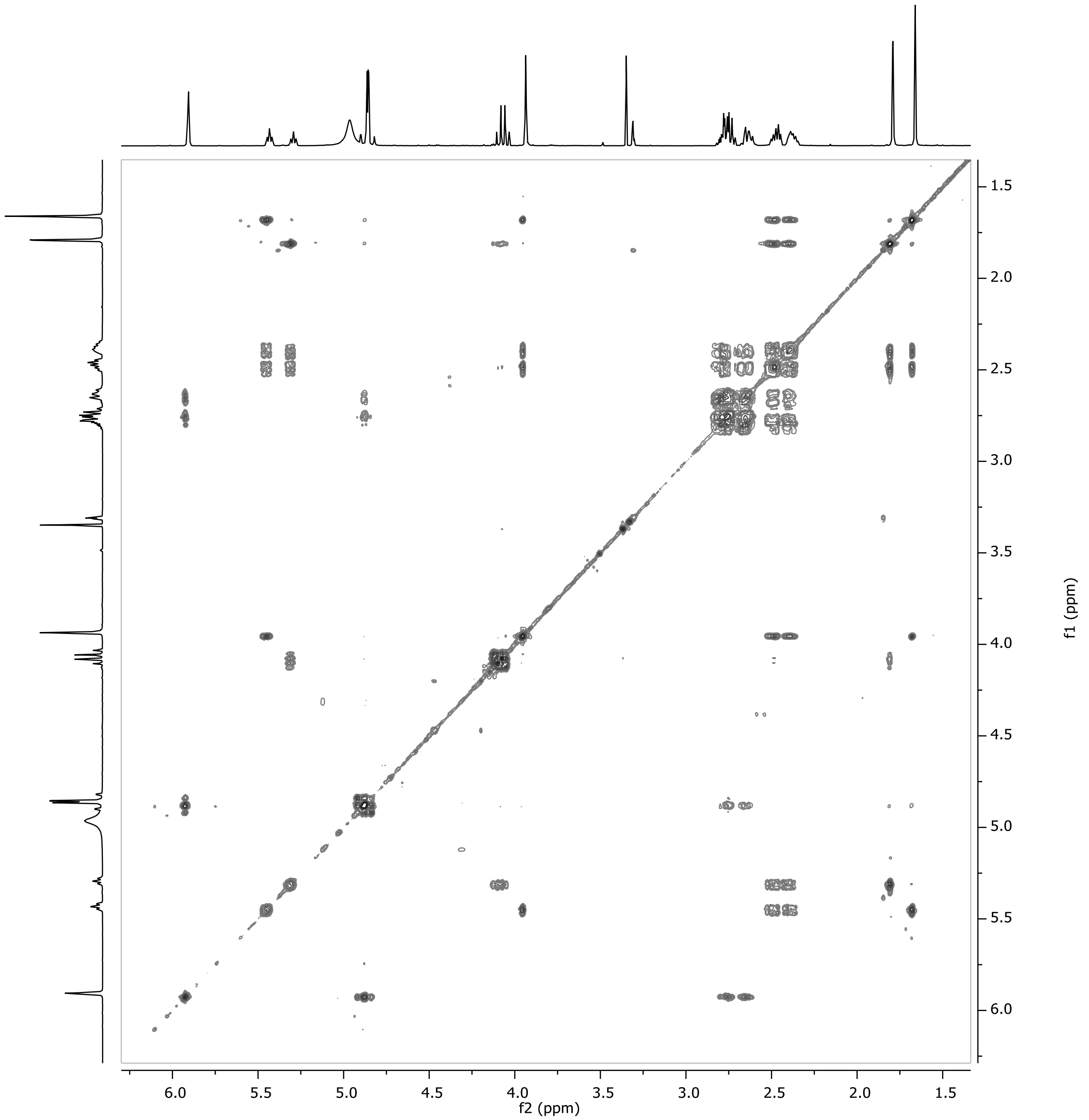

Figure S5. ^{1}H-^{1}H COSY spectrum of **1** in methanol-d_4 at 500 MHz.

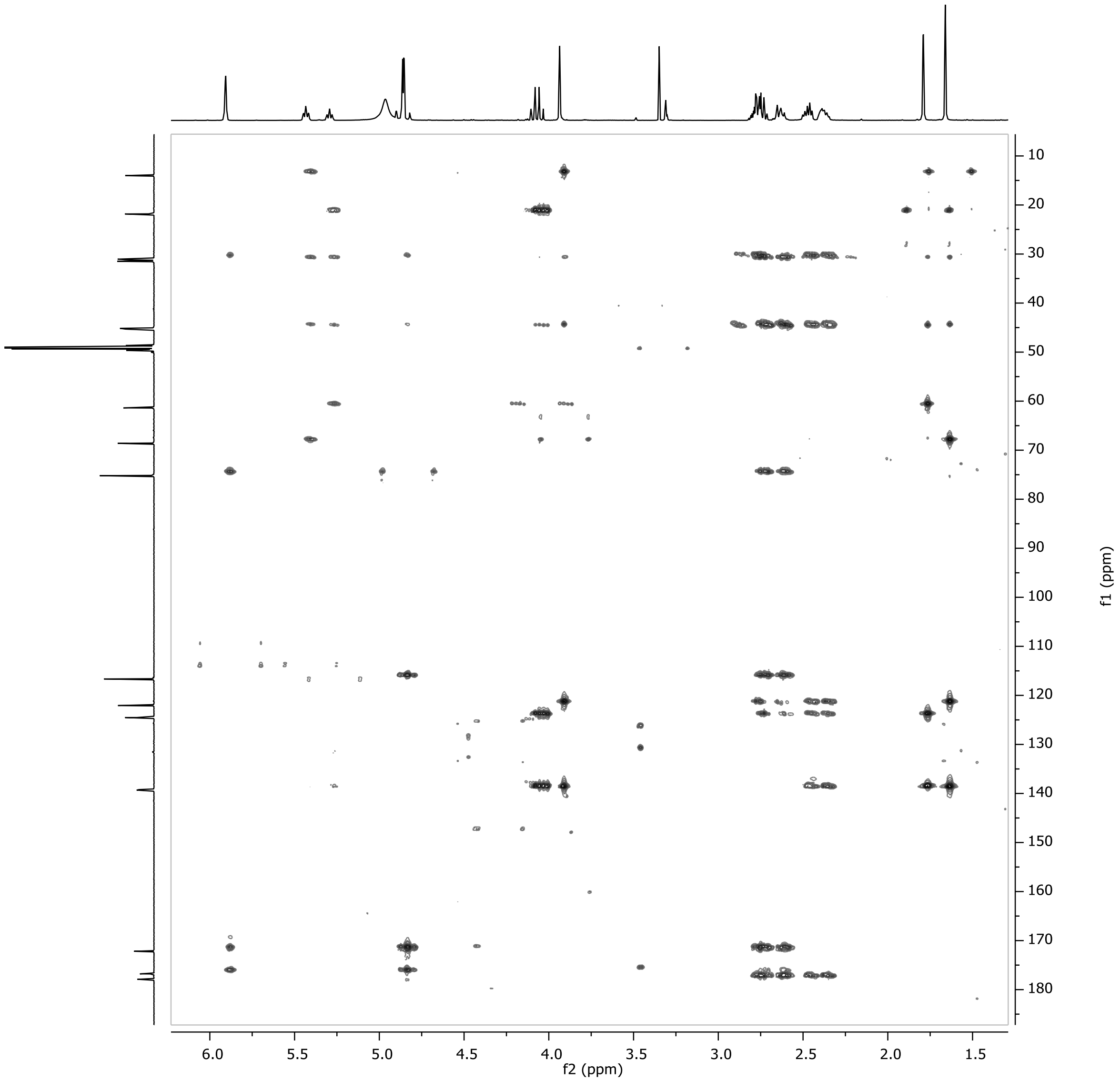

Figure S6. HMBC spectrum of **1** in methanol-d_4 at 500 MHz.

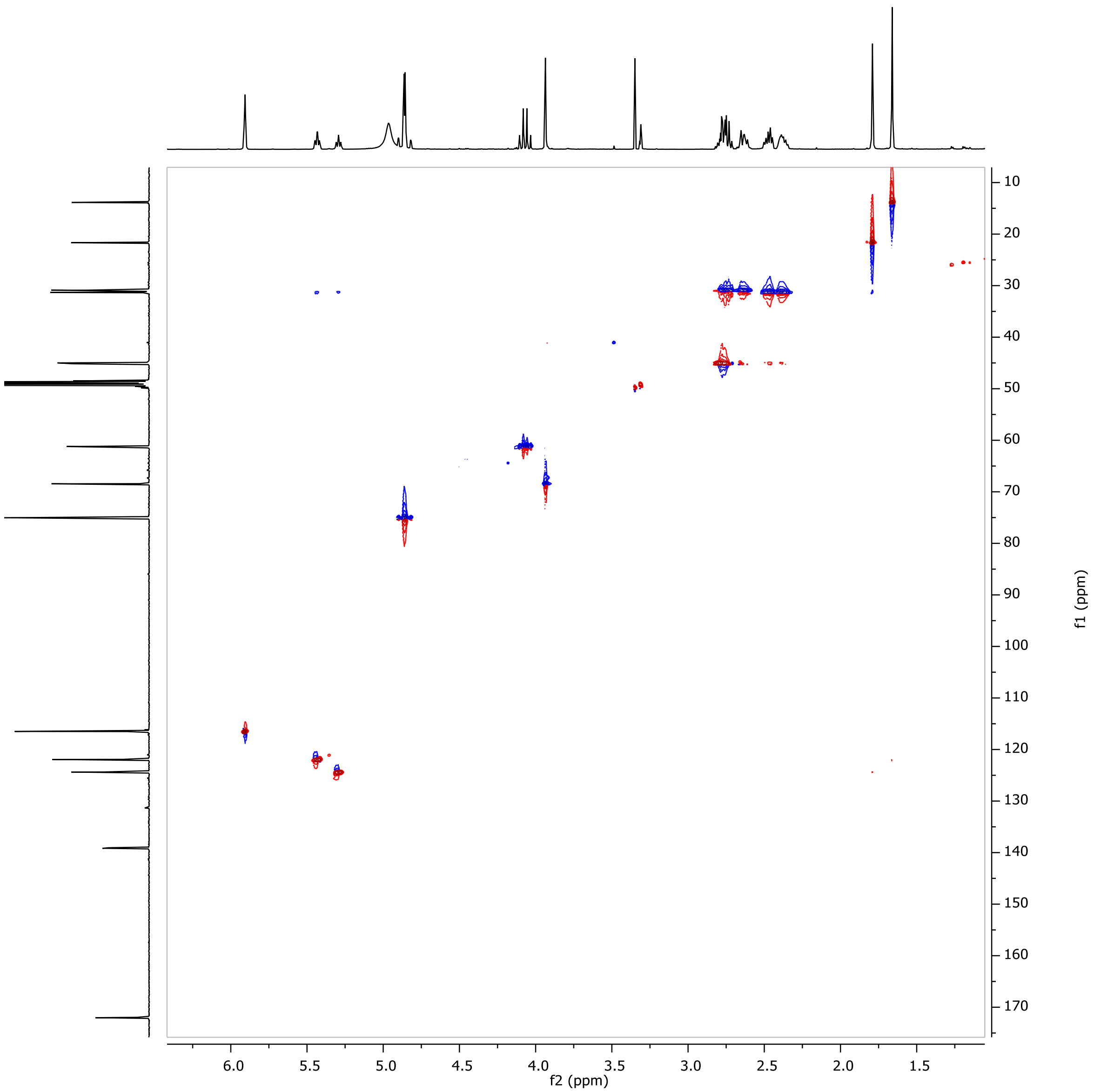

Figure S7. HSQC spectrum of **1** in methanol-d_4 at 500 MHz.

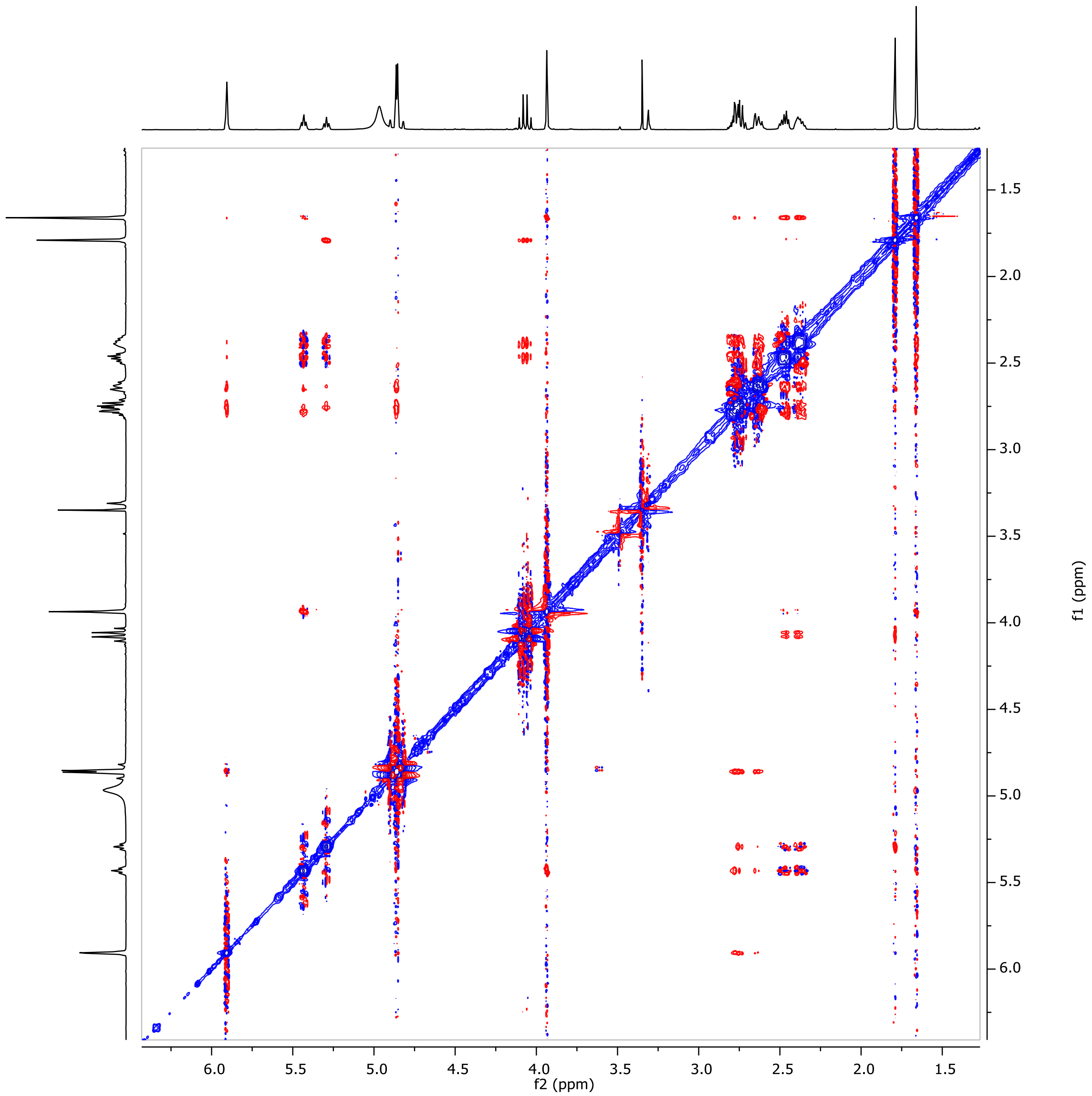

Figure S8. ROESY spectrum of **1** in methanol-d_4 at 500 MHz.

Generic Display Report

Analysis Info

Analysis Name	S:\PEOPLE\sel22_Sherif Elsayed\Bondarzewia\AmaZon\IHI 766R2F12_GE4_01_50346.d	Acquisition Date	02.09.2023 10:54:59
Method	50346.m	Operator	tti
Sample Name	IHI 766R2F12	Instrument	amaZon speed
Comment			

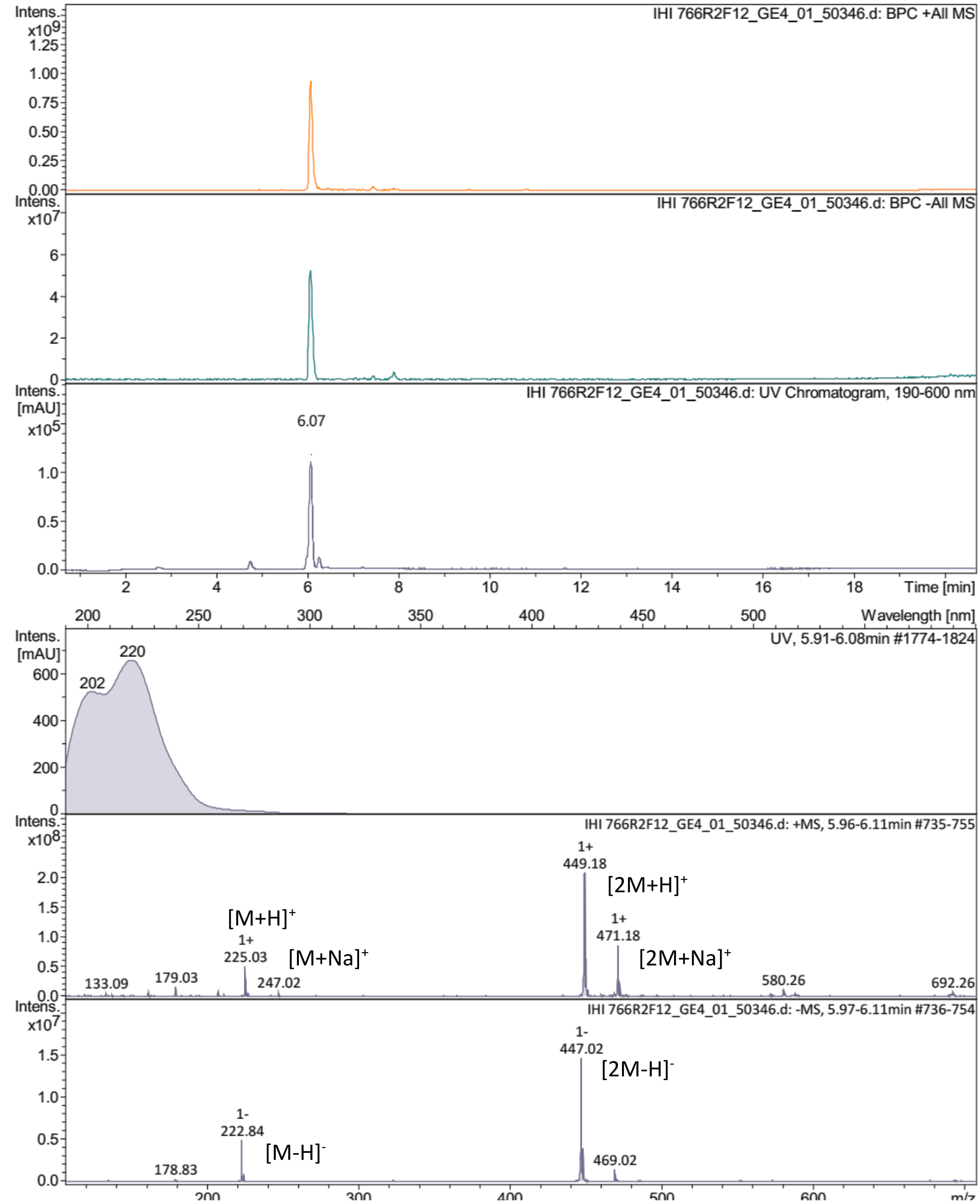

Bruker Compass DataAnalysis 4.4 printed: 11.10.2023 17:51:56 by: sel22 Page 1 of 1

Figure S9. LRESIMS of **2**.

Generic Display Report

Analysis Info

Analysis Name	F:\Volume D\HZI Projects\Winnie\8-Bondarzewia mesenterica\Bondarzewia\MaXis\IHI 766 R2F12_26_01_13122.d	Acquisition Date	05.09.2023 19:51:33
Method	pos_sauce_000_screening_ms_100_2500_line.m	Operator	ate06
Sample Name	IHI 766 R2F12	Instrument	maXis
Comment	Screening01<br>Waters Acquity UPLC BEH C_18 1,7um 2.1x50mm		

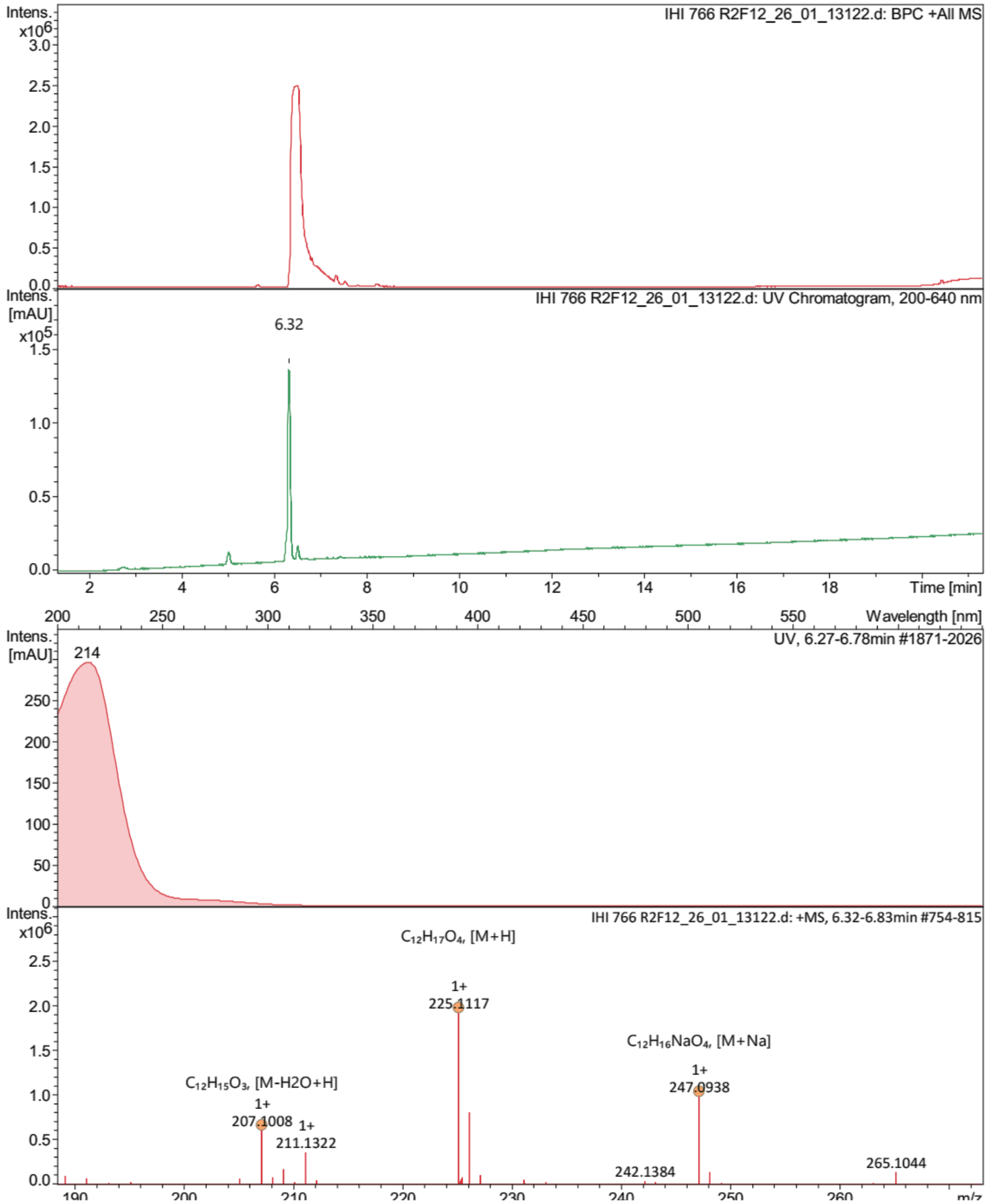

Bruker Compass DataAnalysis 4.4 printed: 11.10.2023 16:17:49 by: sel22 Page 1 of 1

Figure S10. HRESIMS of **2**.

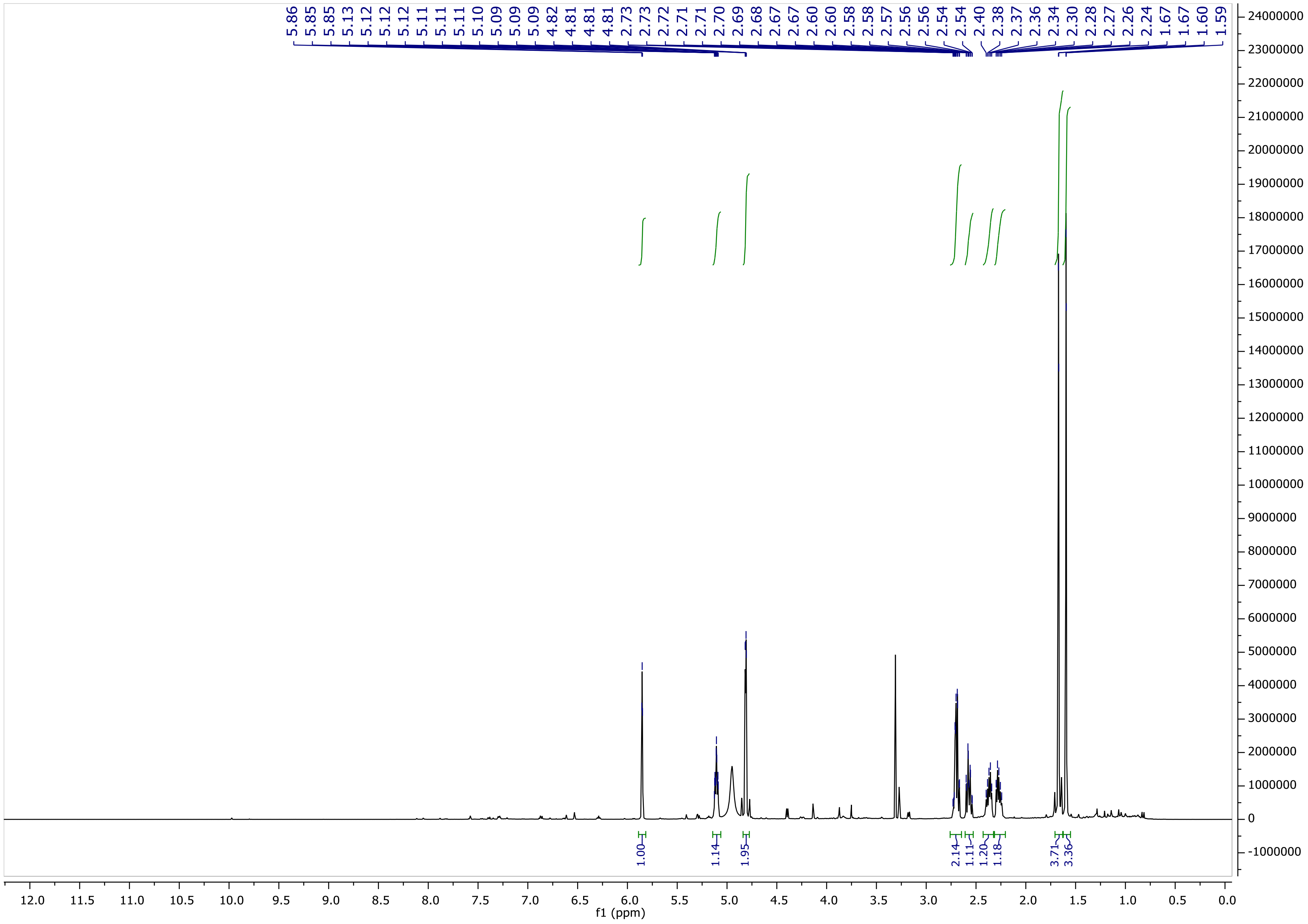

Figure S11. ^{1}H NMR spectrum of **2** in methanol-d_4 at 500 MHz.

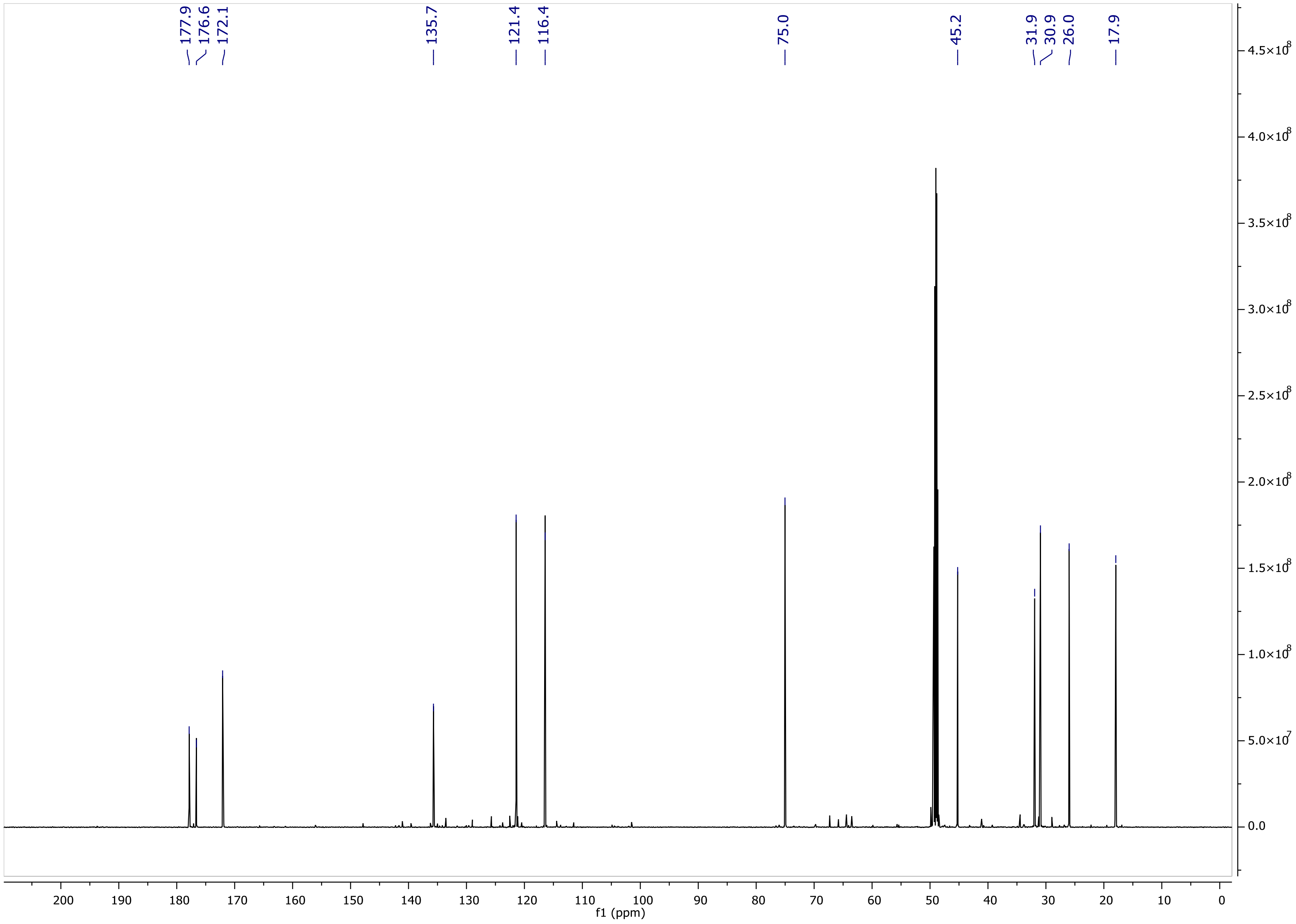

Figure S12. ^{13}C NMR spectrum of **2** in methanol-d_4 at 125 MHz.

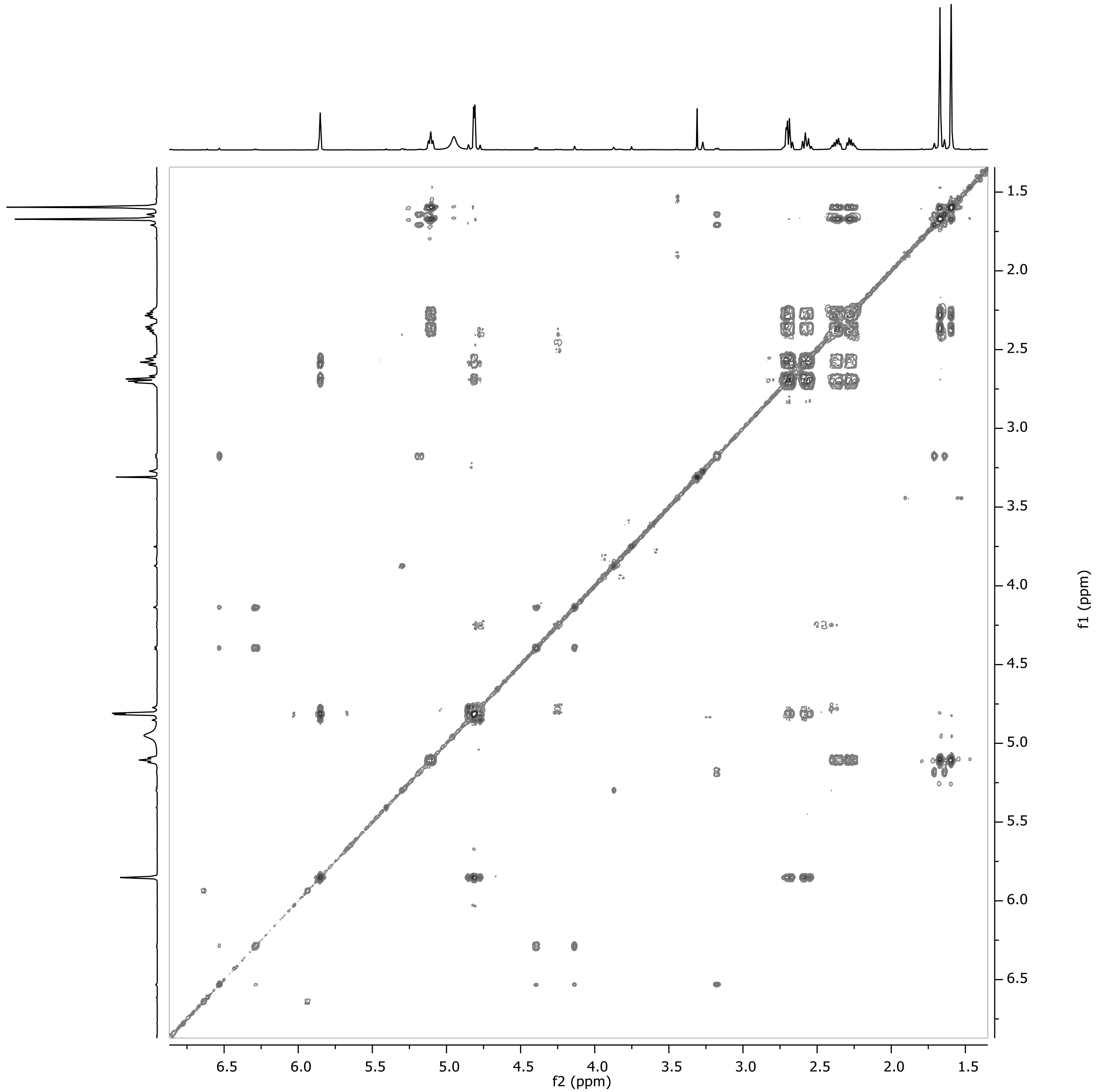

Figure S13. ^{1}H-^{1}H COSY spectrum of **2** in methanol-d_4 at 500 MHz.

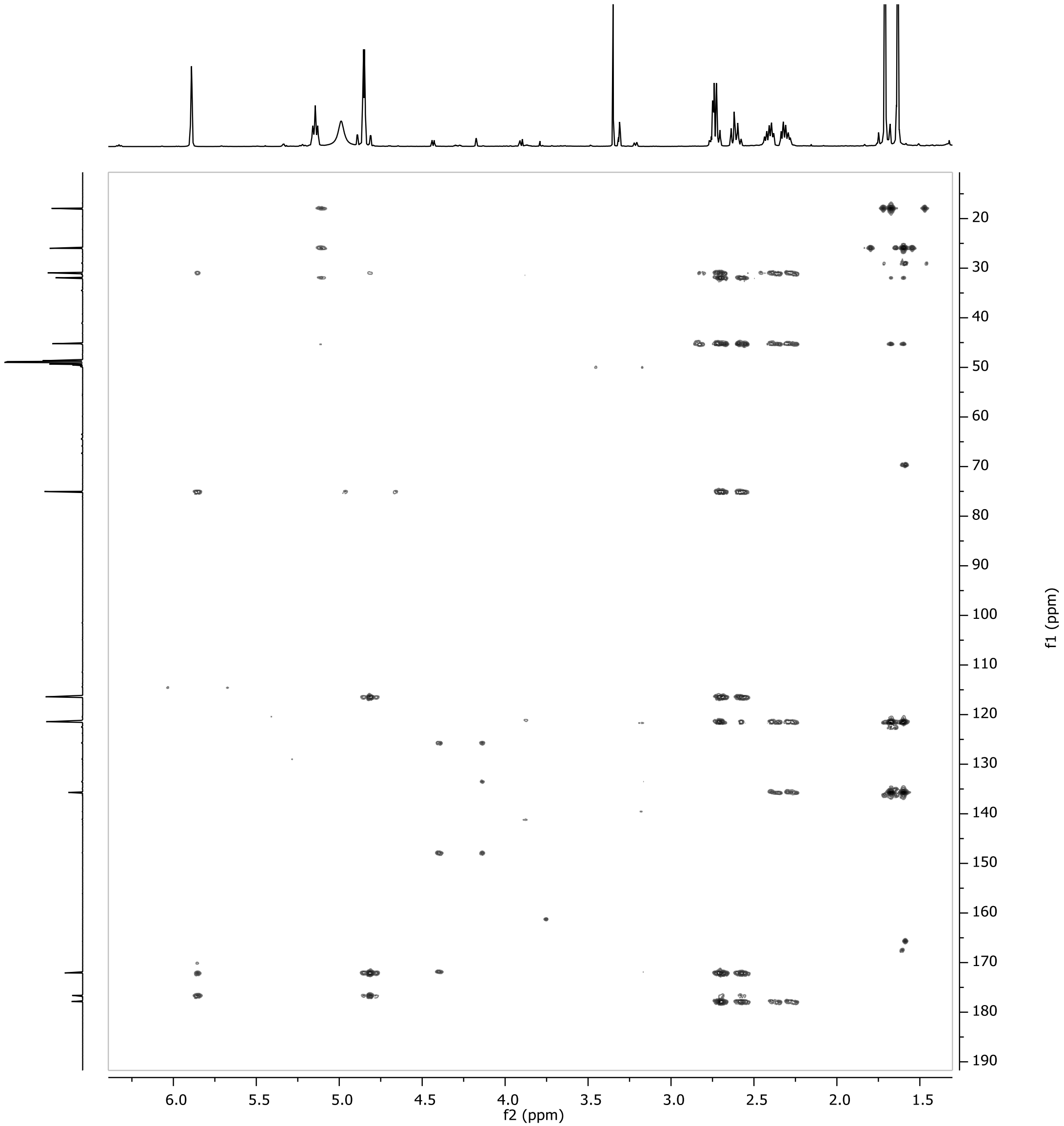

Figure S14. HMBC spectrum of **2** in methanol-d_4 at 500 MHz.

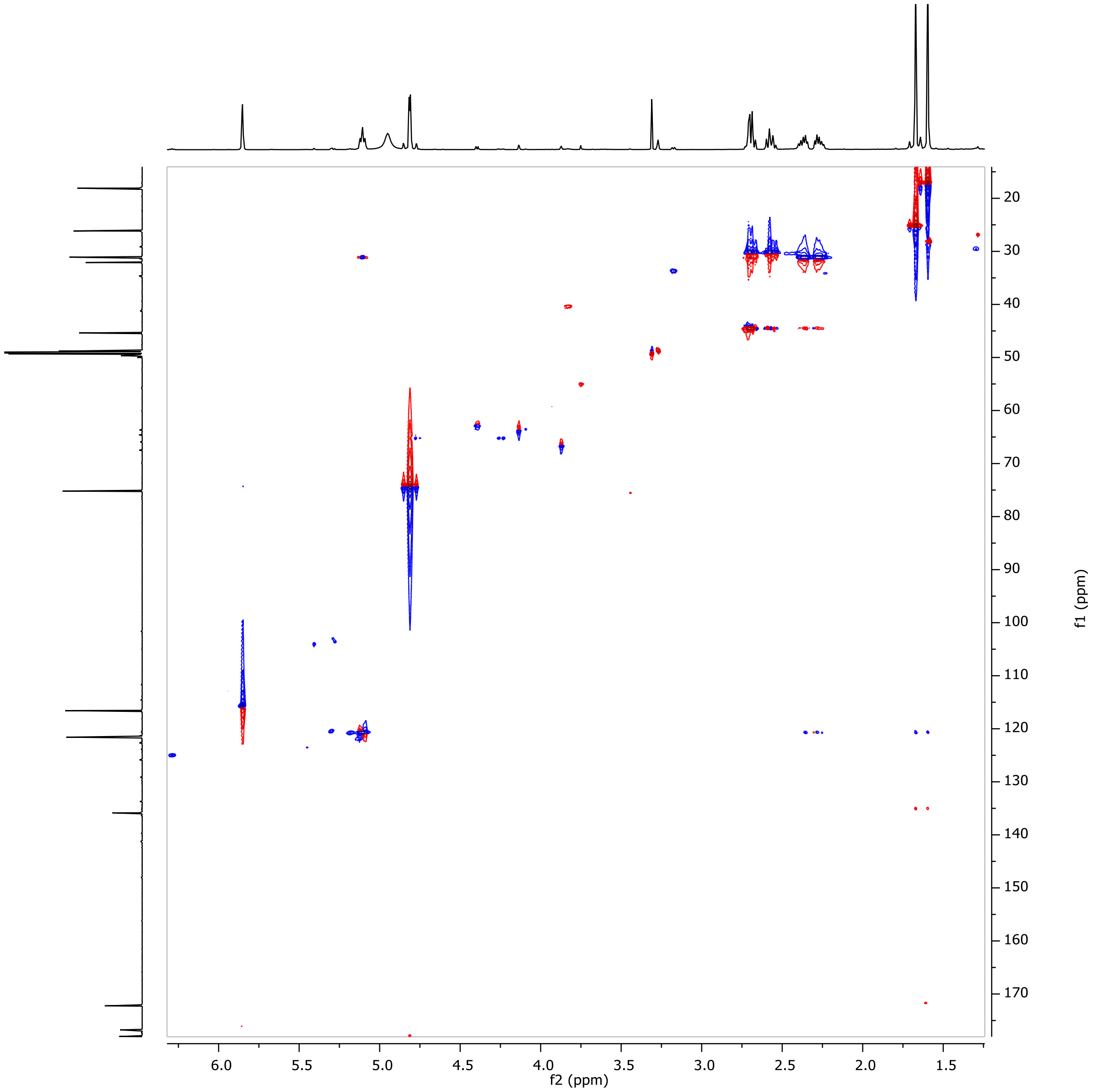

Figure S15. HSQC spectrum of **2** in methanol-d_4 at 500 MHz.

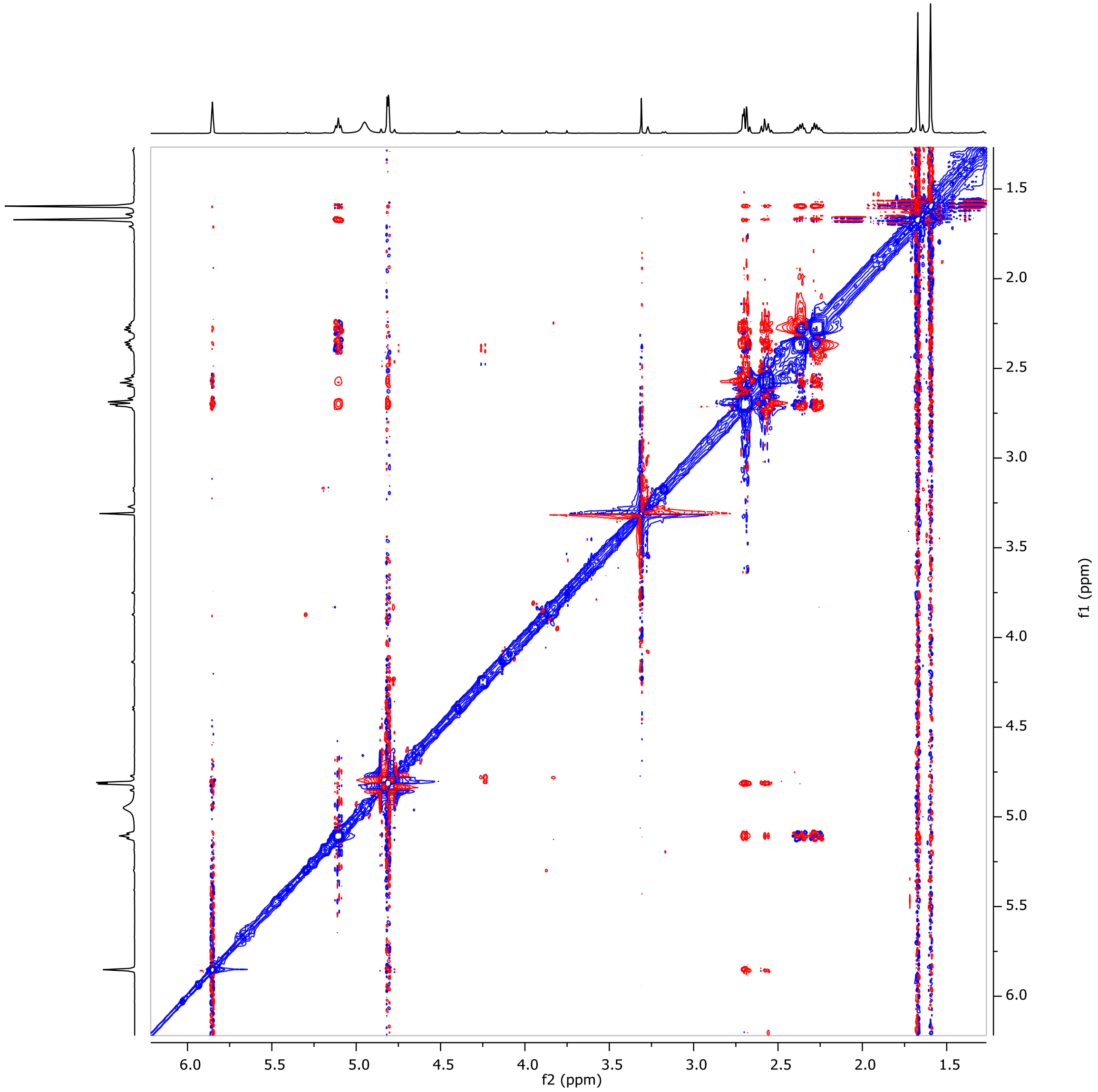

Figure S16. ROESY spectrum of **2** in methanol-d_4 at 500 MHz.

Generic Display Report

Analysis Info

Analysis Name	S:\PEOPLE\sel22_Sherif Elsayed\Bondarzewia\AmaZon\IHI 766 R2F9F2_RC2_01_50467.d	Acquisition Date	07.09.2023 18:47:20
Method	50467.m	Operator	tti
Sample Name	IHI 766 R2F9F2	Instrument	amaZon speed
Comment			

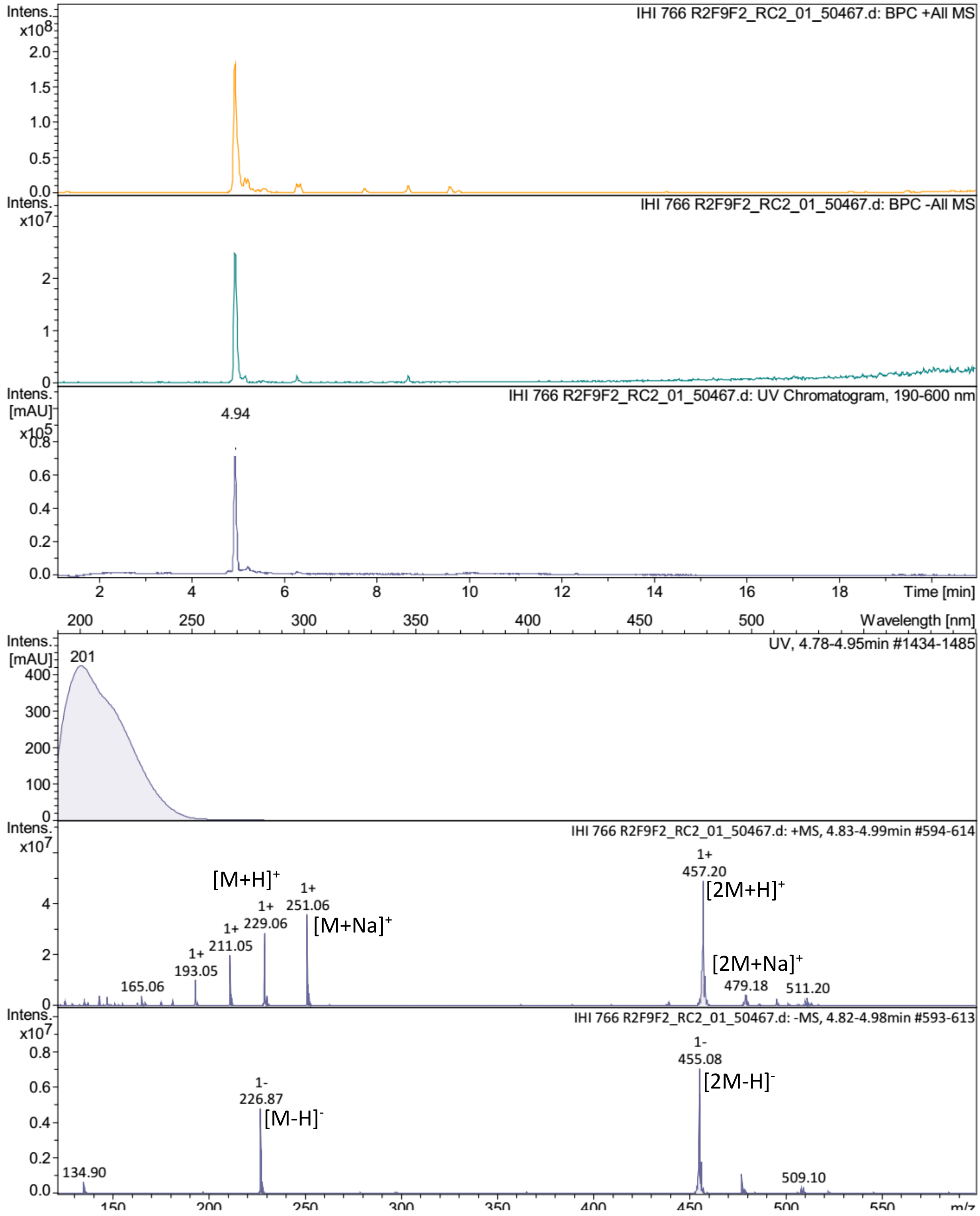

Bruker Compass DataAnalysis 4.4 printed: 11.10.2023 17:38:02 by: sel22 Page 1 of 1

Figure S17. LRESIMS of **3**.

Generic Display Report

Analysis Info

Analysis Name	S:\PEOPLE\sel22_Sherif Elsayed\Bondarzewia\MaXis\IHI 766 R1F8_24_01_13120.d	Acquisition Date	05.09.2023 18:49:37
Method	pos_säure_10000_screening_ms_100_2500_line.m	Operator	ate06
Sample Name	IHI 766 R1F8	Instrument	maXis
Comment	Screening01<br>Waters Acquity UPLC BEH C_18 1,7um 2.1x50mm		

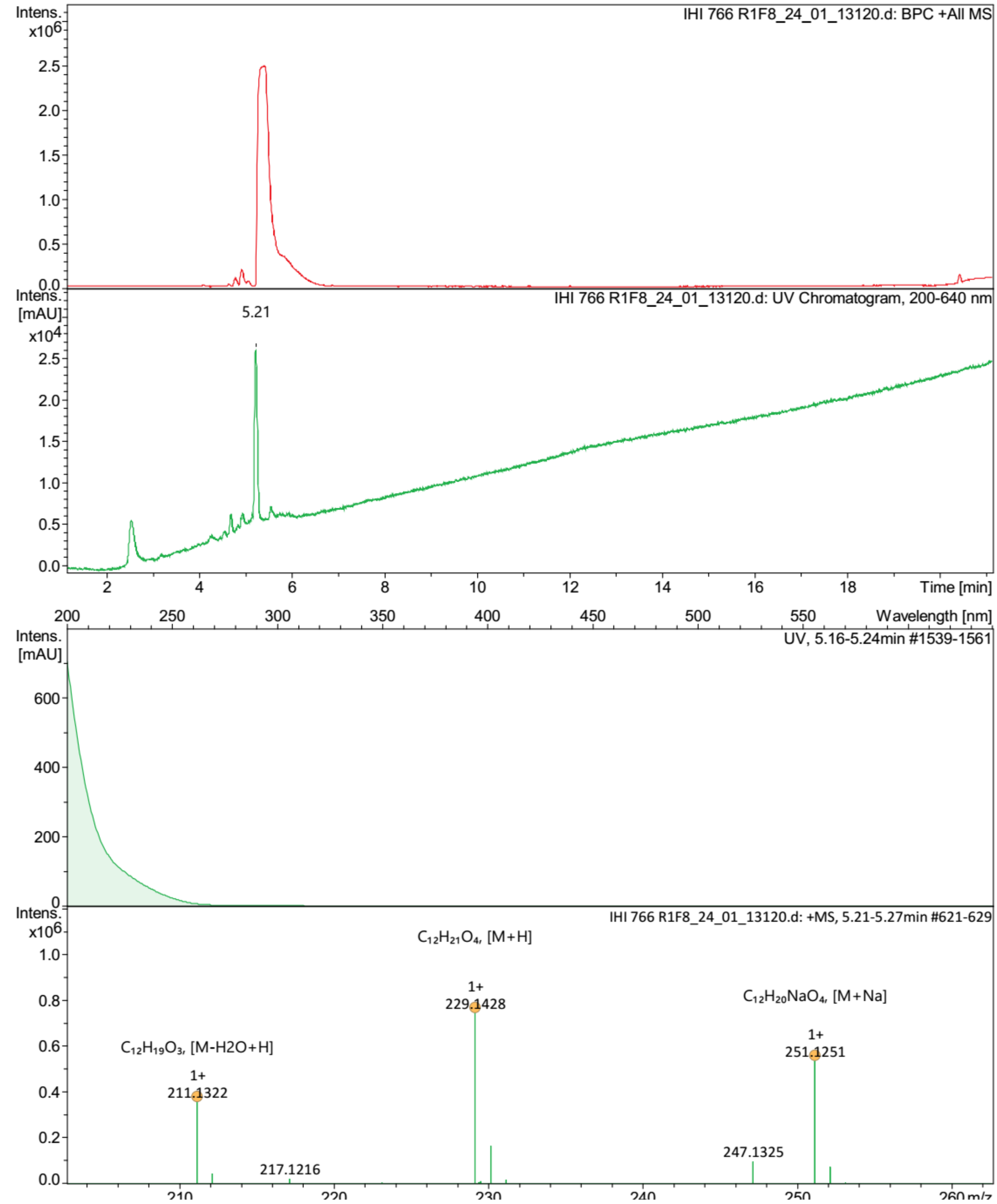

Figure S18. HRESIMS of **3**.

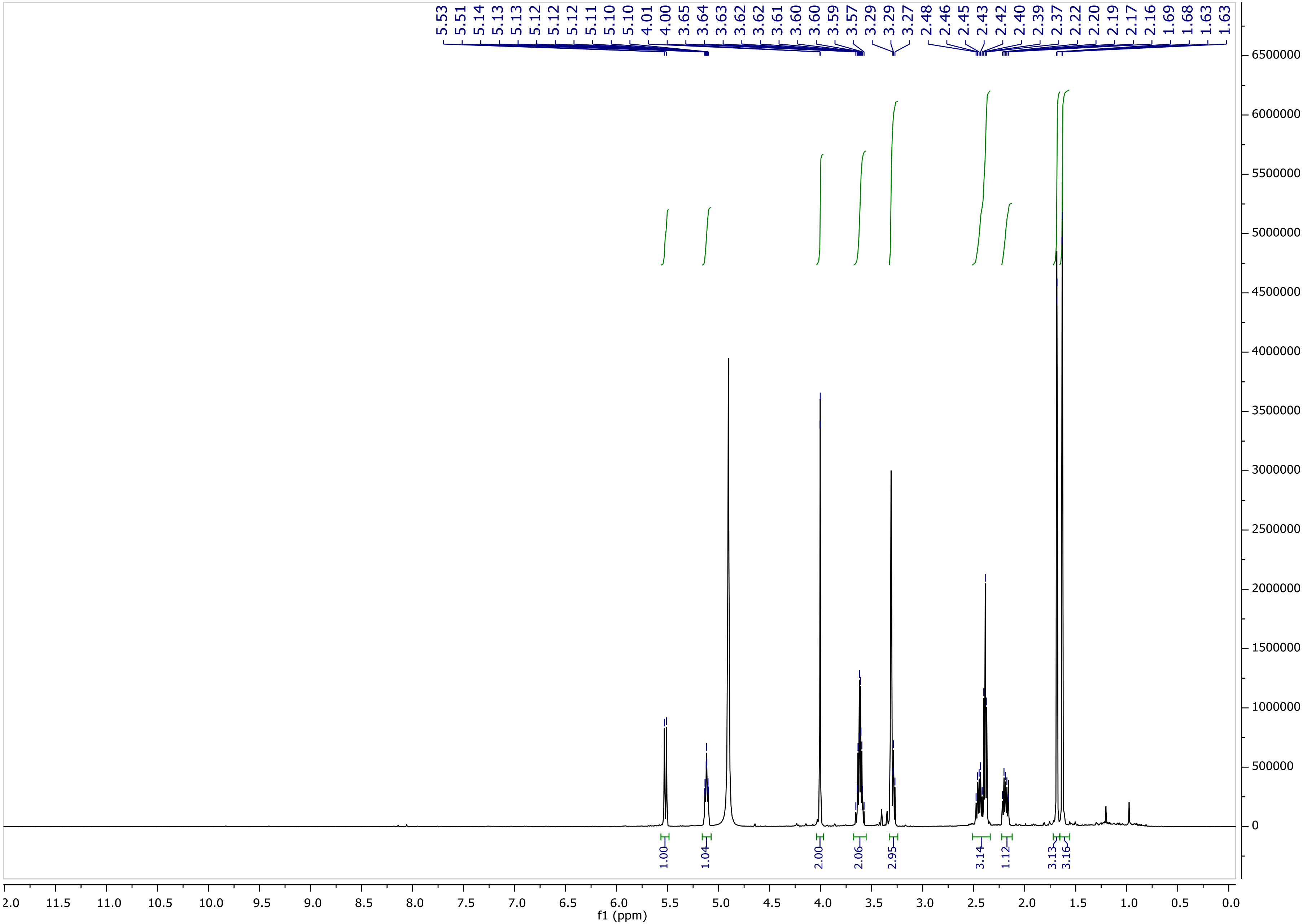

Figure S19. ^{1}H NMR spectrum of **3** in methanol-d_4 at 500 MHz.

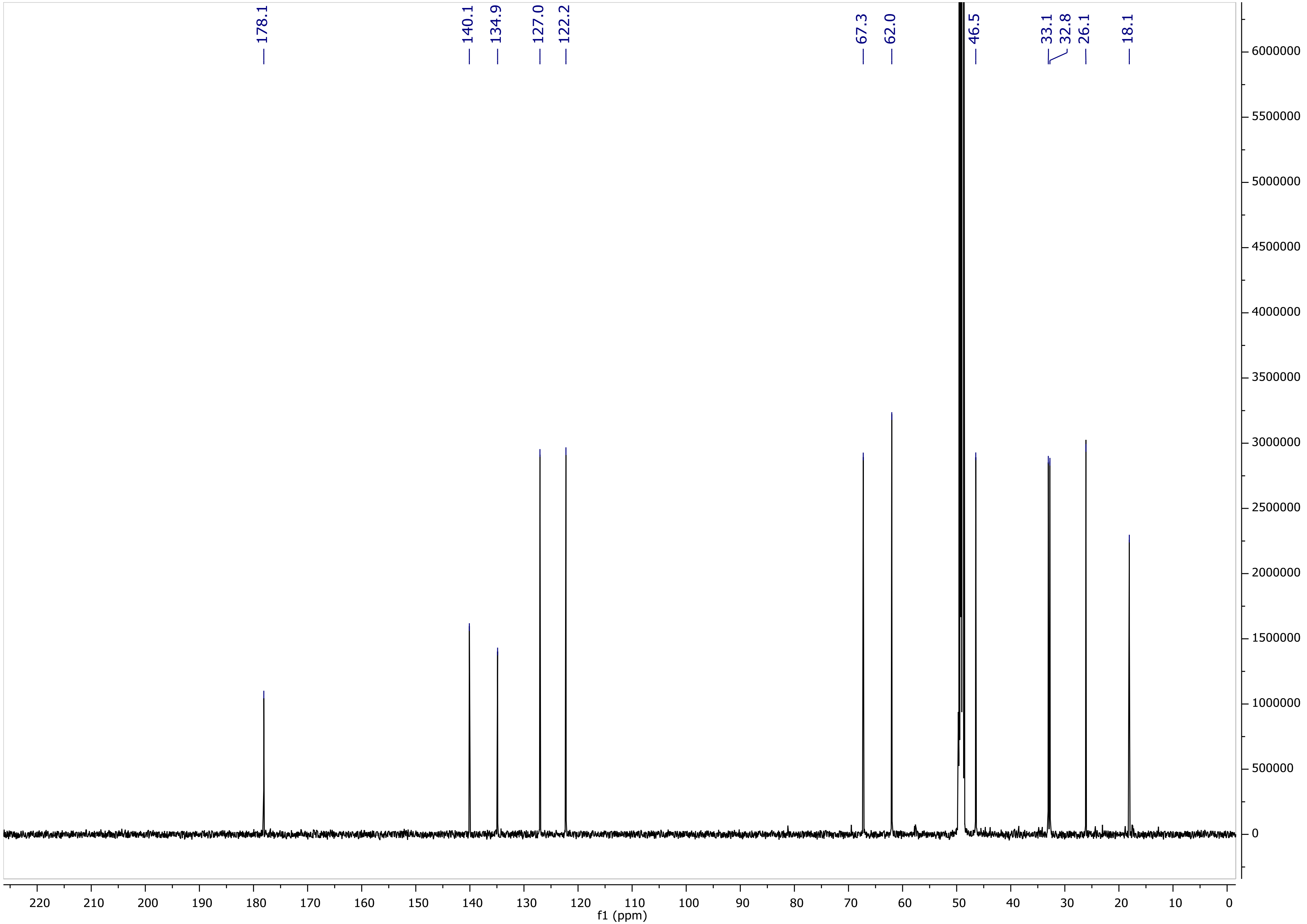

Figure S20. ^{13}C NMR spectrum of **3** in methanol-d_4 at 125 MHz.

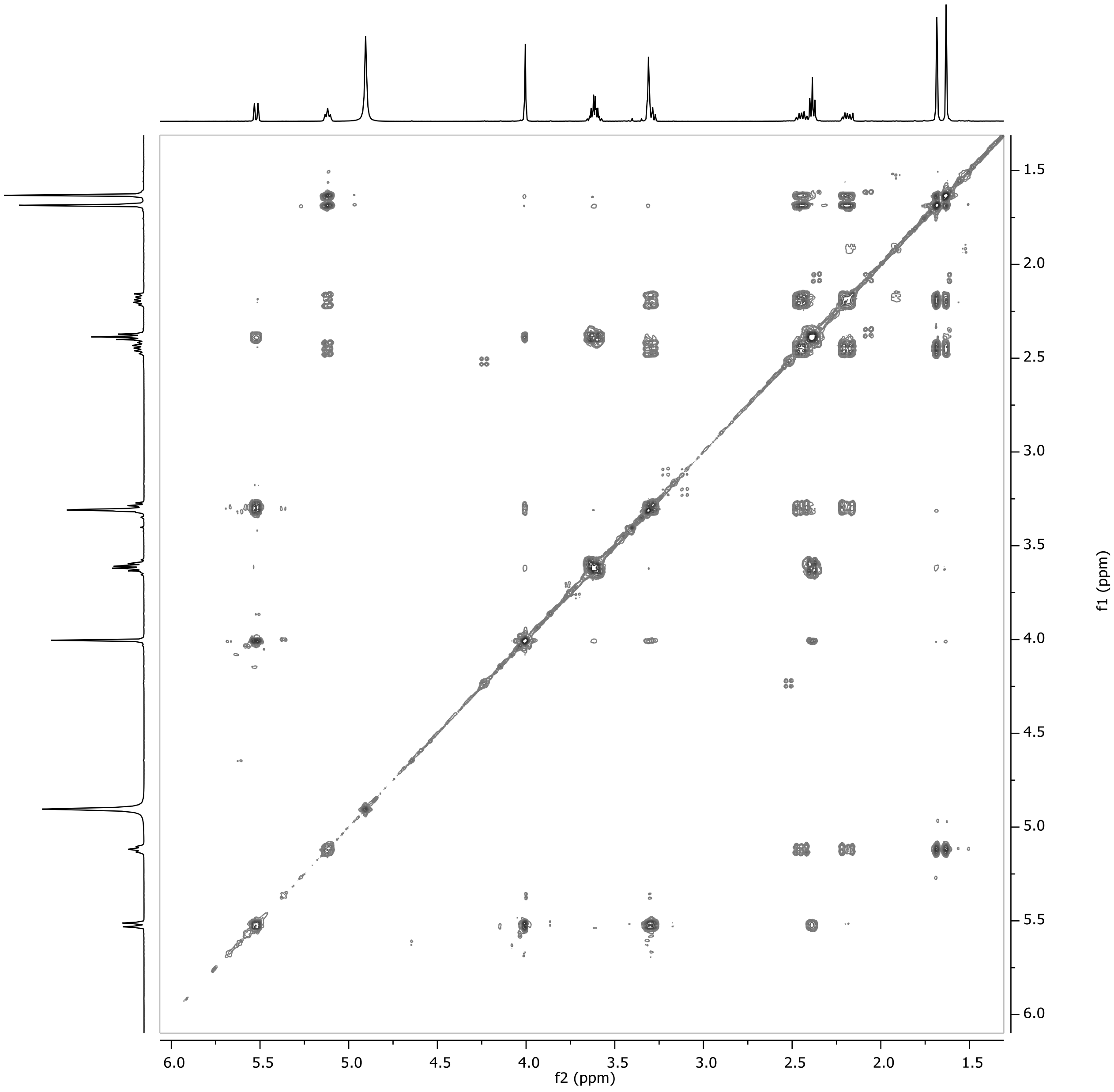

Figure S21. ^{1}H-^{1}H COSY spectrum of **3** in methanol-d_4 at 500 MHz.

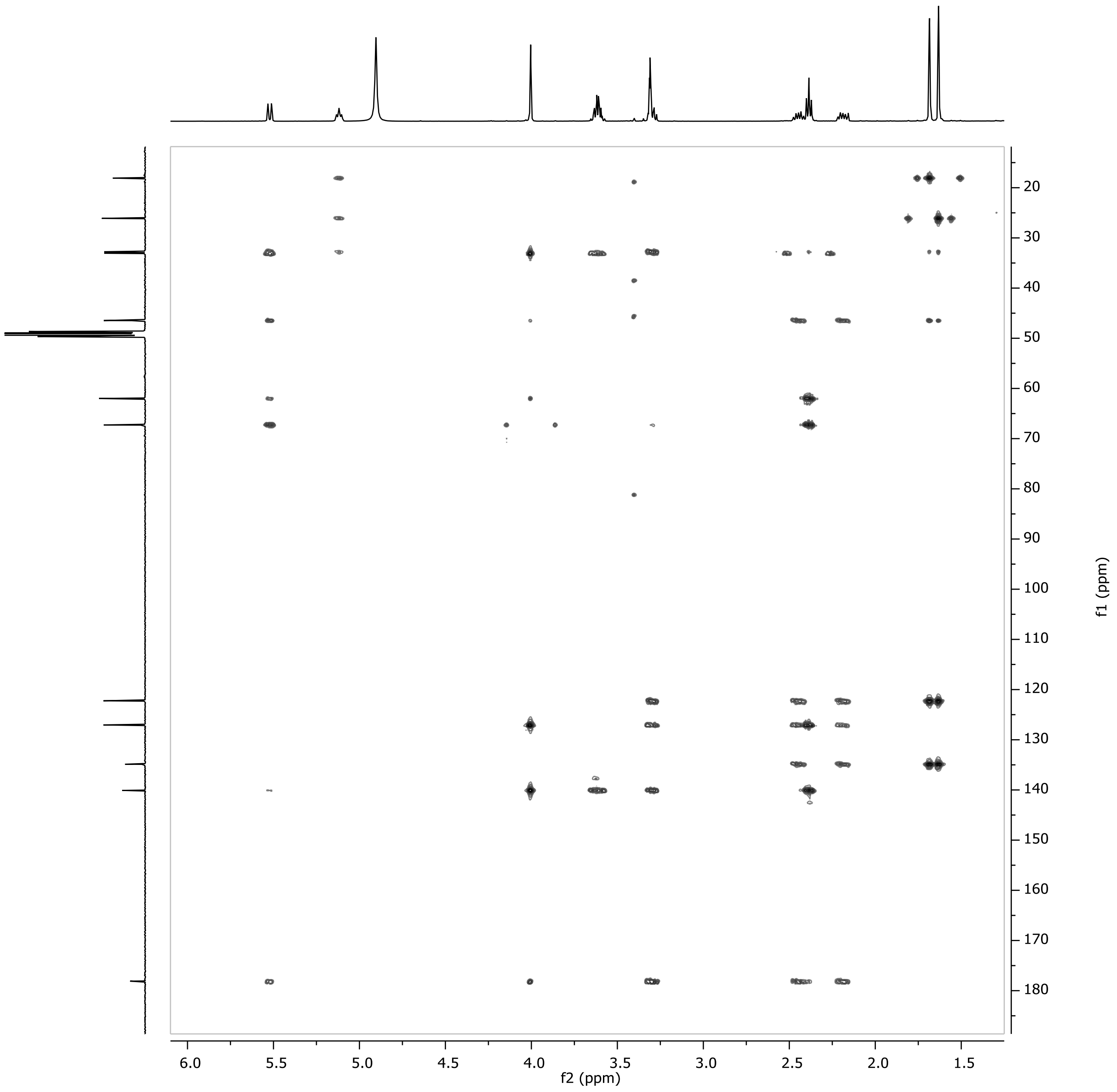

Figure S22. HMBC spectrum of **3** in methanol-d_4 at 500 MHz.

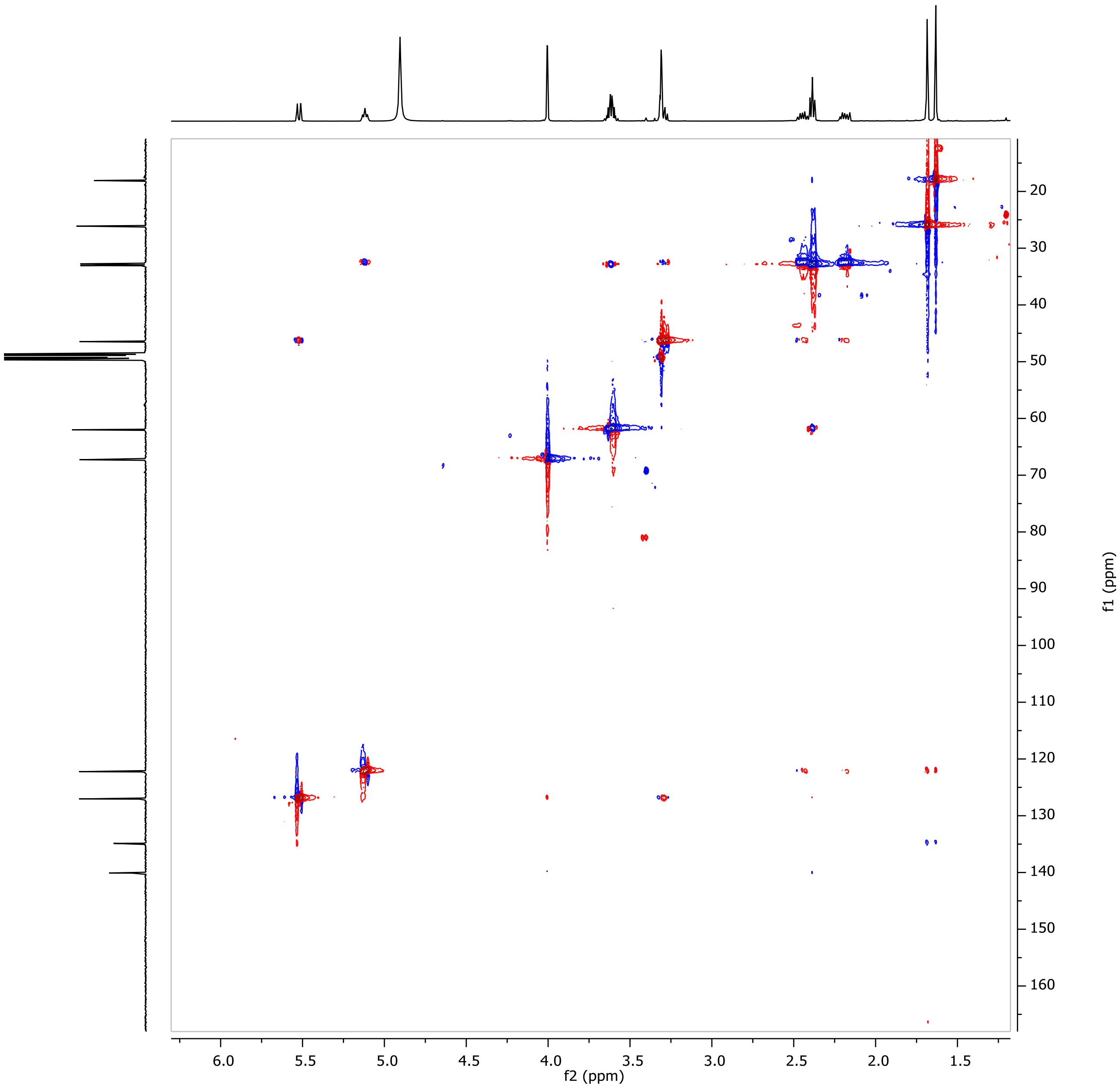

Figure S23. HSQC spectrum of **3** in methanol-d_4 at 500 MHz.

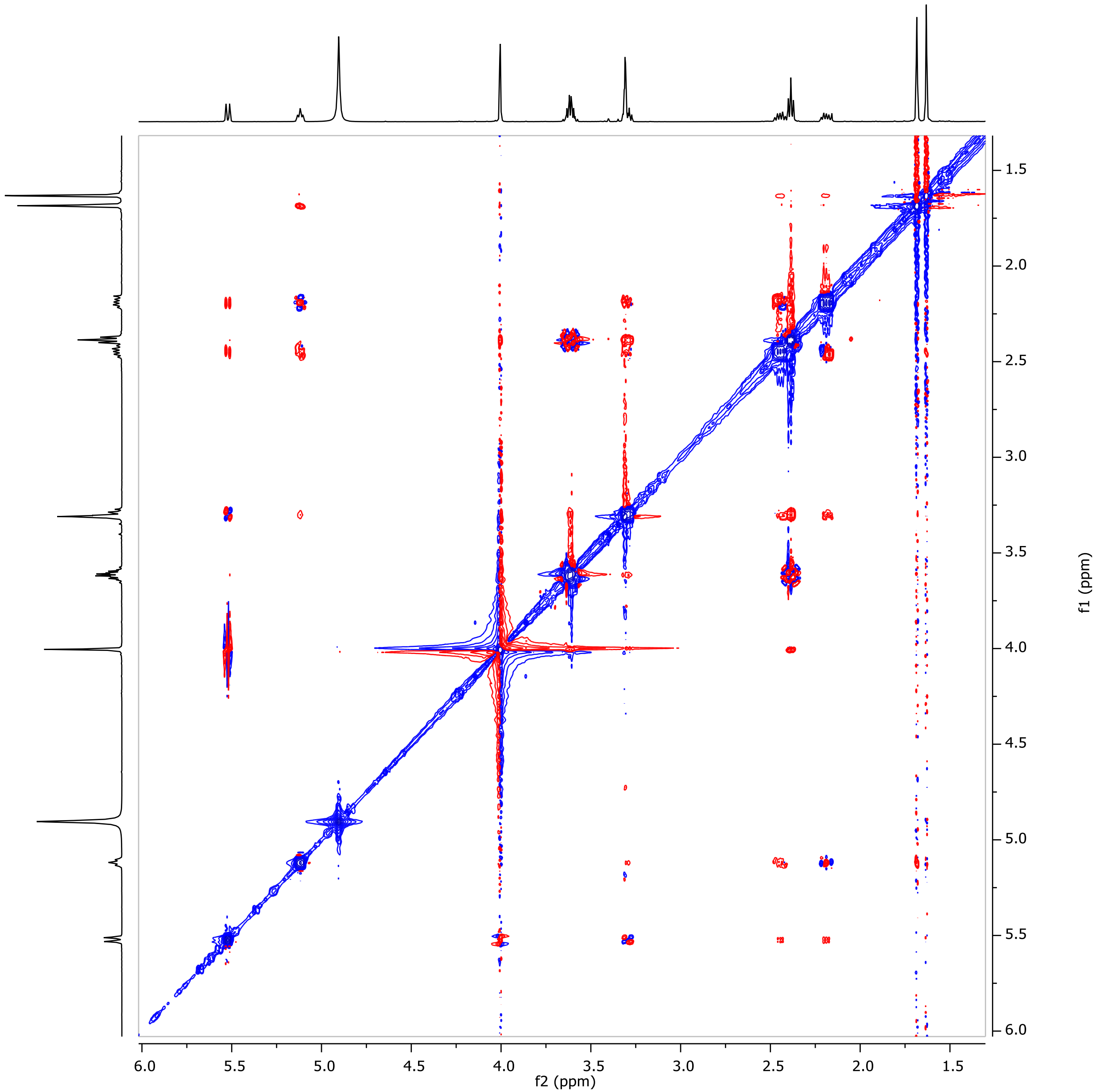

Figure S24. ROESY spectrum of **3** in methanol-d_4 at 500 MHz.

Generic Display Report

Analysis Info

Analysis Name	S:\PEOPLE\sel22_Sherif Elsayed\Bondarzewia\AmaZon\IHI 766R2F2_GD2_01_50334.d	Acquisition Date	02.09.2023 03:40:03
Method	50334.m	Operator	tti
Sample Name	IHI 766R2F2	Instrument	amaZon speed
Comment			

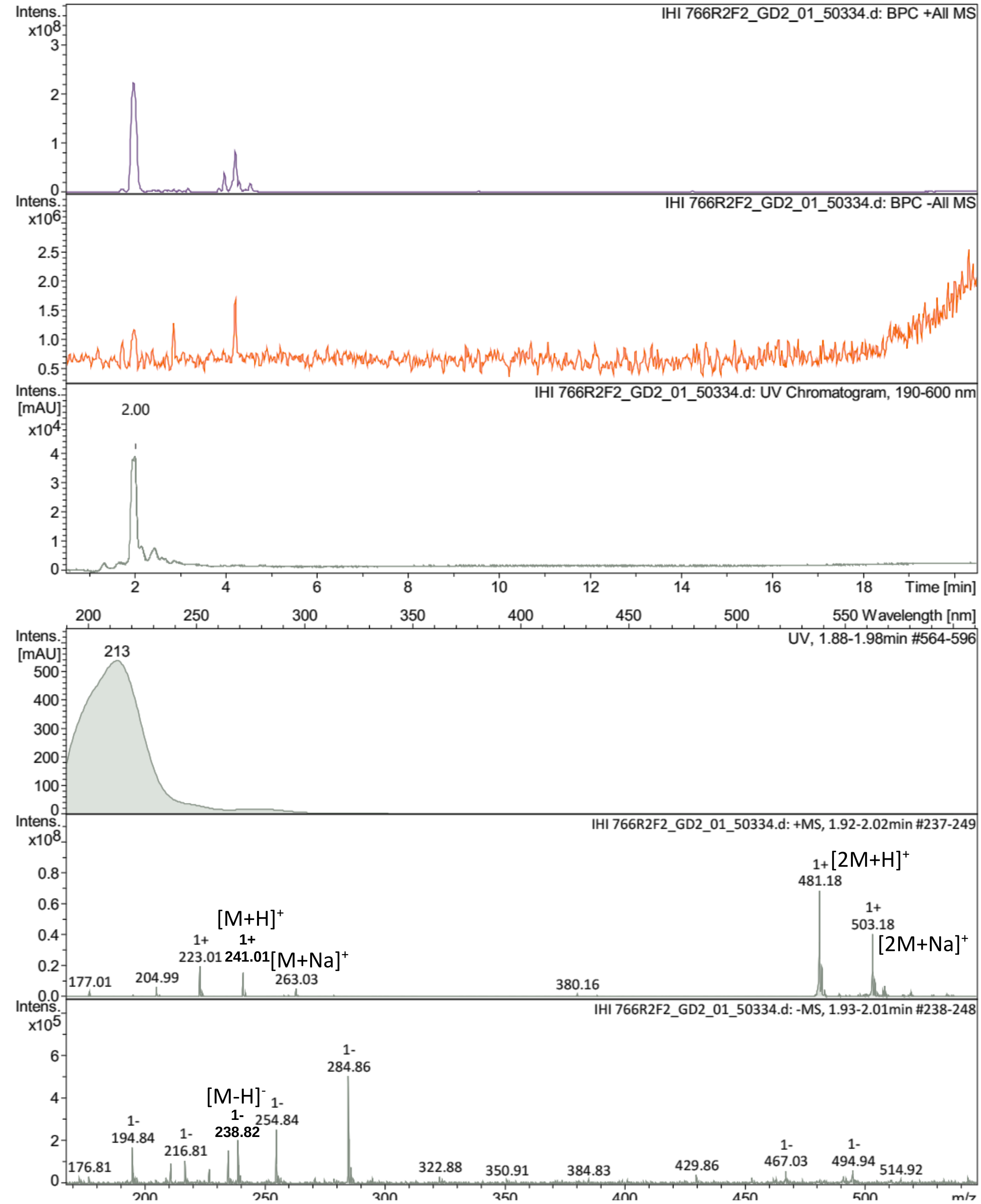

Bruker Compass DataAnalysis 4.4 printed: 11.10.2023 17:47:16 by: sel22 Page 1 of 1

Figure S25. LRESIMS of **4**.

Generic Display Report

Analysis Info

Analysis Name	F:\Volume D\HZI Projects\Winnie\8-Bondarzewia mesenterica\Bondarzewia\MaXis\IHI 766 R2F2_23_01_13119.d	Acquisition Date	05.09.2023 18:18:39
Method	[illegible]screening_ms_100_2500_line.m	Operator	ate06
Sample Name	IHI 766 R2F2	Instrument	maXis
Comment	Screening01<br>Waters Acquity UPLC BEH C_18 1,7um 2.1x50mm		

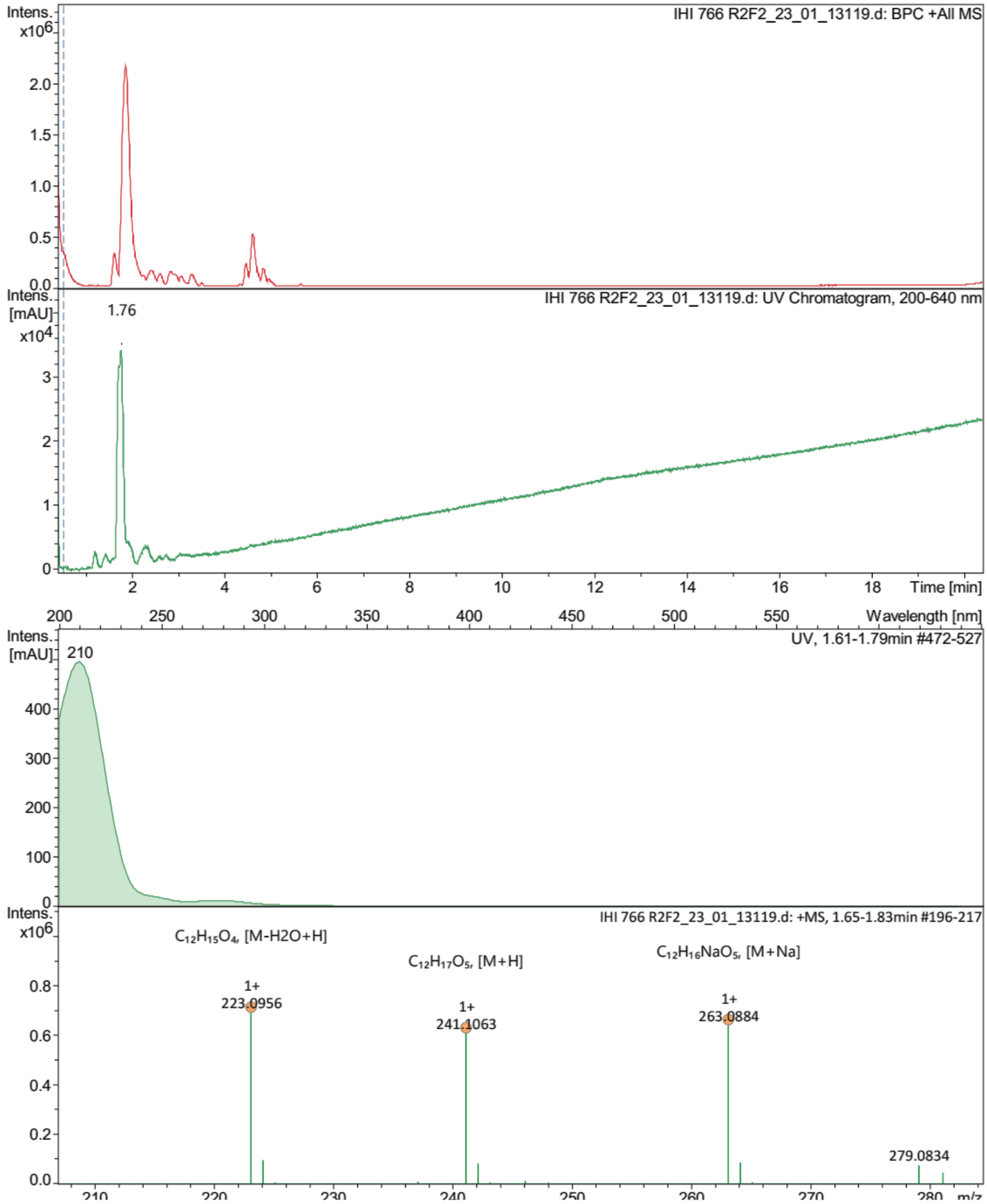

Figure S26. HRESIMS of **4**.

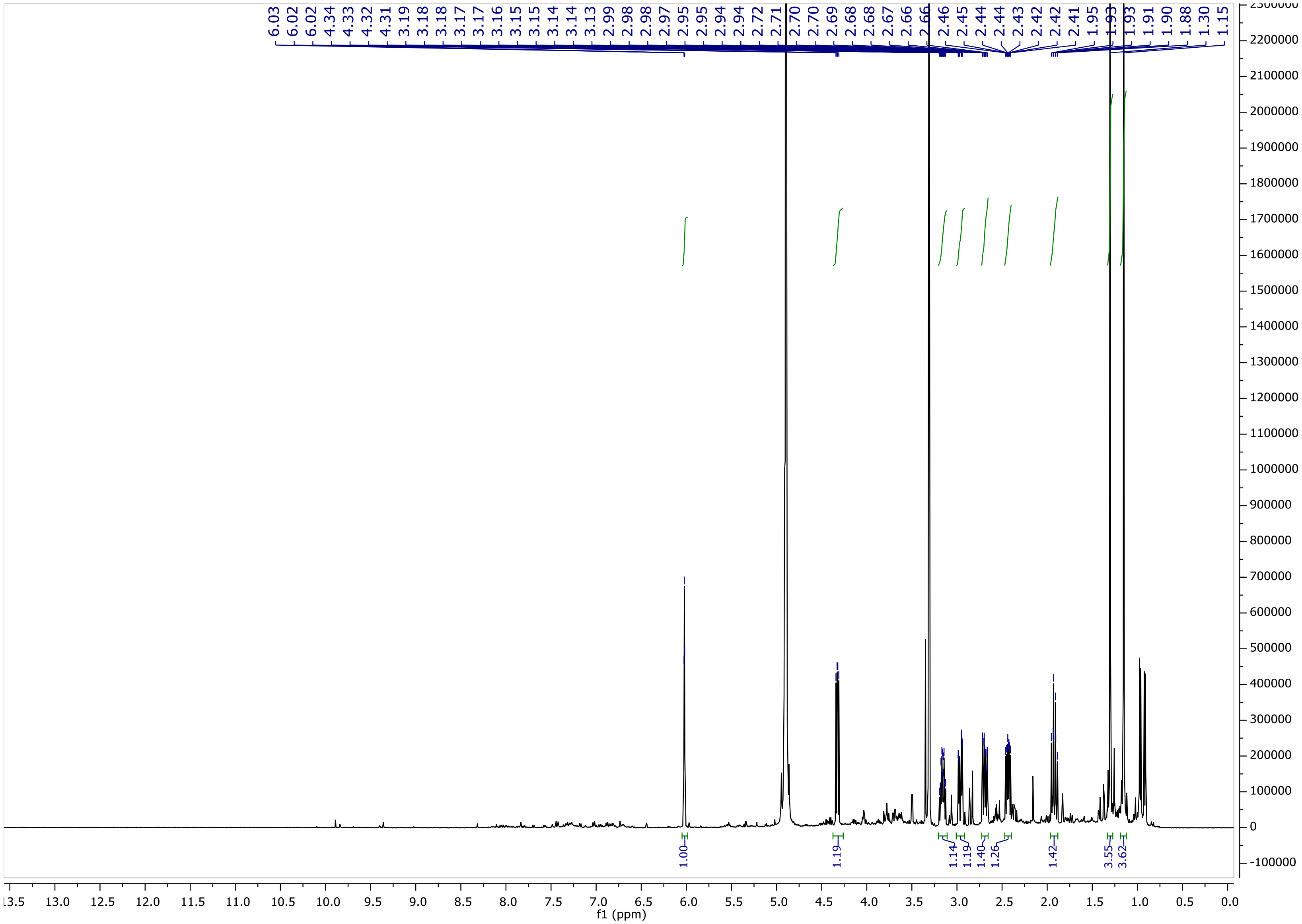

Figure S27. ^{1}H NMR spectrum of **4** in methanol-d_4 at 500 MHz.

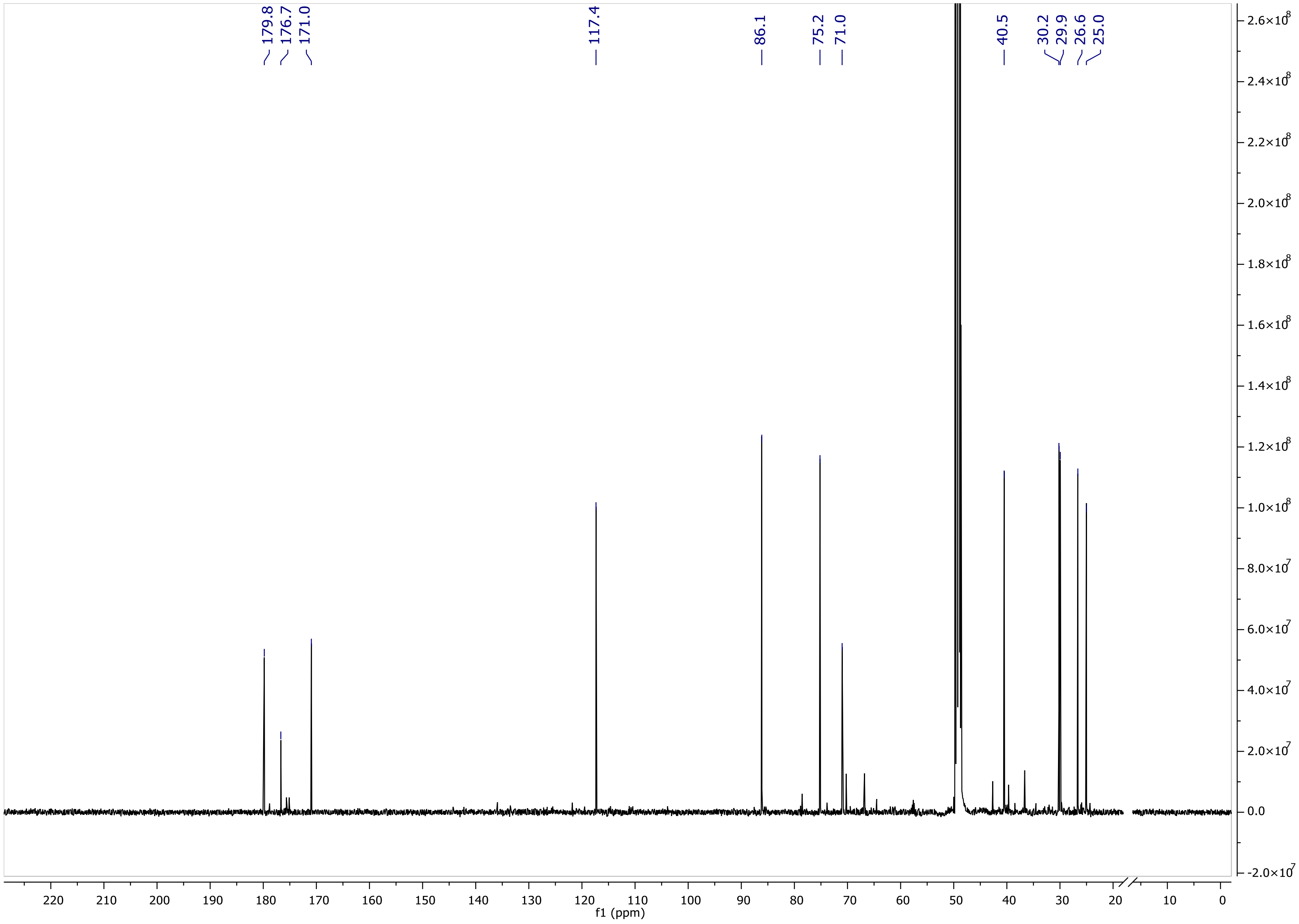

Figure S28. ^{13}C NMR spectrum of **4** in methanol-d_4 at 125 MHz.

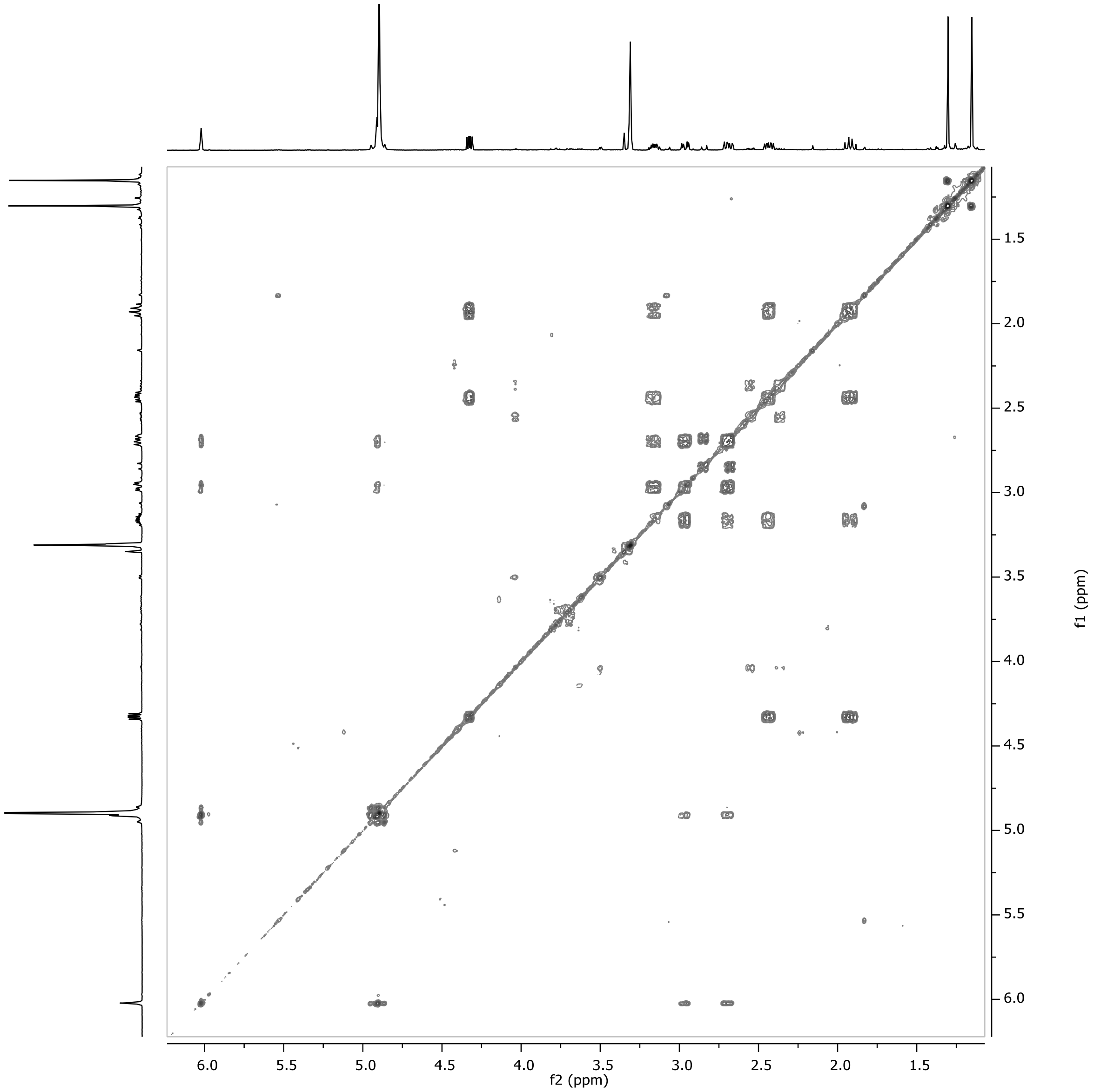

Figure S29. ^1H-^1H COSY spectrum of **4** in methanol-d_4 at 500 MHz.

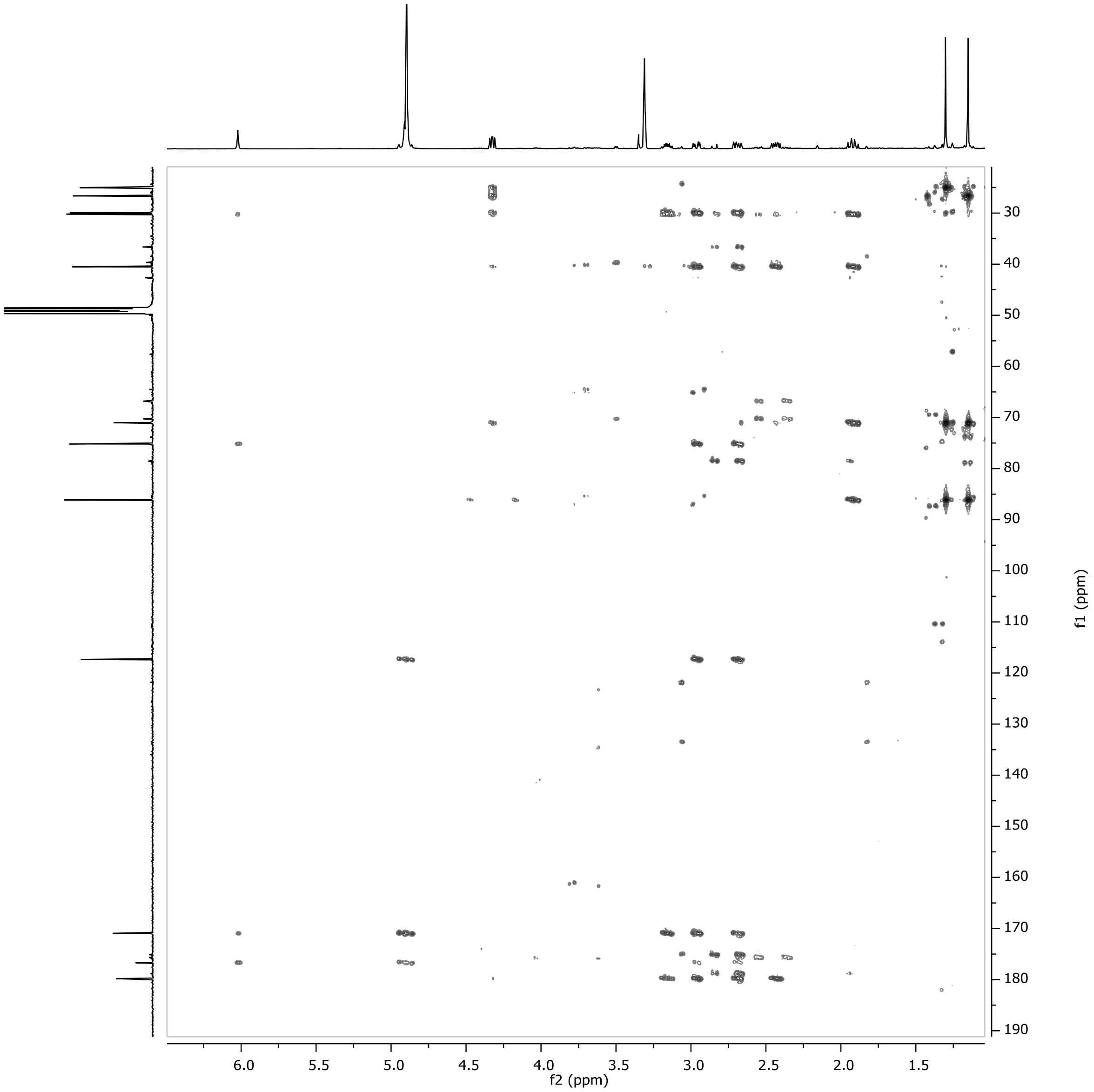

Figure S30. HMBC spectrum of **4** in methanol-d_4 at 500 MHz.

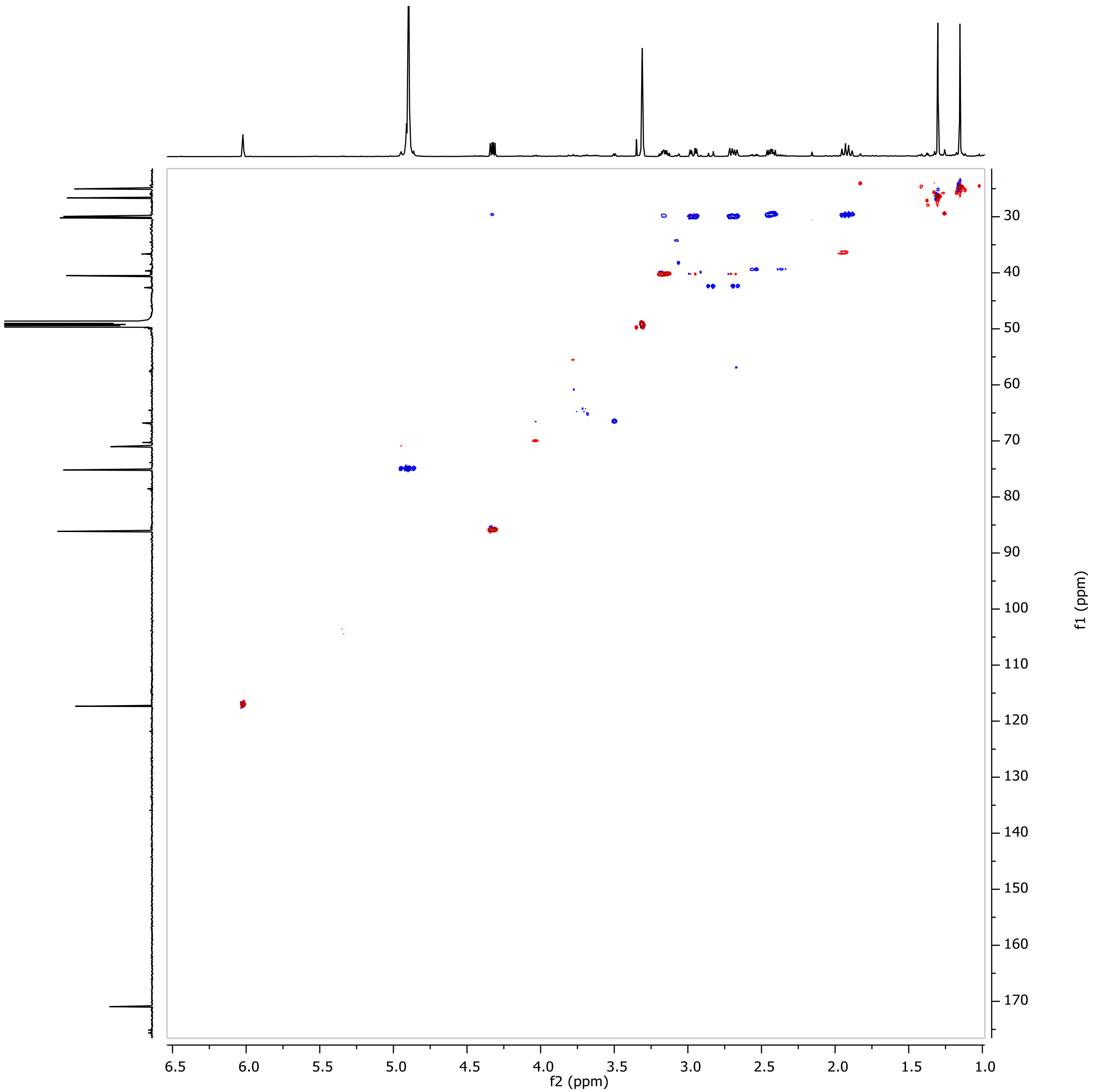

Figure S31. HSQC spectrum of **4** in methanol-d_4 at 500 MHz.

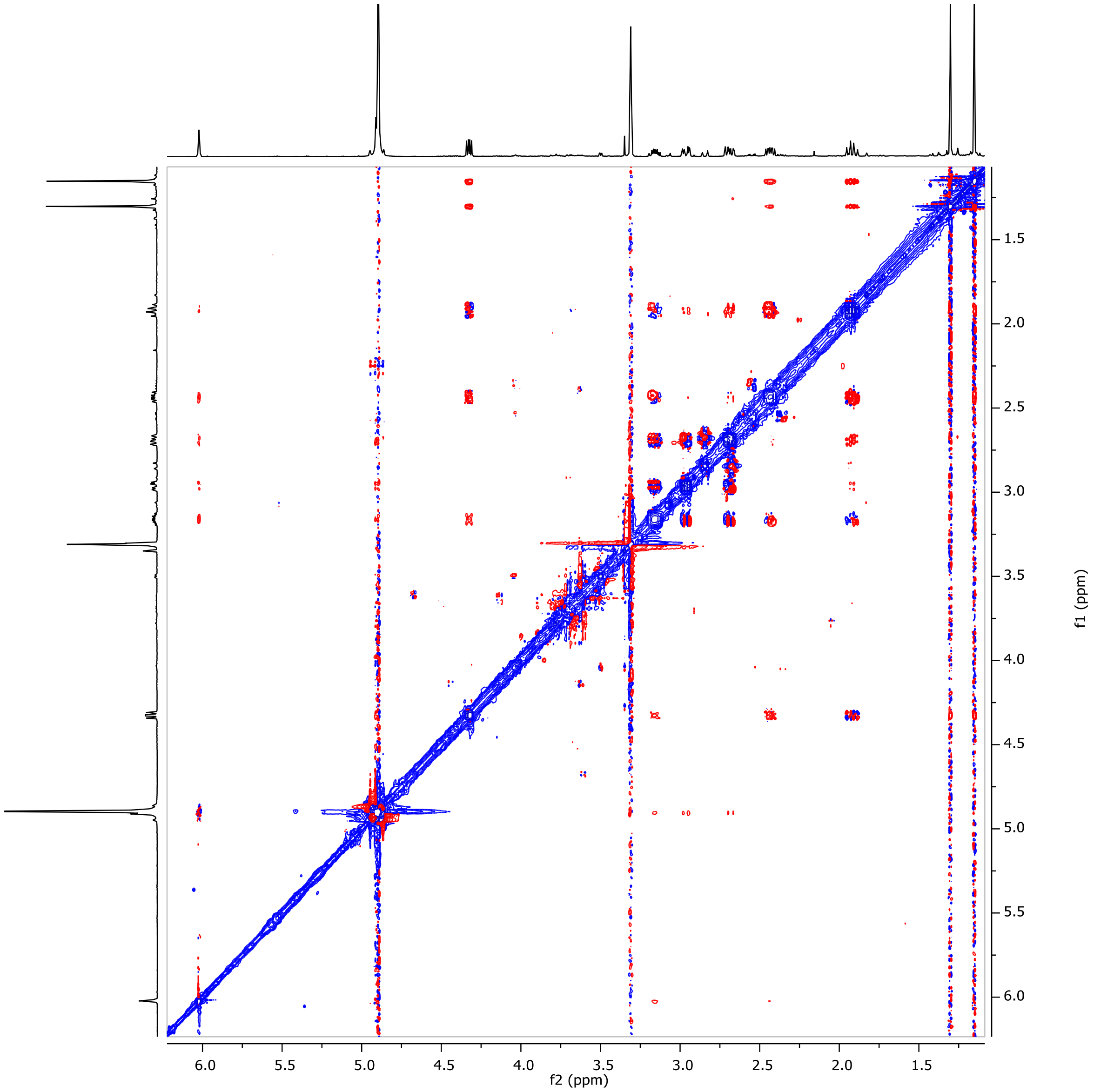

Figure S32. ROESY spectrum of **4** in methanol-d_4 at 500 MHz.

Generic Display Report

**Analysis Info**		Acquisition Date	02.09.2023 04:16:16
Analysis Name	S:\PEOPLE\sel22_Sherif Elsayed\Bondarzewia\AmaZon\IHI 766R2F3_GD3_01_50335.d		
Method	50335.m	Operator	tti
Sample Name	IHI 766R2F3	Instrument	amaZon speed
Comment			

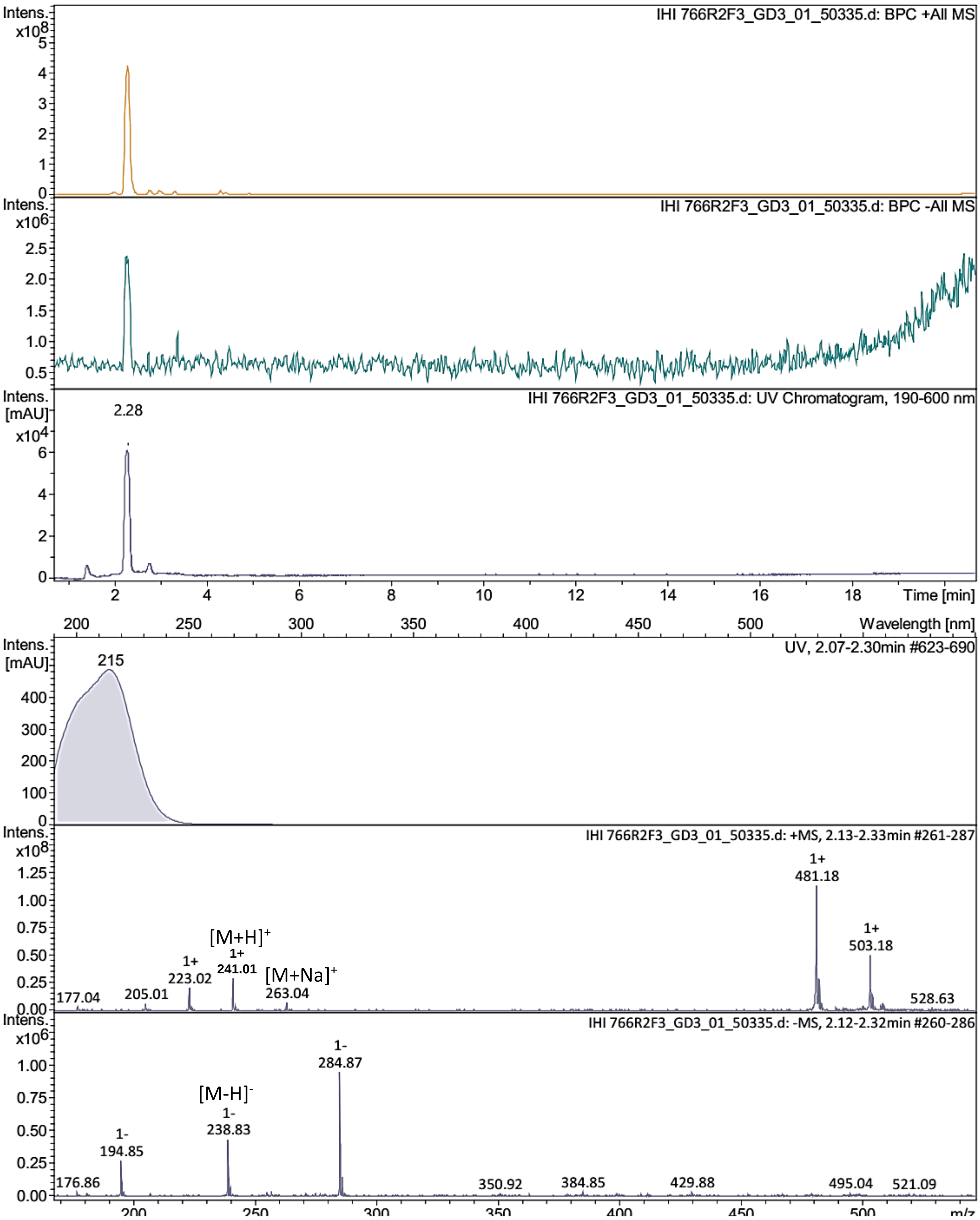

Figure S33. LRESIMS of **5**.

Generic Display Report

Analysis Info

Analysis Name	F:\Volume D\HZI Projects\Winnie\8-Bondarzewia mesenterica\Bondarzewia\MaXis\IHI 766 R2F3_21_01_13117.d	Acquisition Date	05.09.2023 17:16:45
Method	pos_säure_[illegible]_screening_ms_100_2500_line.m	Operator	ate06
Sample Name	IHI 766 R2F3	Instrument	maXis
Comment	Screening01<br>Waters Acquity UPLC BEH C_18 1,7um 2.1x50mm		

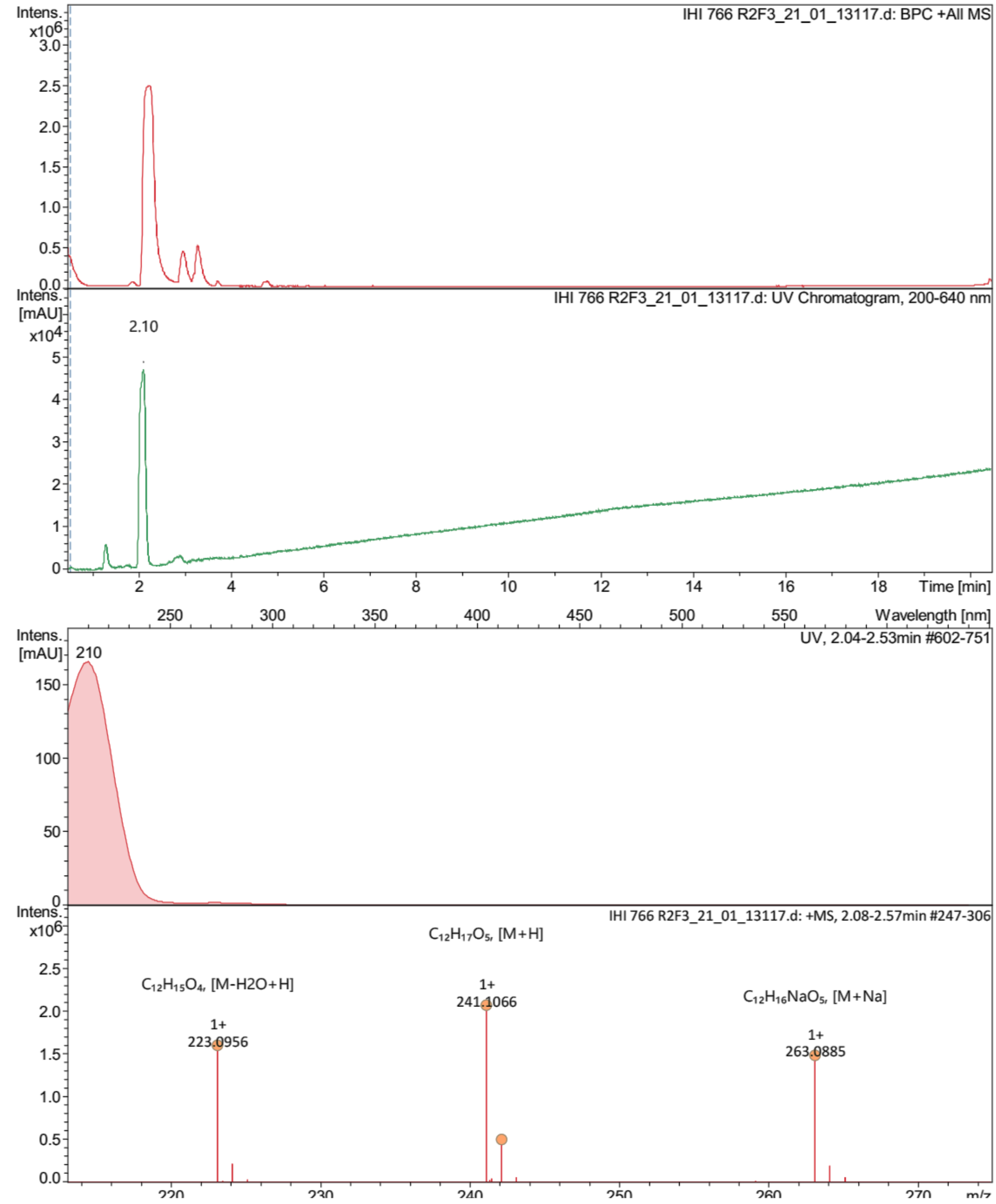

Bruker Compass DataAnalysis 4.4 printed: 11.10.2023 16:06:36 by: sel22 Page 1 of 1

Figure S34. HRESIMS of **5**.

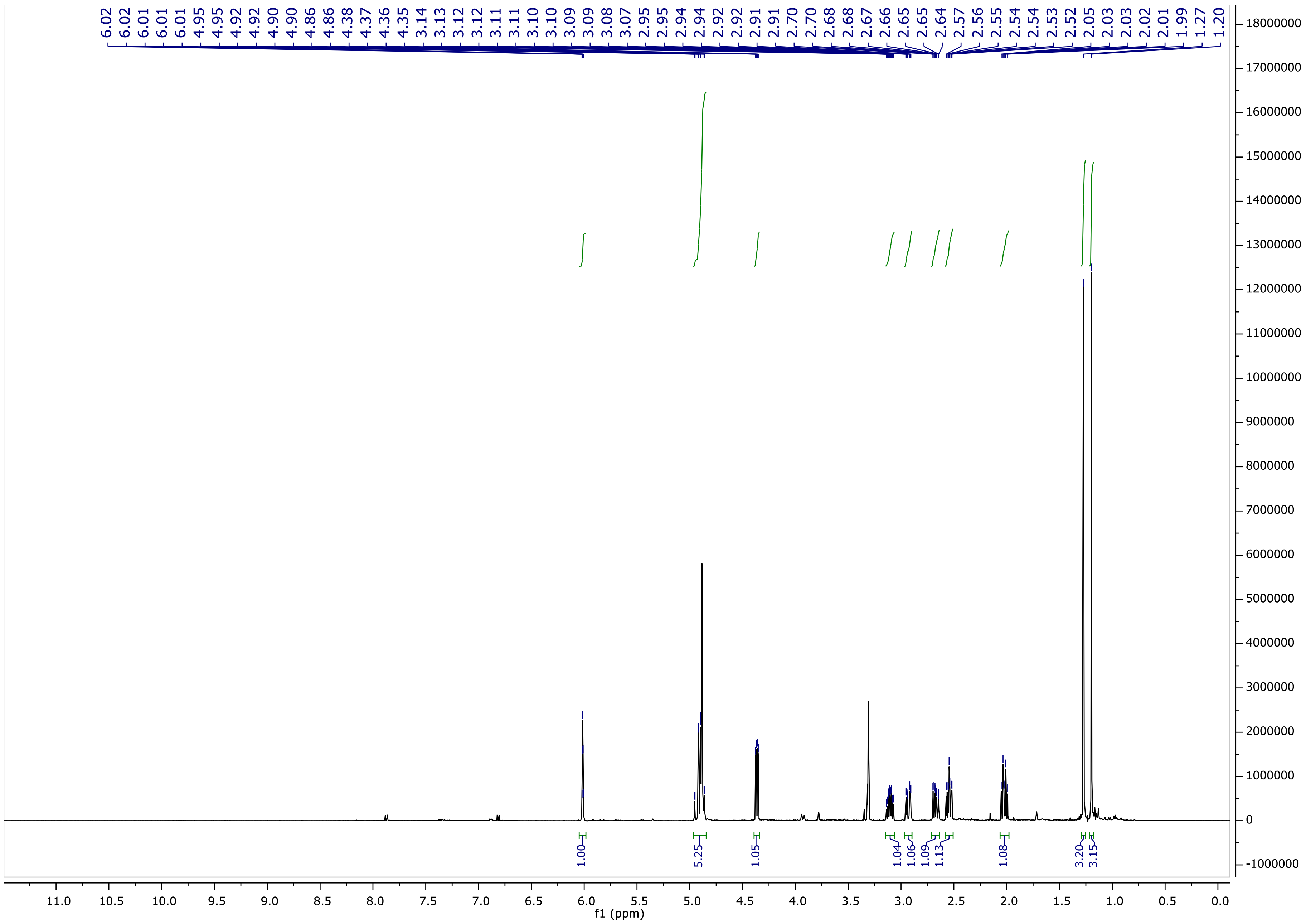

Figure S35. ^{1}H NMR spectrum of **5** in methanol-d_4 at 500 MHz.

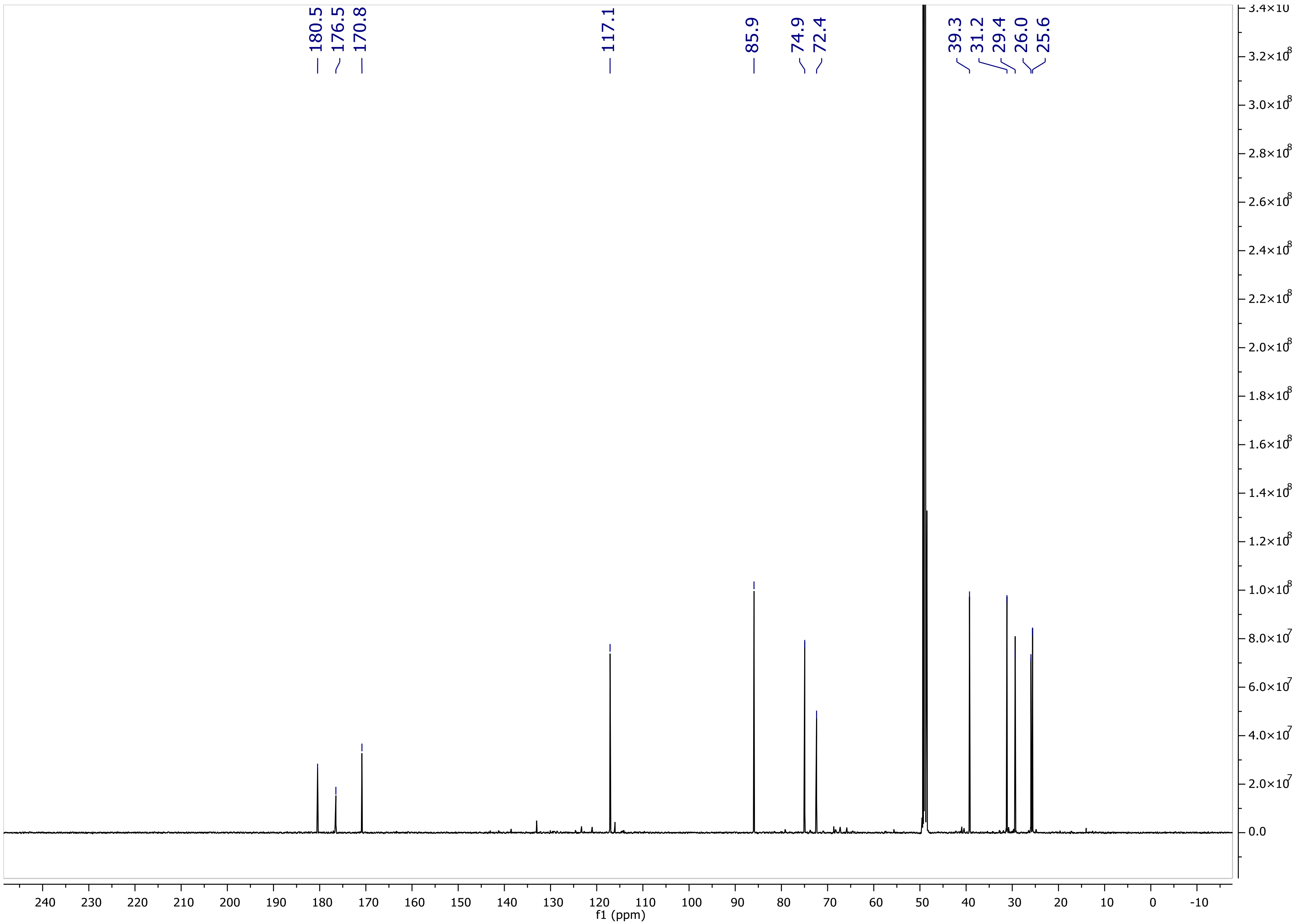

Figure S36. ^{13}C NMR spectrum of **5** in methanol-*d*$_4$ at 125 MHz.

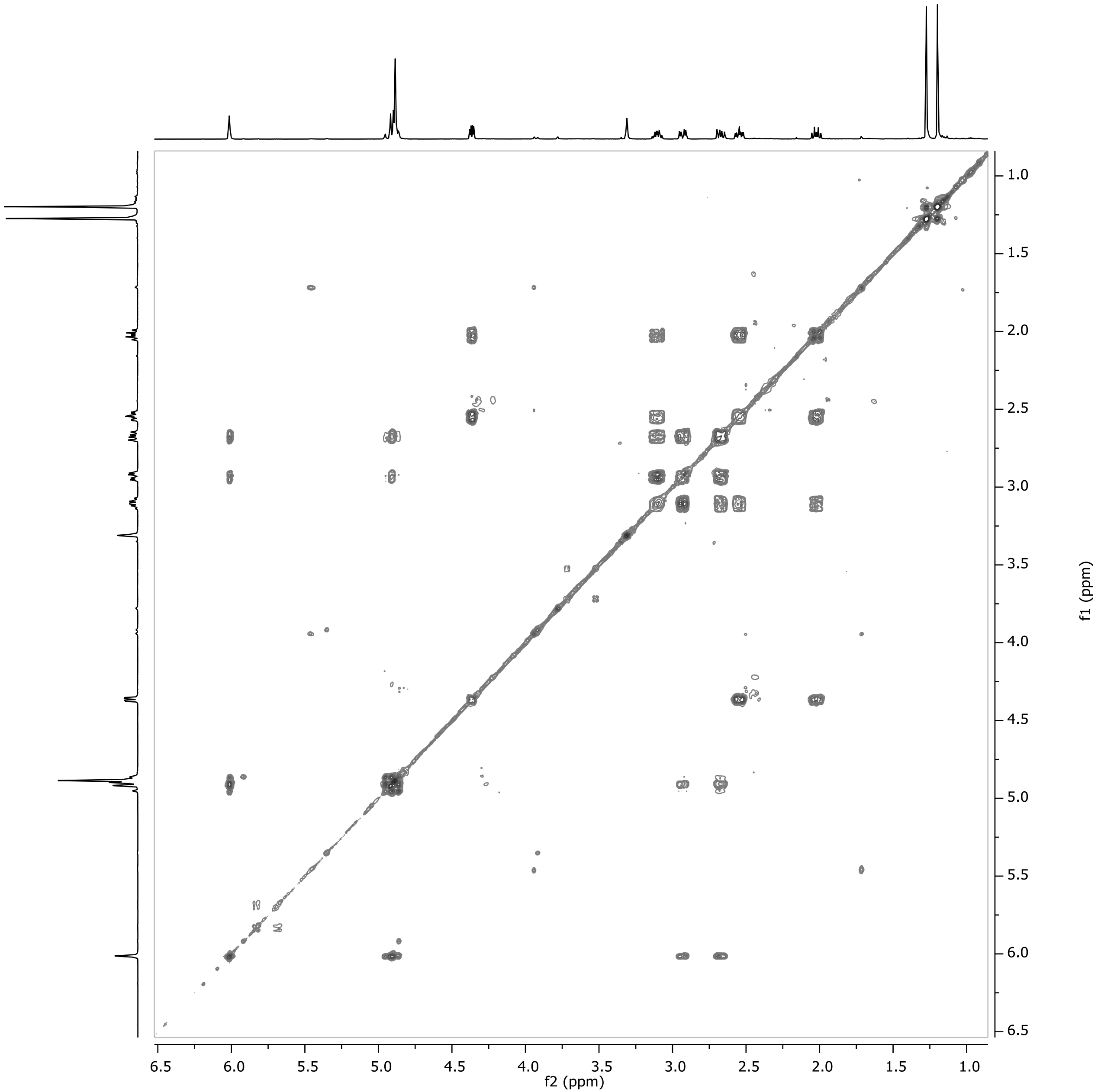

Figure S37. ^1H-^1H COSY spectrum of **5** in methanol-d_4 at 500 MHz.

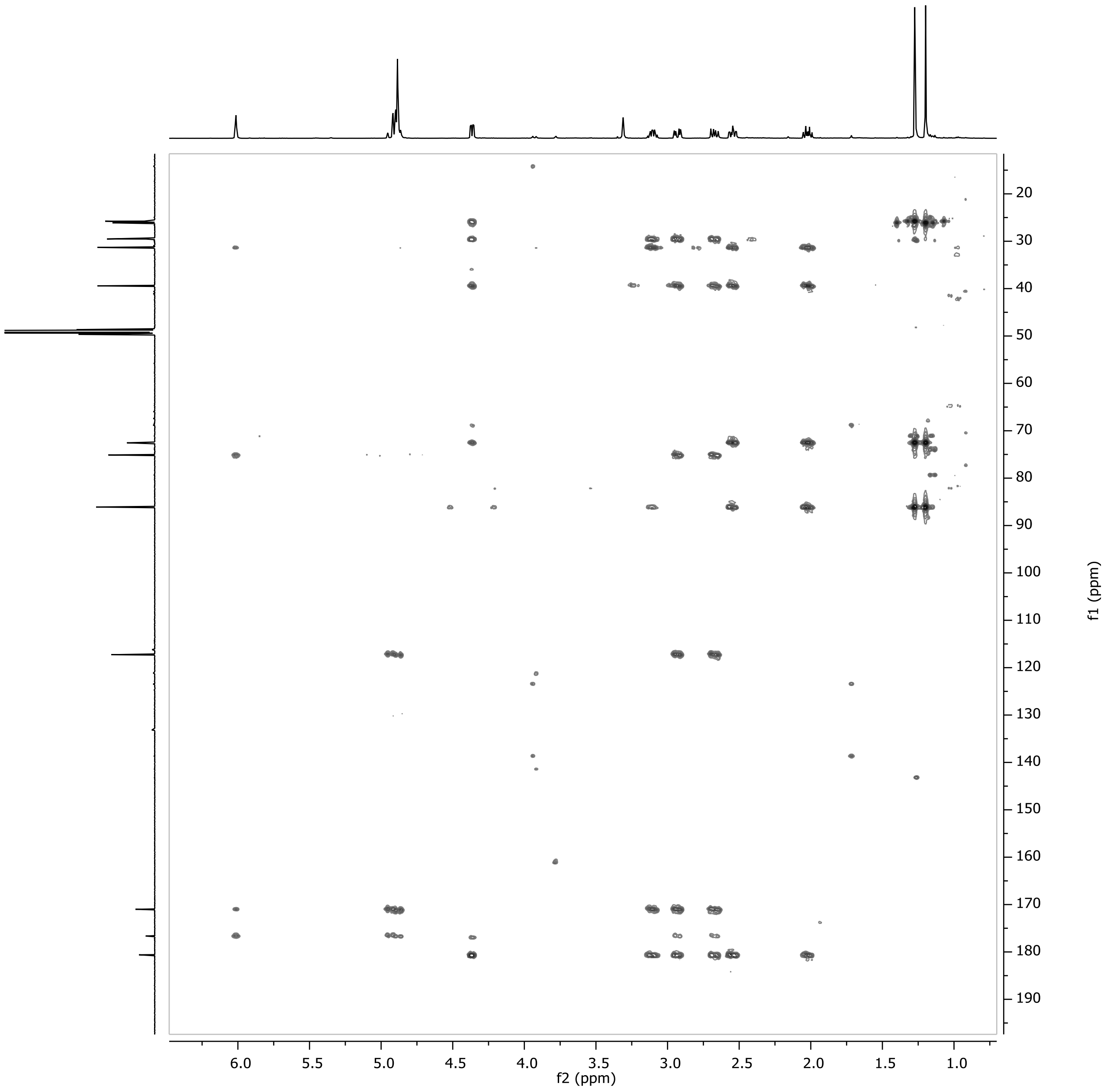

Figure S38. HMBC spectrum of **5** in methanol-d_4 at 500 MHz.

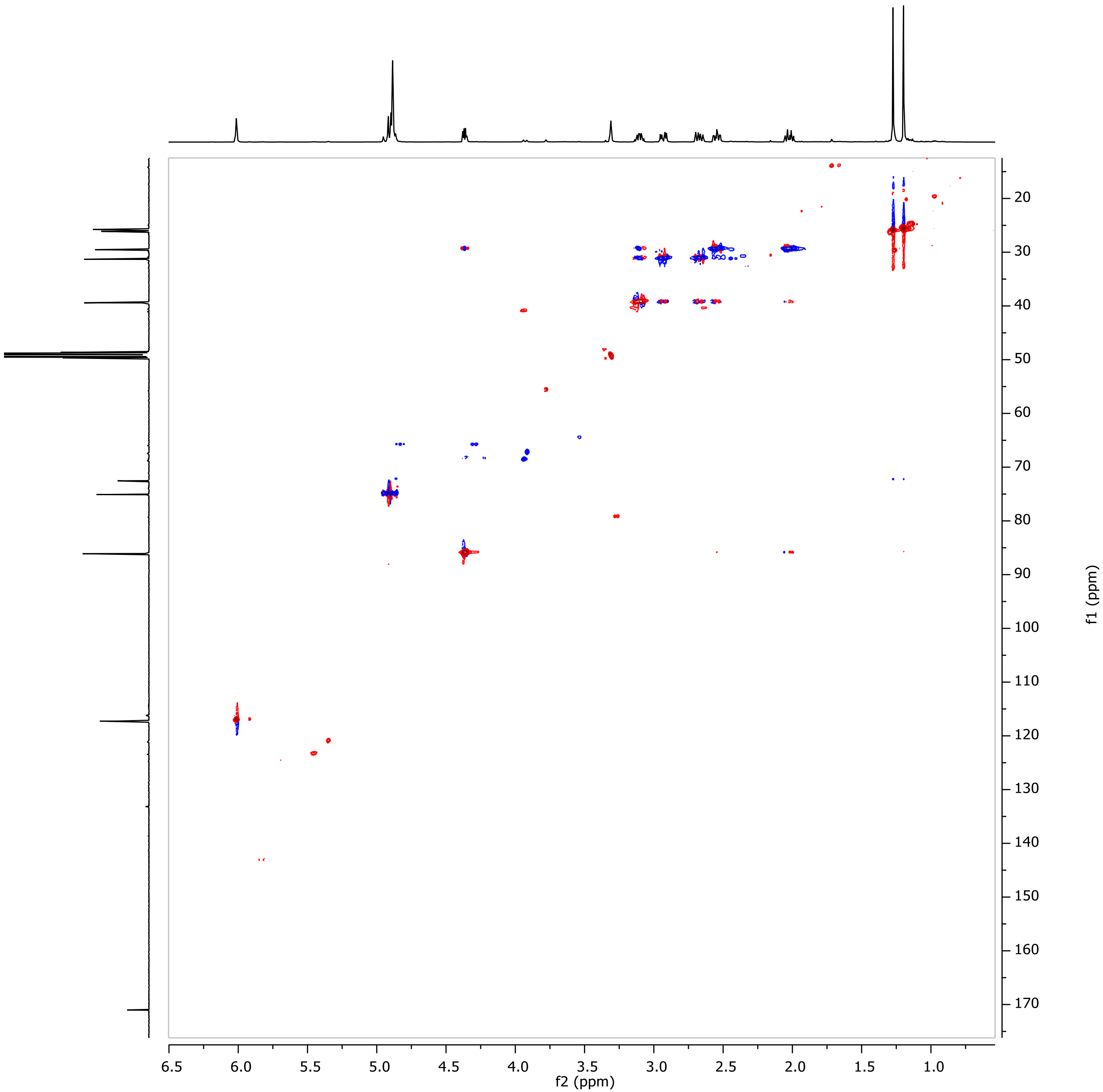

Figure S39. HSQC spectrum of **5** in methanol-d_4 at 500 MHz.

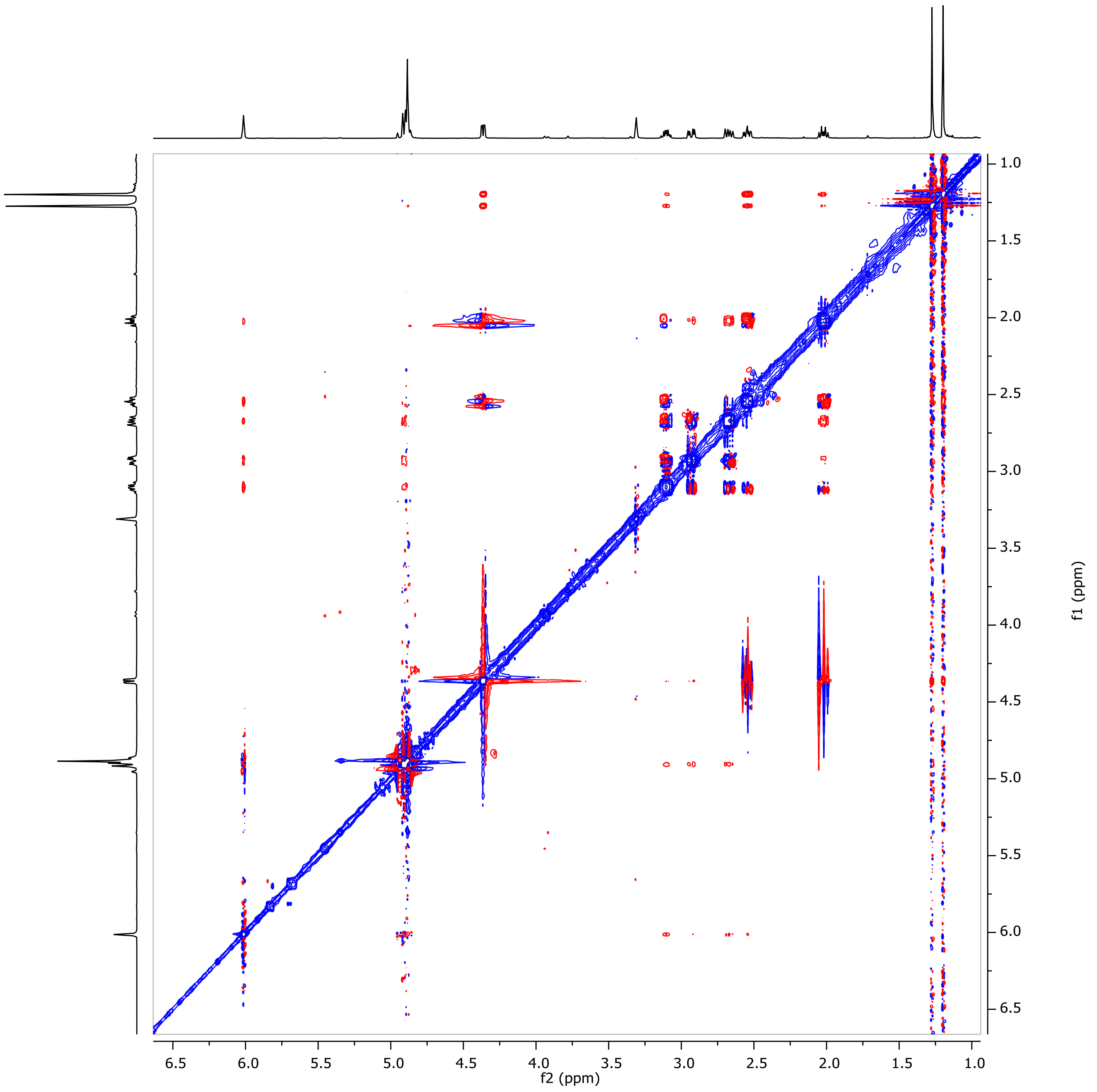

Figure S40. ROESY spectrum of **5** in methanol-d_4 at 500 MHz.

Generic Display Report

Analysis Info

Analysis Name	S:\PEOPLE\sel22_Sherif Elsayed\Bondarzewia\AmaZon\IHI 766R2F14_GE6_01_50348.d	Acquisition Date	02.09.2023 12:07:45
Method	50348.m	Operator	tti
Sample Name	IHI 766R2F14	Instrument	amaZon speed
Comment			

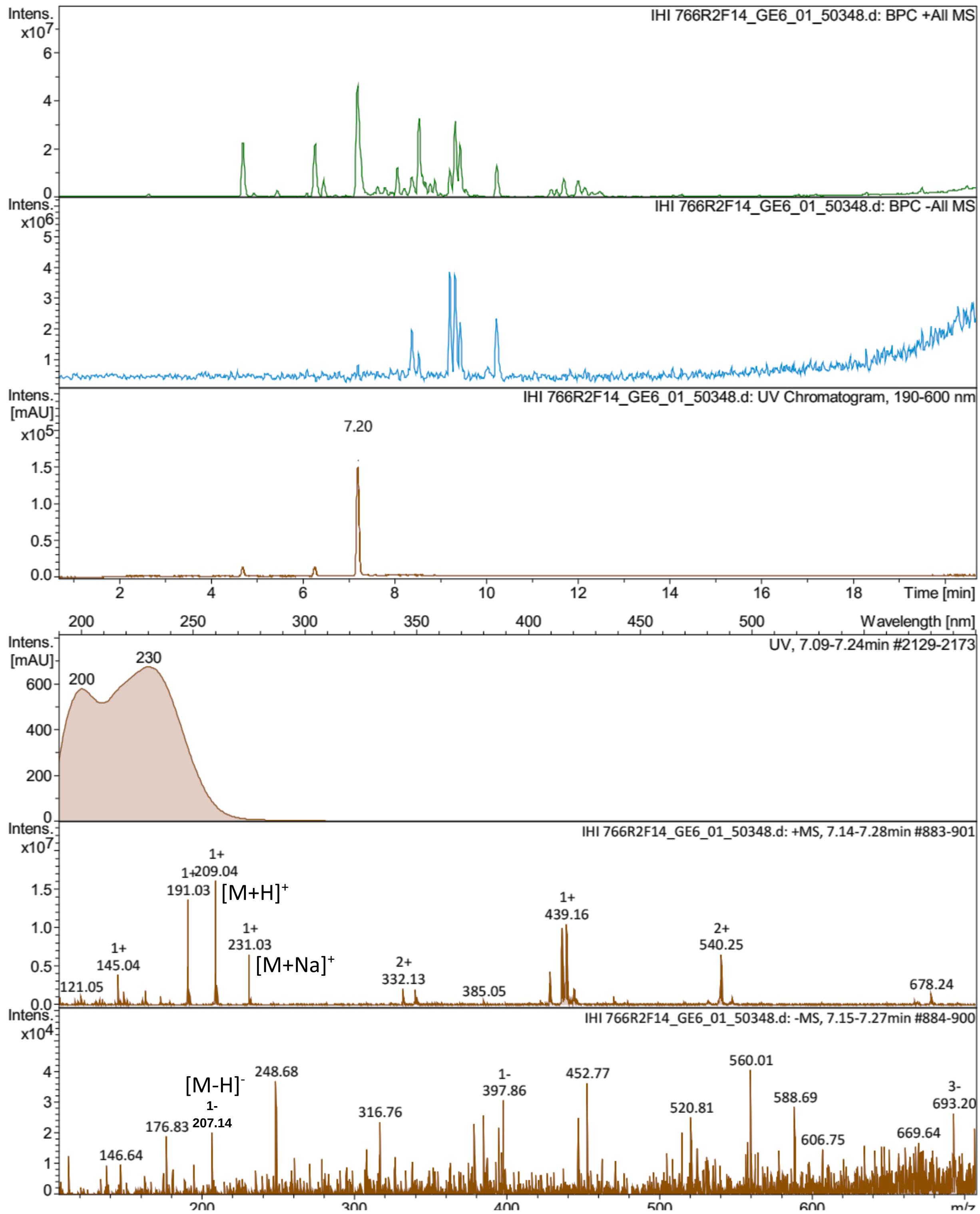

Bruker Compass DataAnalysis 4.4 printed: 11.10.2023 17:54:25 by: sel22 Page 1 of 1

Figure S41. LRESIMS of **6**.

Generic Display Report

Analysis Info

Analysis Name	F:\Volume D\HZI Projects\Winnie\8-Bondarzewia mesenterica\Bondarzewia\MaXis\IHI 766 R2F14_27_01_13123.d	Acquisition Date	05.09.2023 20:22:30
Method	pos_sauce_00_00_screening_ms_100_2500_line.m	Operator	ate06
Sample Name	IHI 766 R2F14	Instrument	maXis
Comment	Screening01<br>Waters Acquity UPLC BEH C_18 1,7um 2.1x50mm		

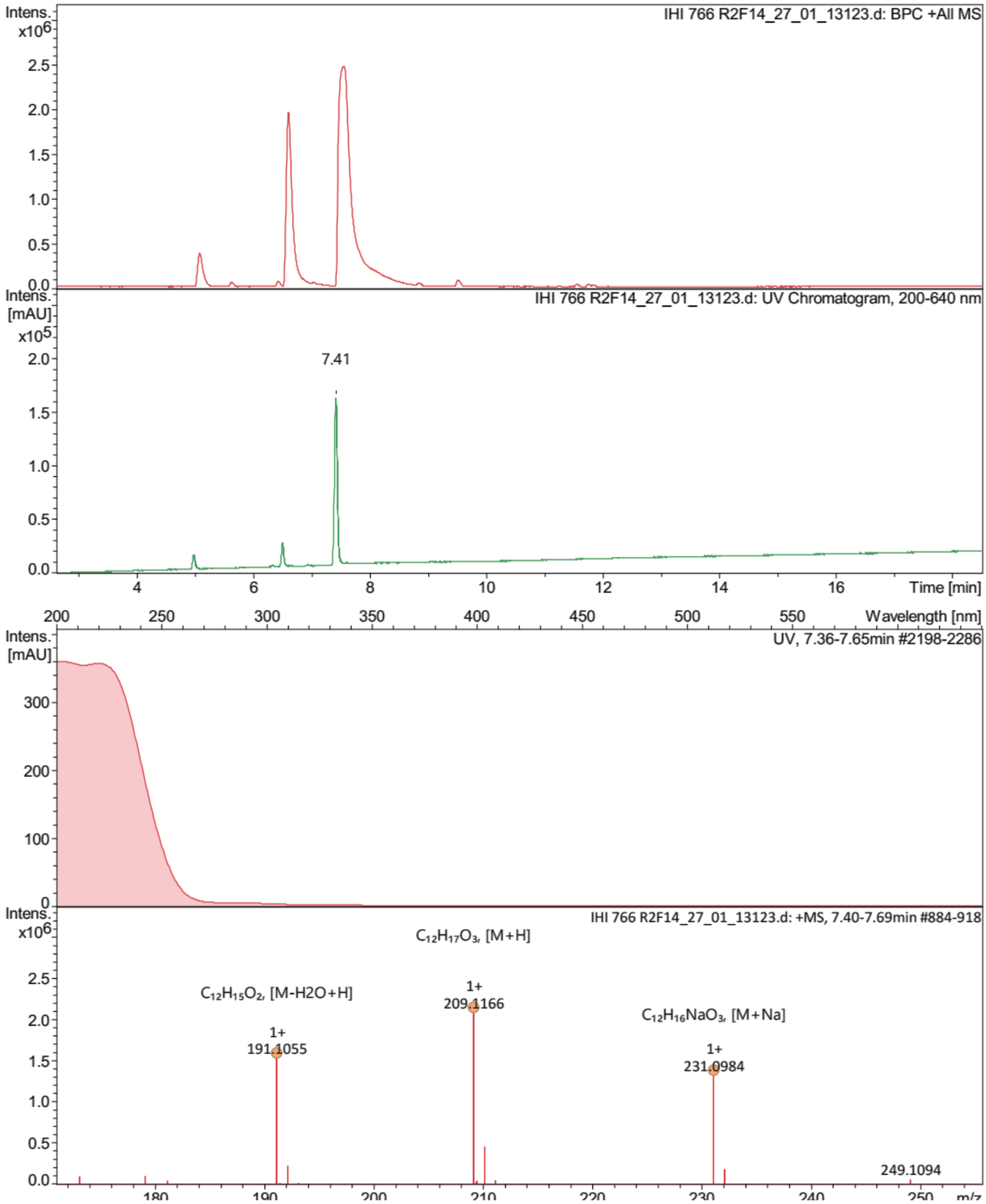

Figure S42. HRESIMS of **6**.

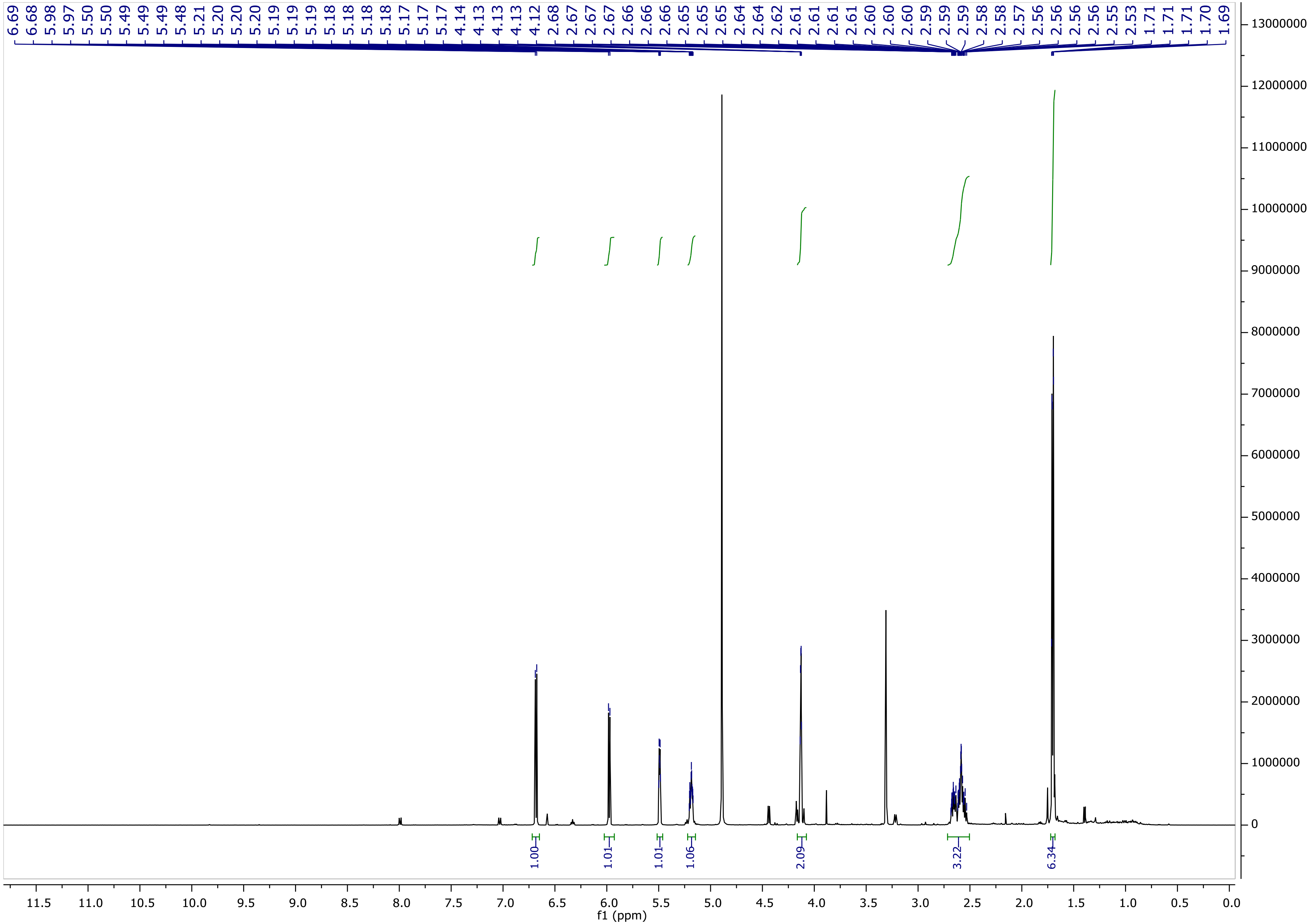

Figure S43. ^{1}H NMR spectrum of **6** in methanol-d_4 at 500 MHz.

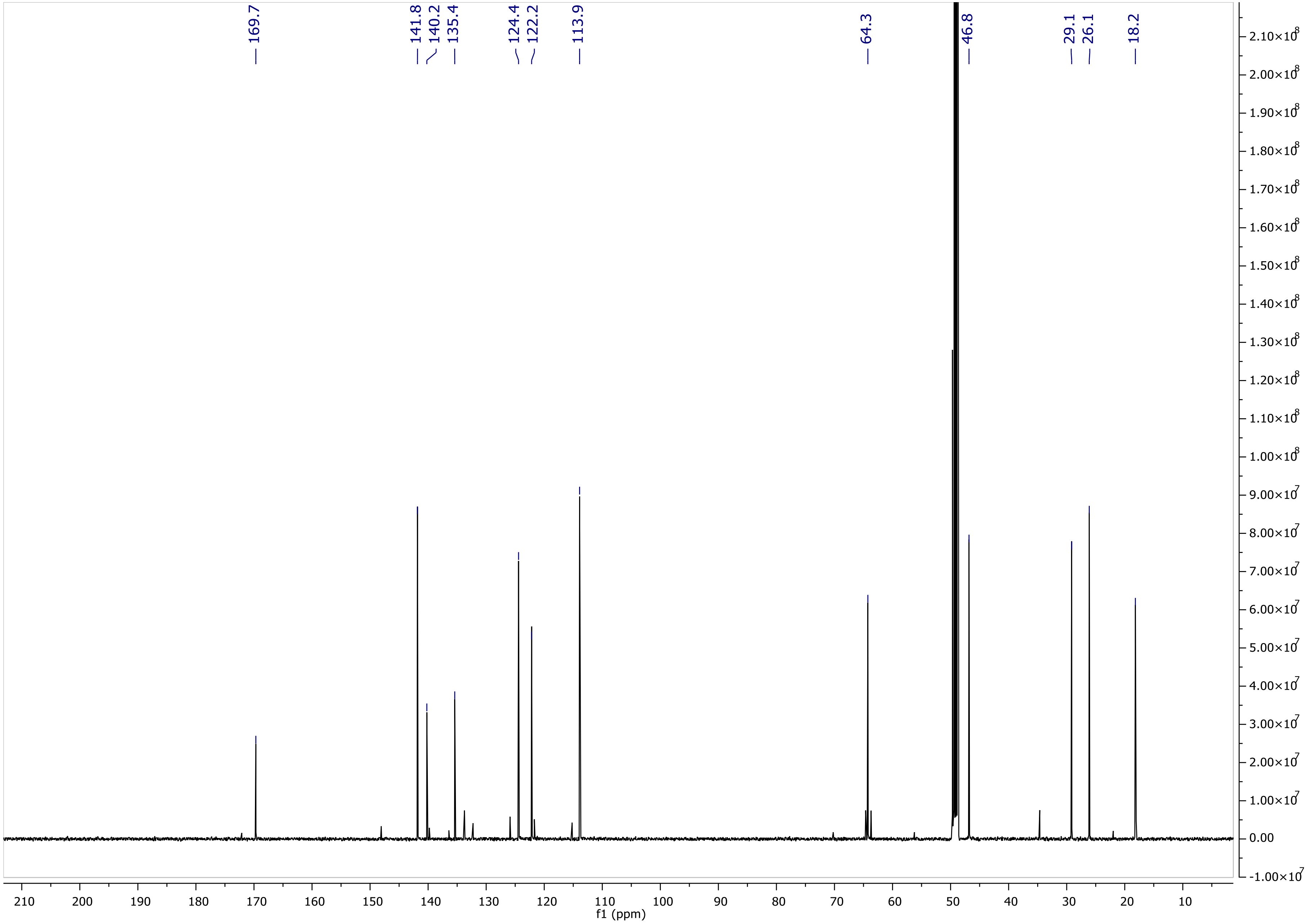

Figure S44. ^{13}C NMR spectrum of **6** in methanol-d_4 at 125 MHz.

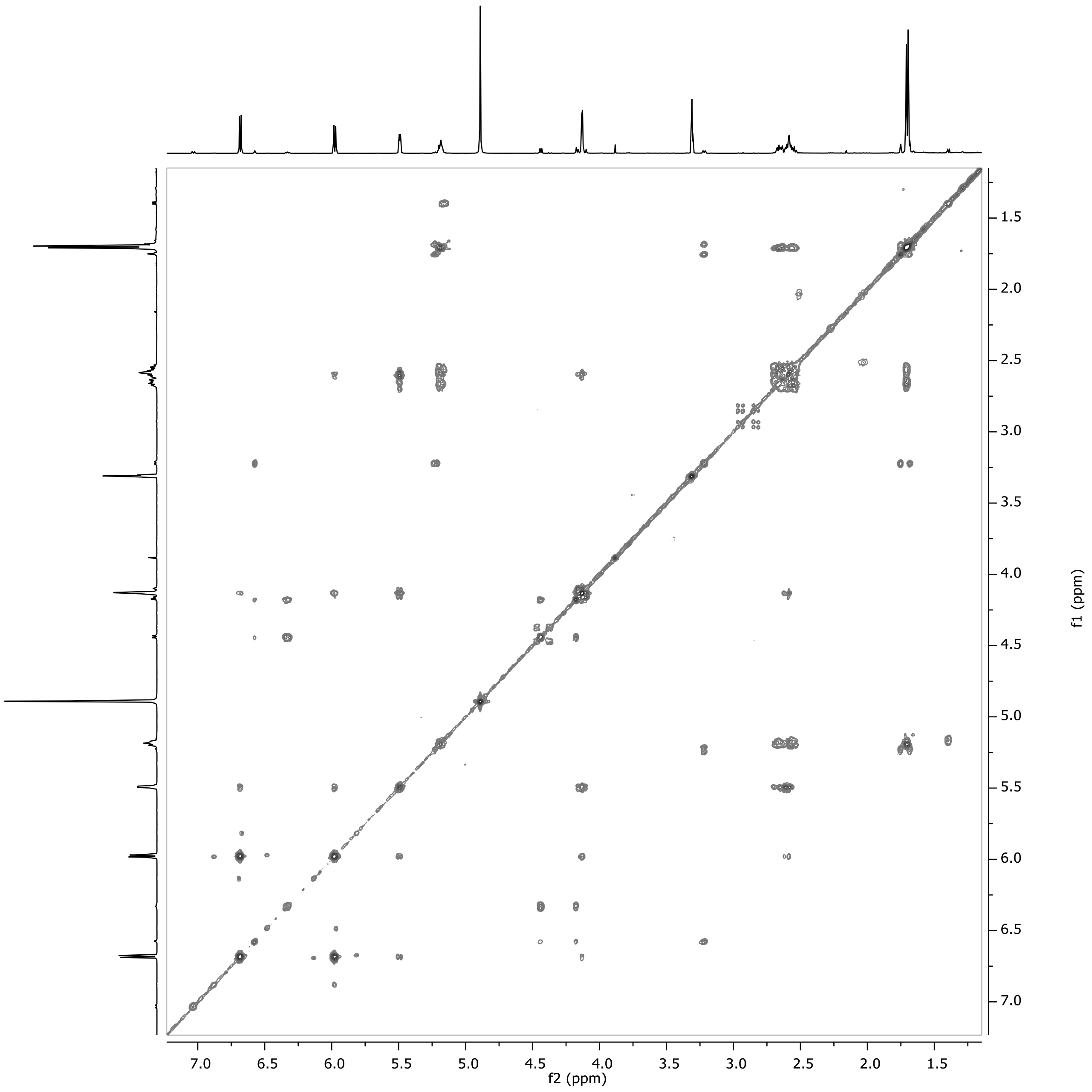

Figure S45. ^{1}H-^{1}H COSY spectrum of **6** in methanol-d_4 at 500 MHz.

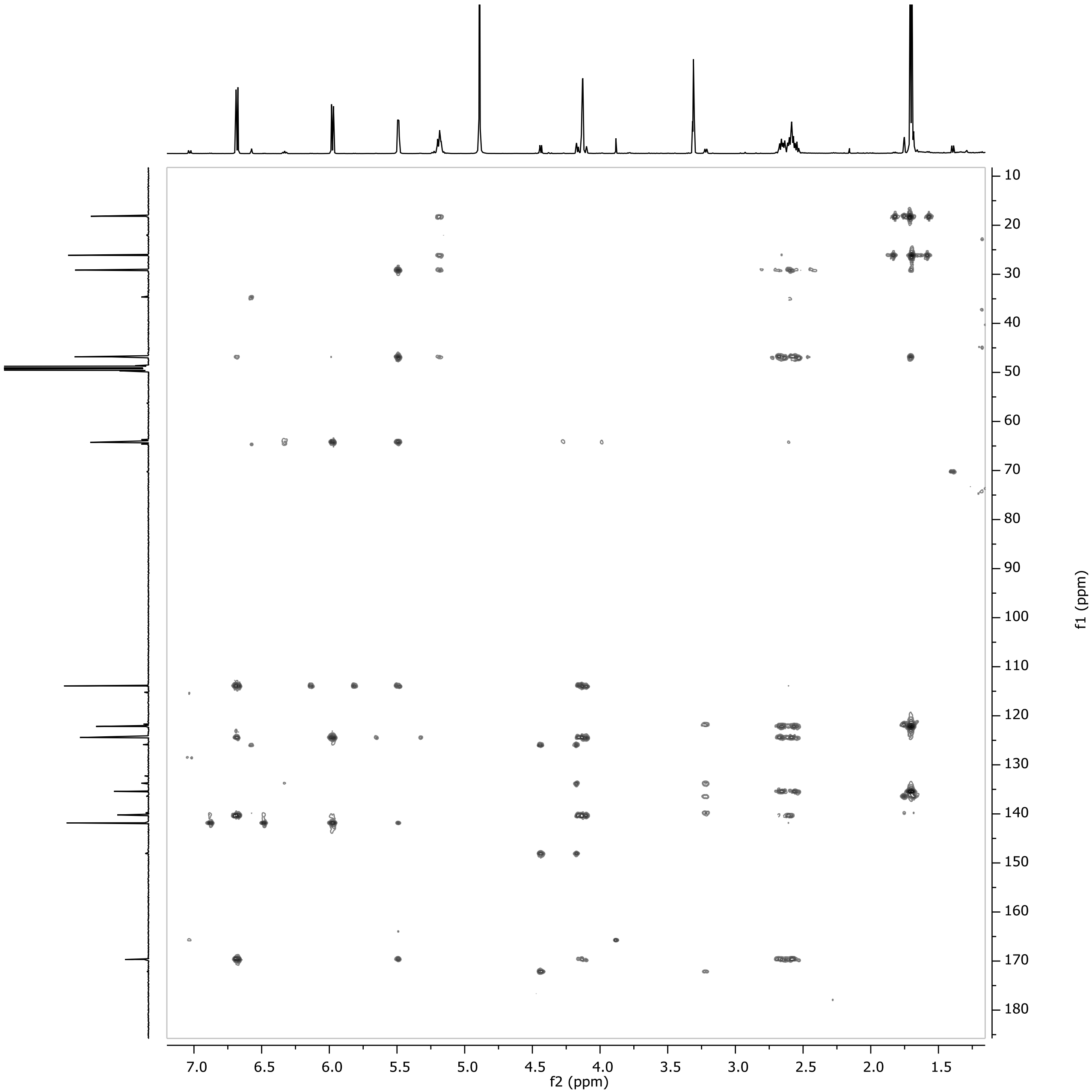

Figure S46. HMBC spectrum of **6** in methanol-d_4 at 500 MHz.

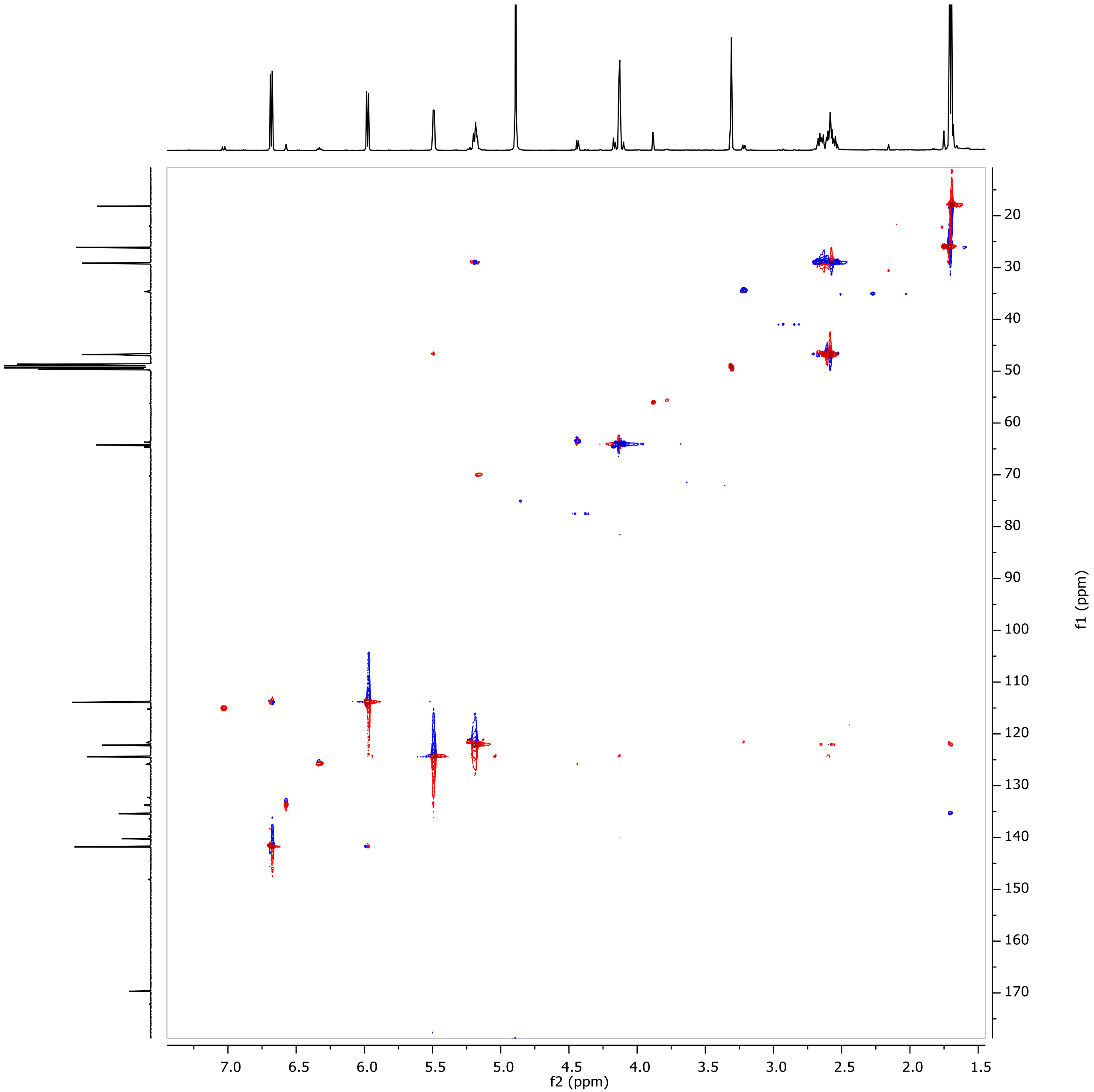

Figure S47. HSQC spectrum of **6** in methanol-d_4 at 500 MHz.

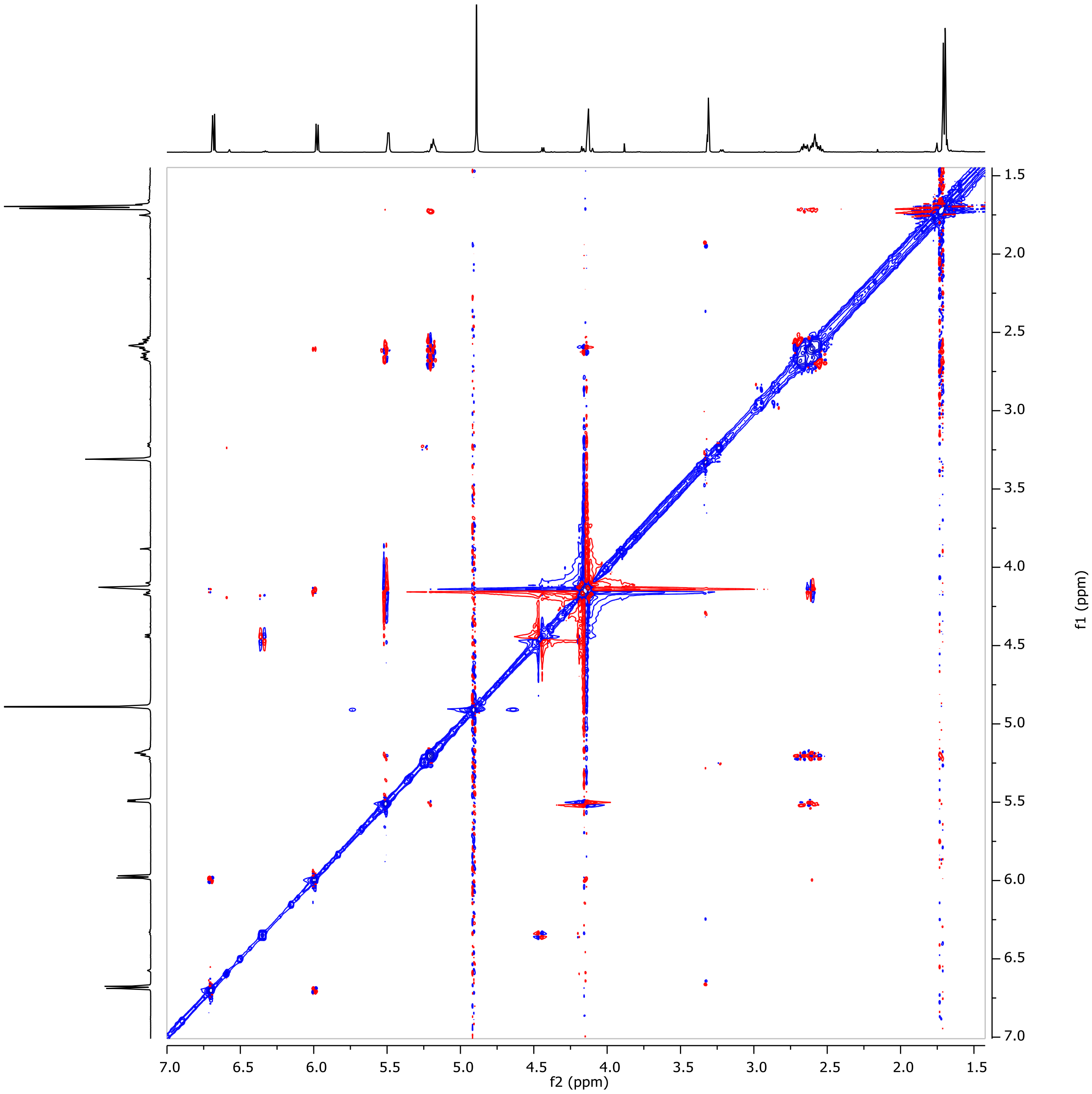

Figure S48. ROESY spectrum of **6** in methanol-d_4 at 500 MHz.

Generic Display Report

Analysis Info

Analysis Name	S:\PEOPLE\sel22_Sherif Elsayed\Bondarzewia\AmaZon\IHI 766R2F16_GE8_01_50352.d	Acquisition Date	02.09.2023 14:32:41
Method	50352.m	Operator	tti
Sample Name	IHI 766R2F16	Instrument	amaZon speed
Comment			

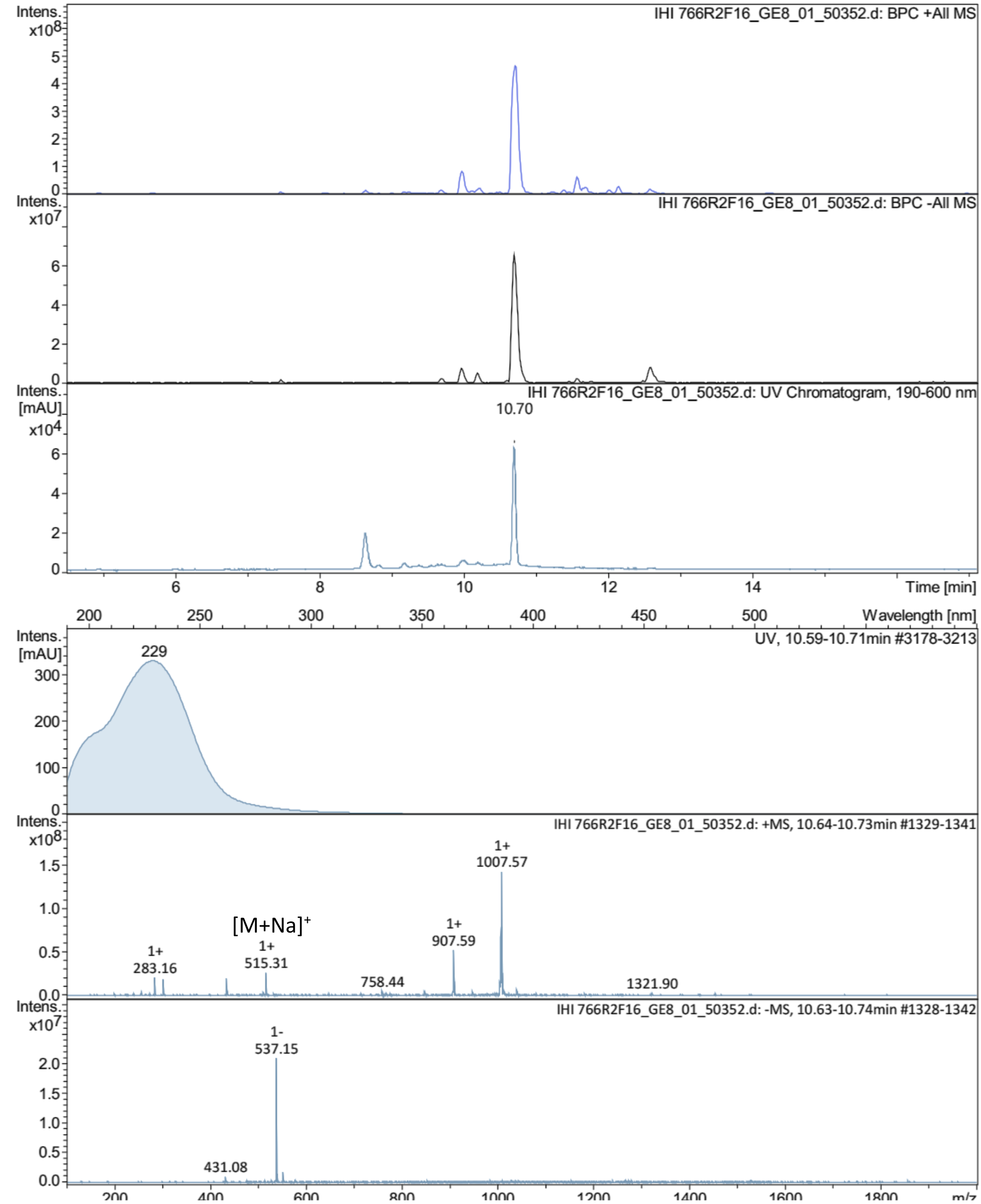

Bruker Compass DataAnalysis 4.4 printed: 11.10.2023 17:57:14 by: sel22 Page 1 of 1

Figure S49. LRESIMS of **7**.

Generic Display Report

Analysis Info

Analysis Name	F:\Volume D\HZI Projects\Winnie\8-Bondarzewia mesenterica\Bondarzewia\MaXis\IHI 766 R2F16_32_01_13128.d	Acquisition Date	05.09.2023 22:57:21
Method	pos_s[illegible]screening_ms_100_2500_line.m	Operator	ate06
Sample Name	IHI 766 R2F16	Instrument	maXis
Comment	Screening01<br>Waters Acquity UPLC BEH C_18 1,7um 2.1x50mm		

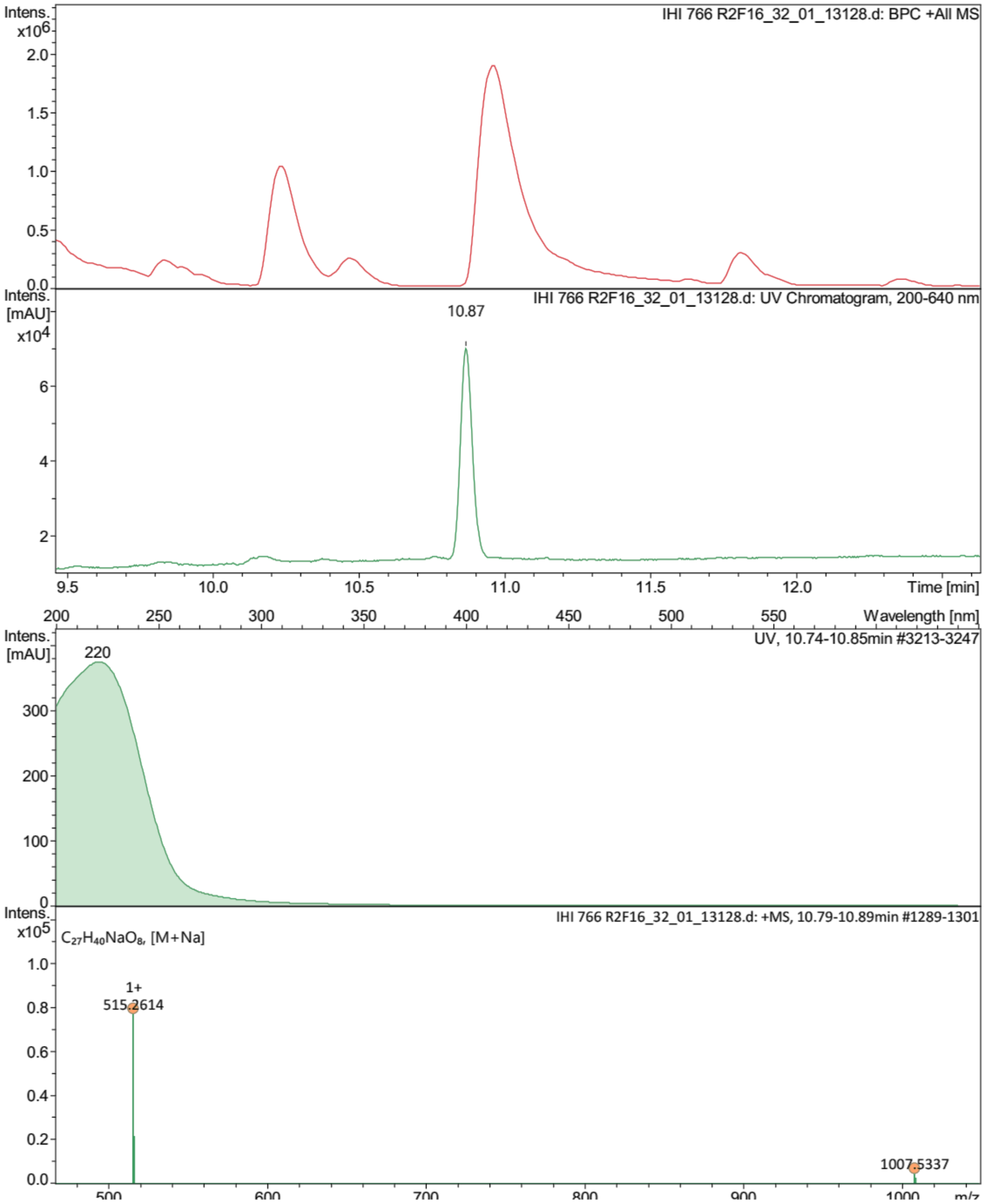

Bruker Compass DataAnalysis 4.4 printed: 16.10.2023 13:41:02 by: sel22 Page 1 of 1

Figure S50. HRESIMS of **7**.

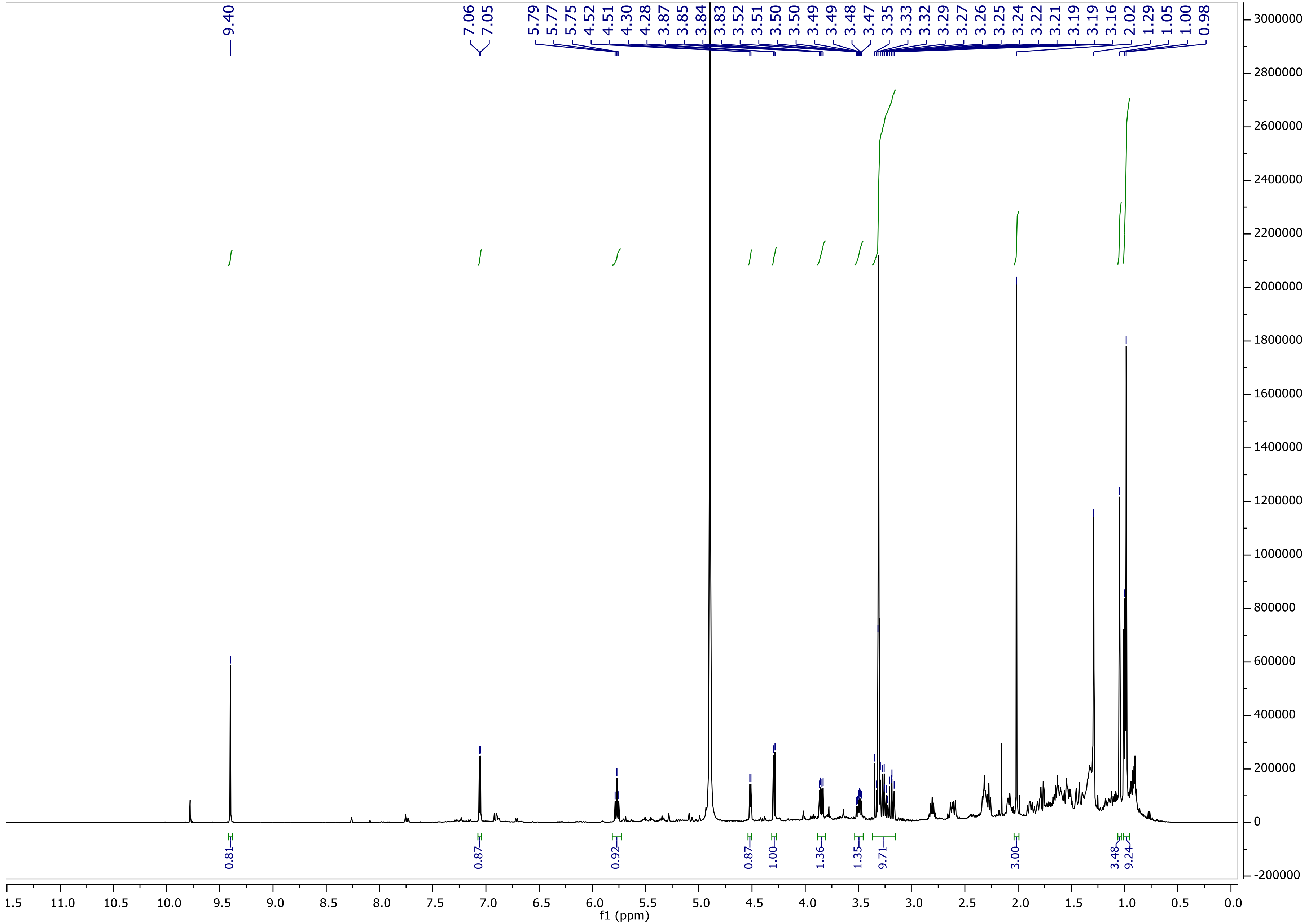

Figure S51. ^{1}H NMR spectrum of **7** in methanol-d_4 at 500 MHz.

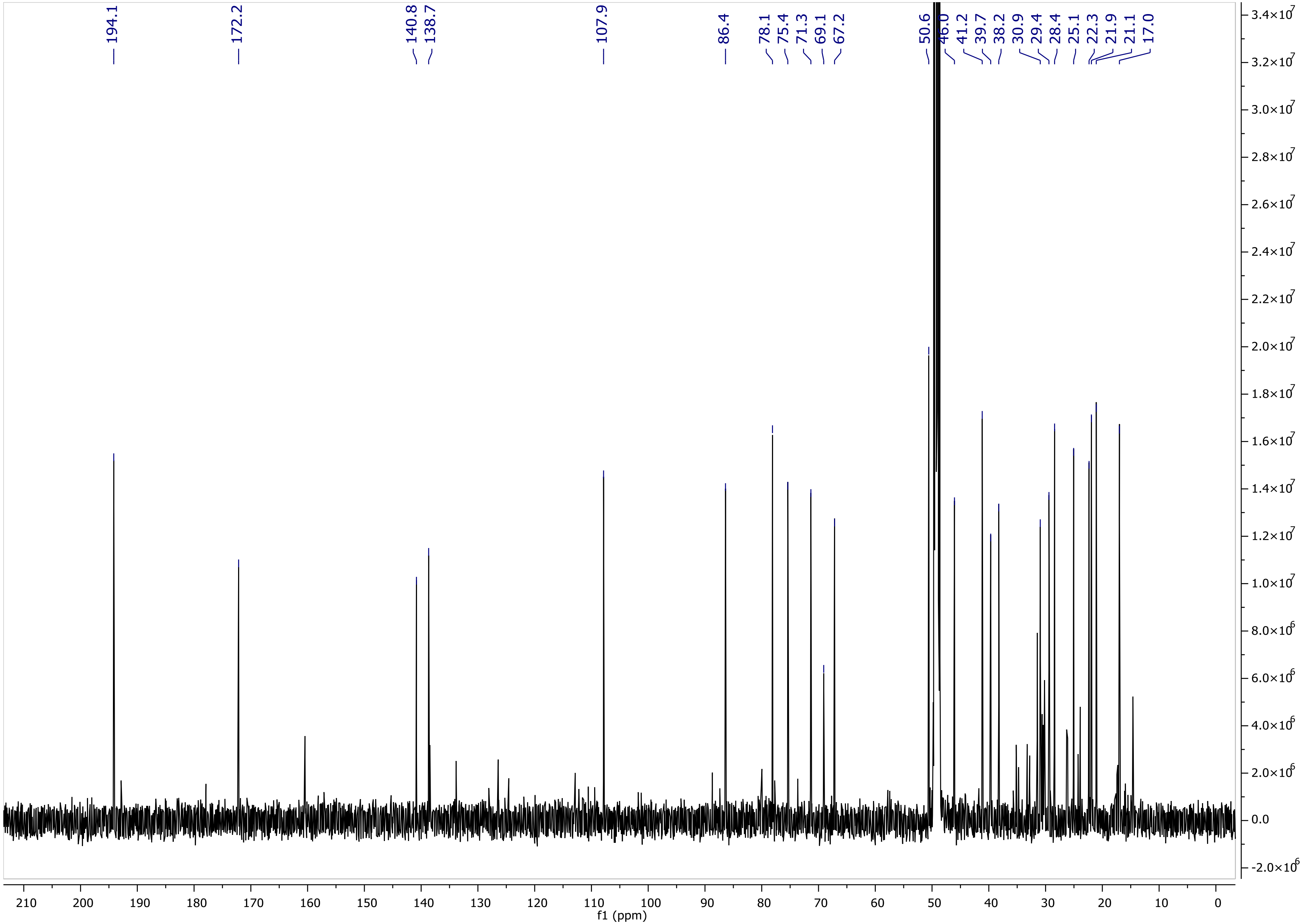

Figure S52. ^{13}C NMR spectrum of **7** in methanol-d_4 at 125 MHz.

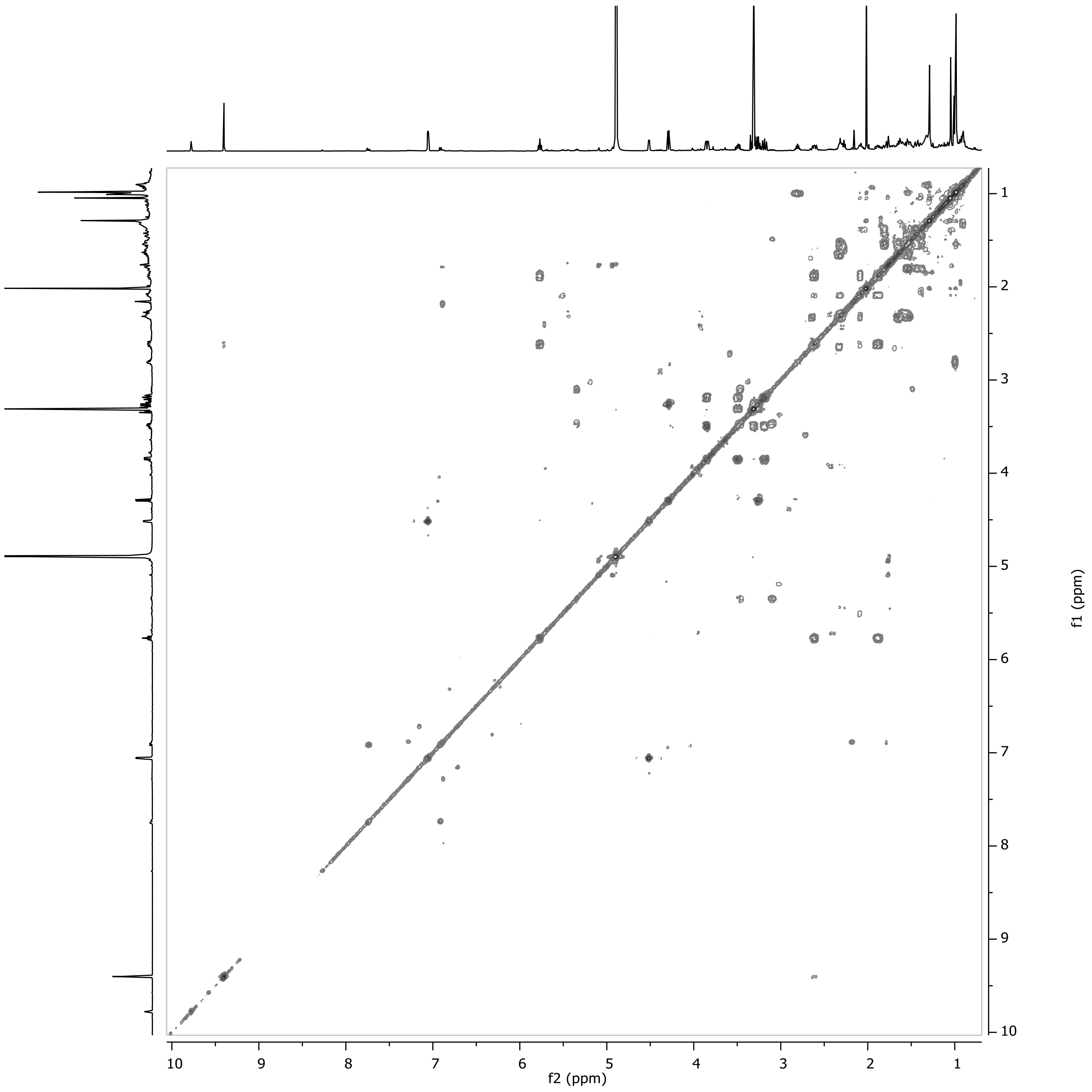

Figure S53. ^{1}H-^{1}H COSY spectrum of **7** in methanol-d_4 at 500 MHz.

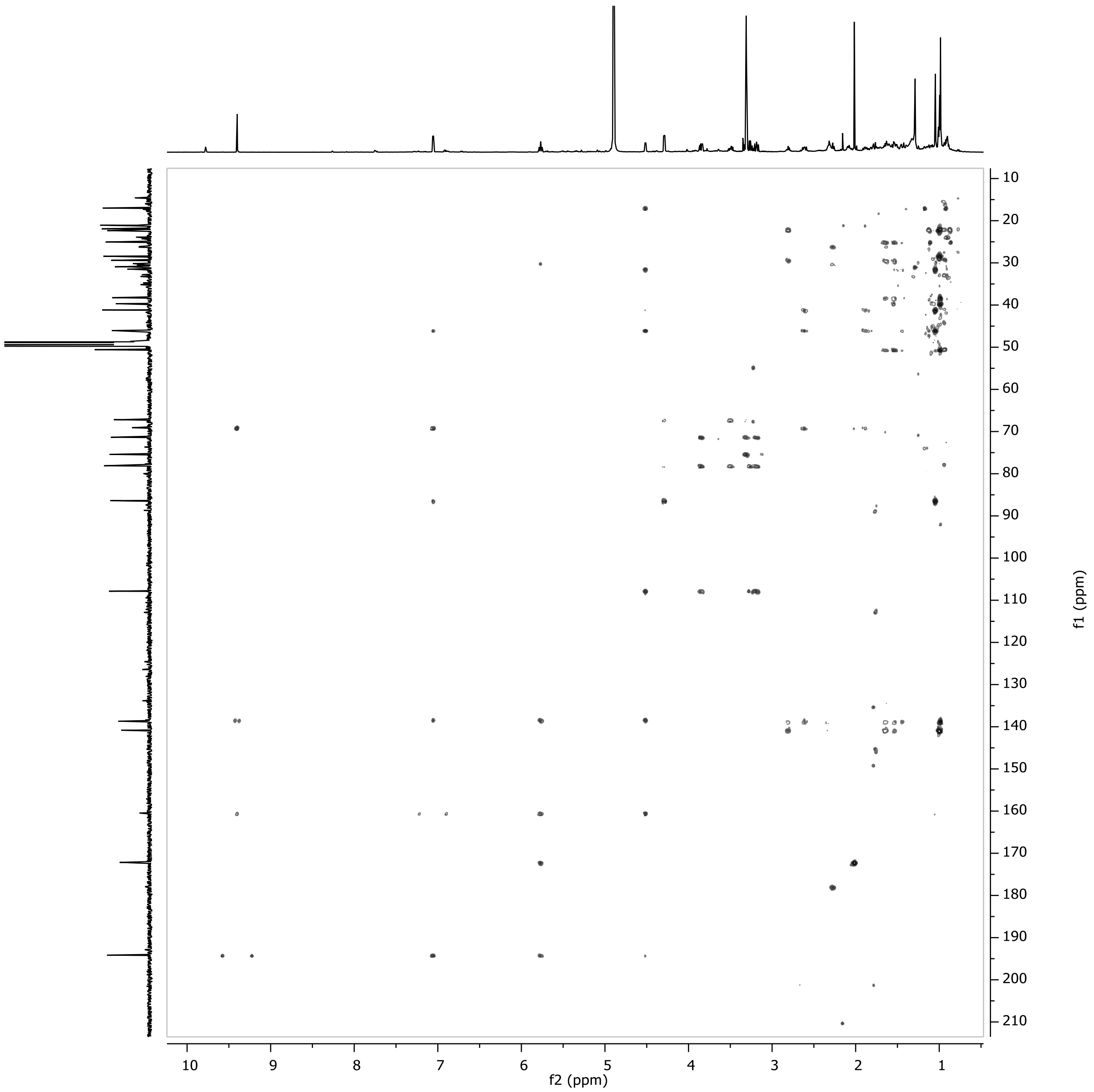

Figure S54. HMBC spectrum of **7** in methanol-d_4 at 500 MHz.

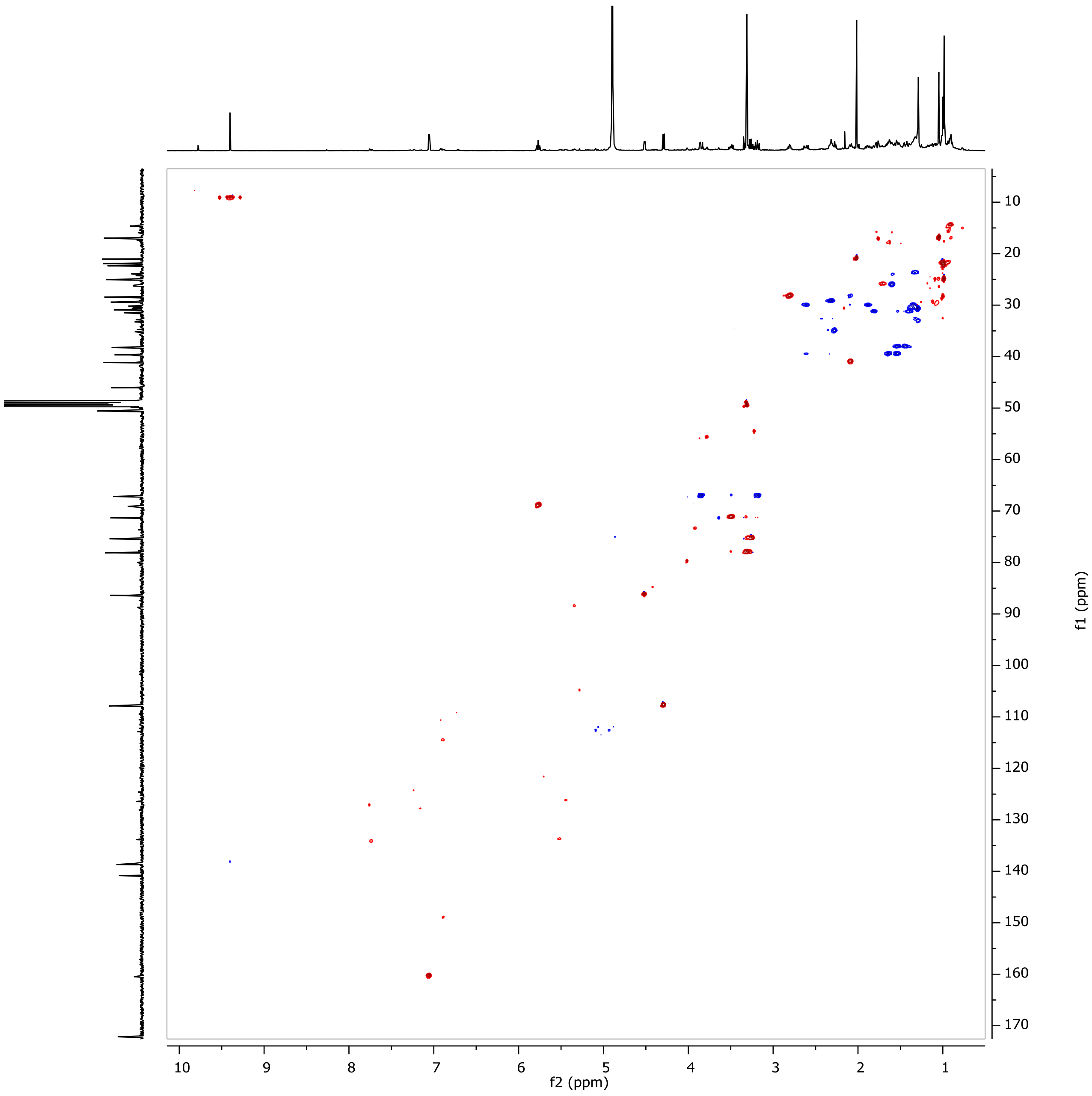

Figure S55. HSQC spectrum of **7** in methanol-d_4 at 500 MHz.

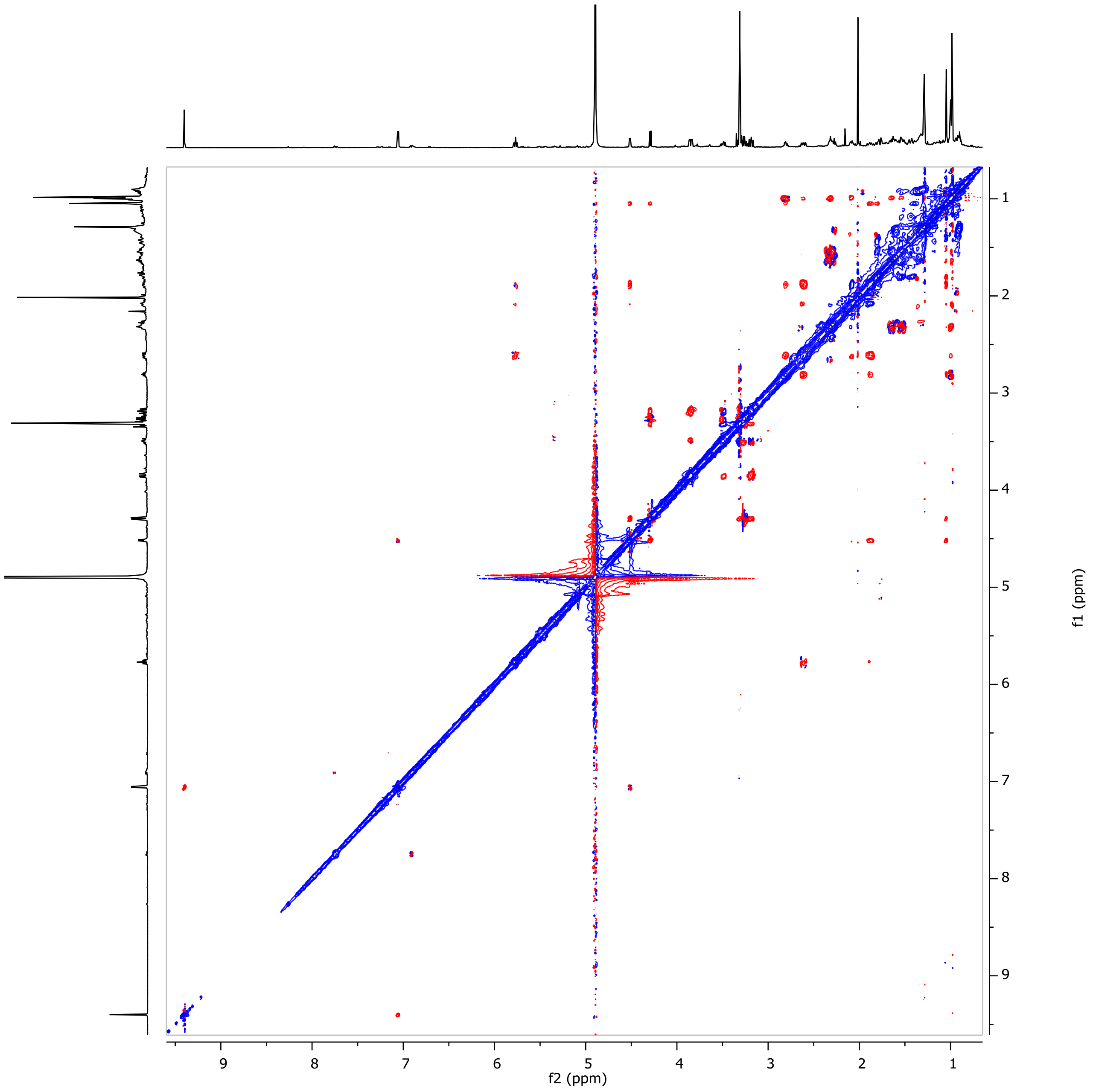

Figure S56. ROESY spectrum of **7** in methanol-d_4 at 500 MHz.

Generic Display Report

Analysis Info

Analysis Name	S:\PEOPLE\sel22_Sherif Elsayed\Bondarzewia\AmaZon\IHI 766R2F17_GA4_01_50353.d	Acquisition Date	02.09.2023 15:09:03
Method	50353.m	Operator	tti
Sample Name	IHI 766R2F17	Instrument	amaZon speed
Comment			

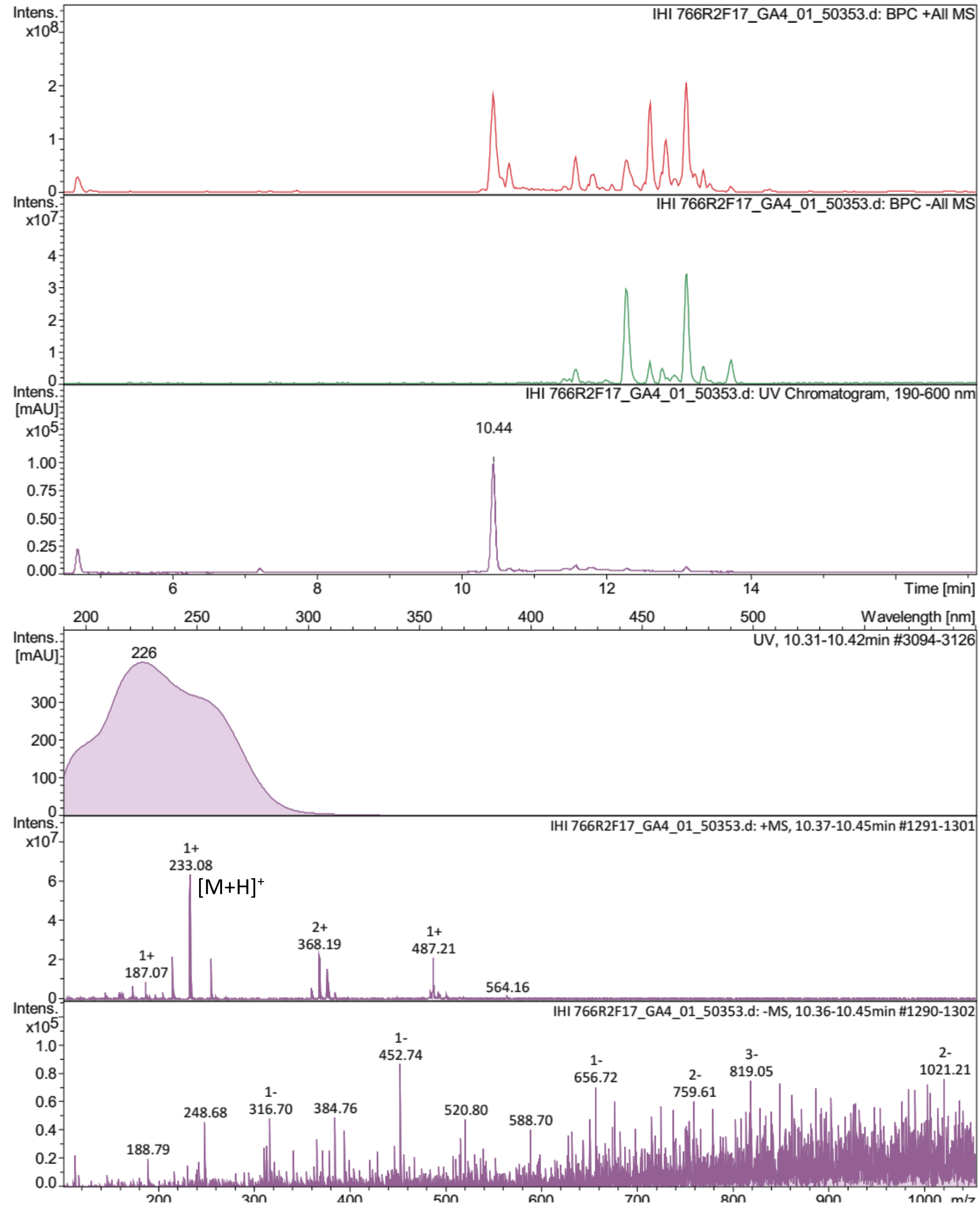

Bruker Compass DataAnalysis 4.4   printed: 11.10.2023 17:57:59   by: sel22   Page 1 of 1

Figure S57. LRESIMS of **8**.

Generic Display Report

Analysis Info

Analysis Name	F:\Volume D\HZI Projects\Winnie\8-Bondarzewia mesenterica\Bondarzewia\MaXis\IHI 766 R2F17_29_01_13125.d	Acquisition Date	05.09.2023 21:24:26
Method	pos_[illegible]_screening_ms_100_2500_line.m	Operator	ate06
Sample Name	IHI 766 R2F17	Instrument	maXis
Comment	Screening01<br>Waters Acquity UPLC BEH C_18 1,7um 2.1x50mm		

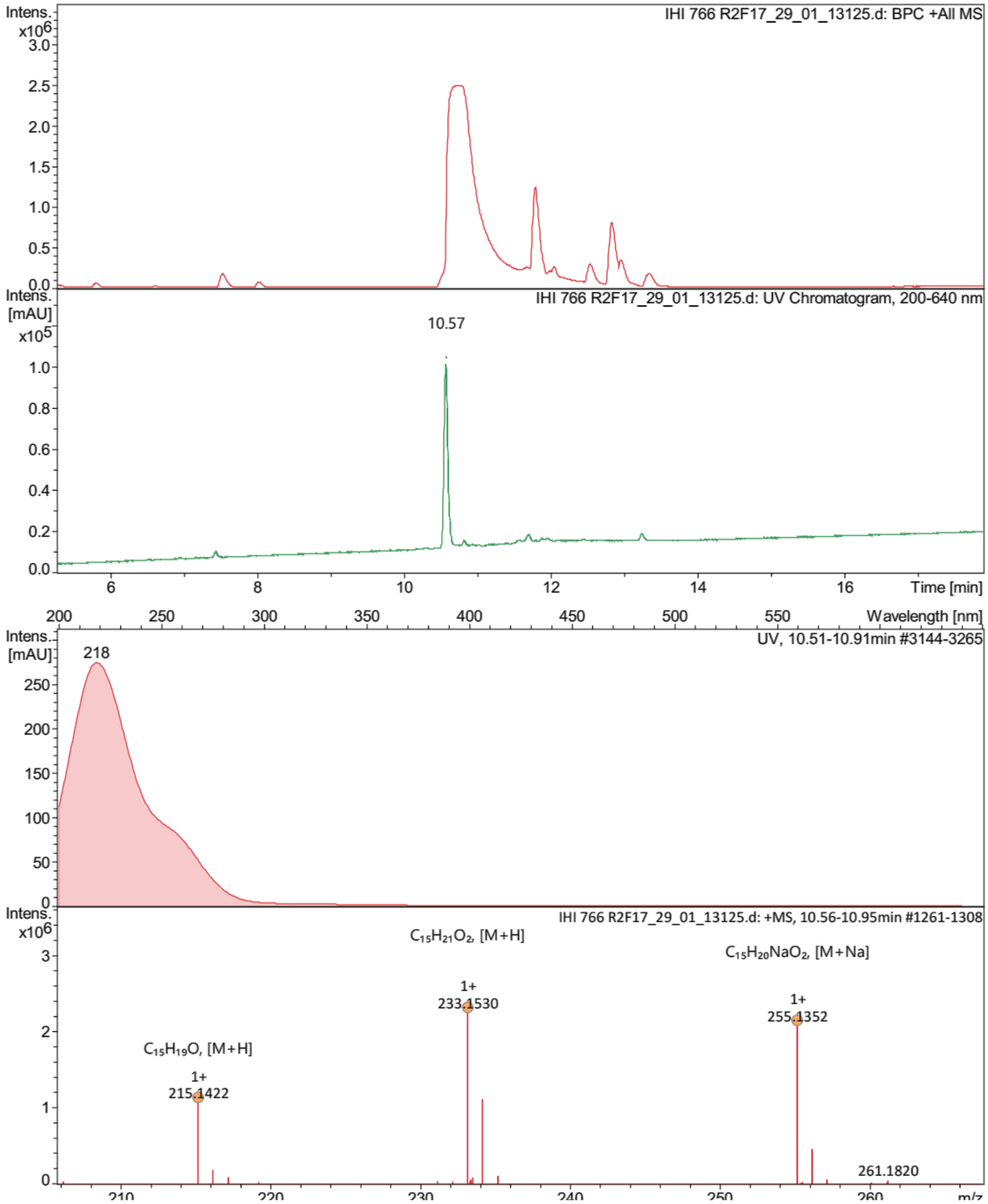

Figure S58. HRESIMS of **8**.

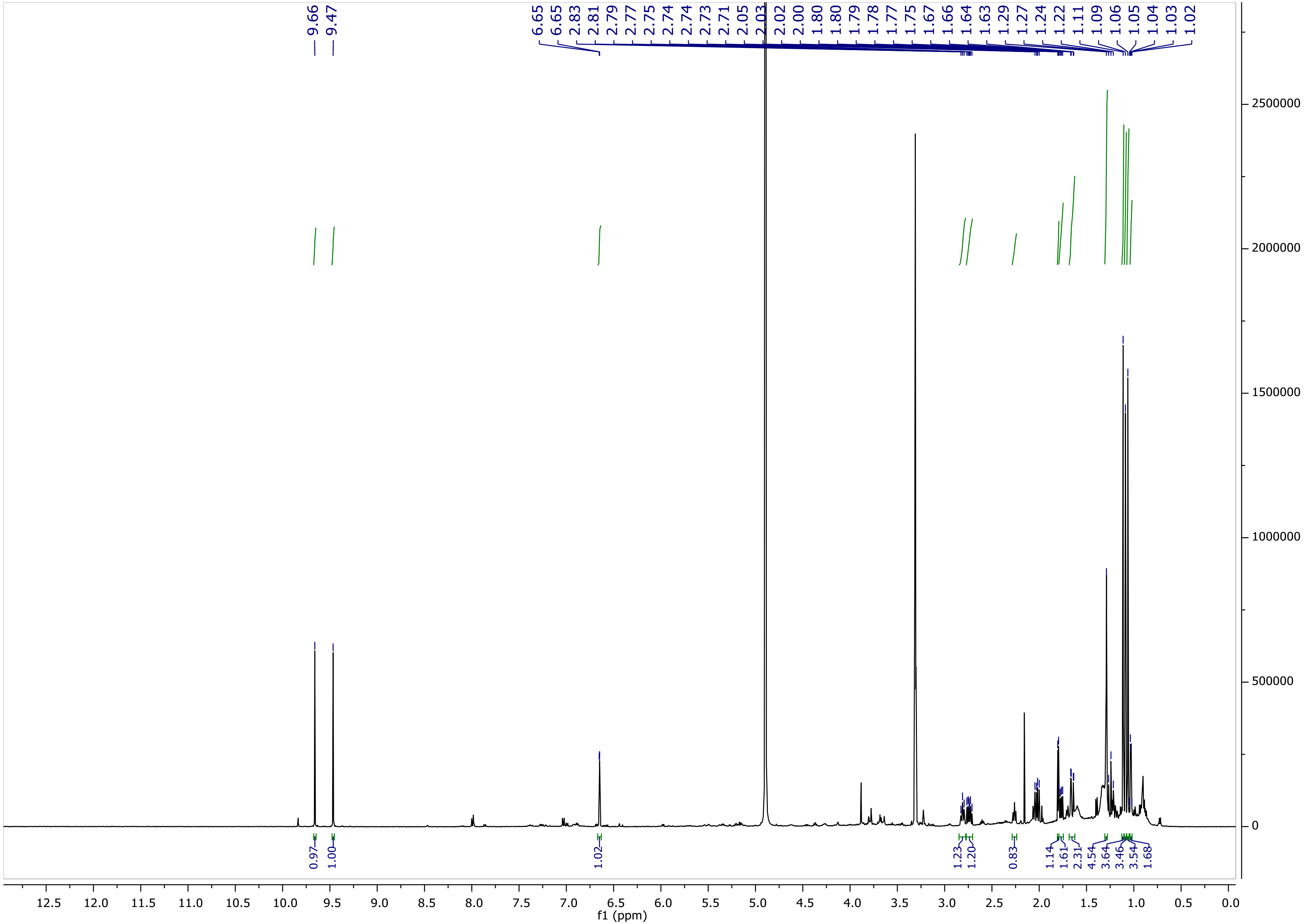

Figure S59. ^{1}H NMR spectrum of **8** in methanol-d_4 at 500 MHz.

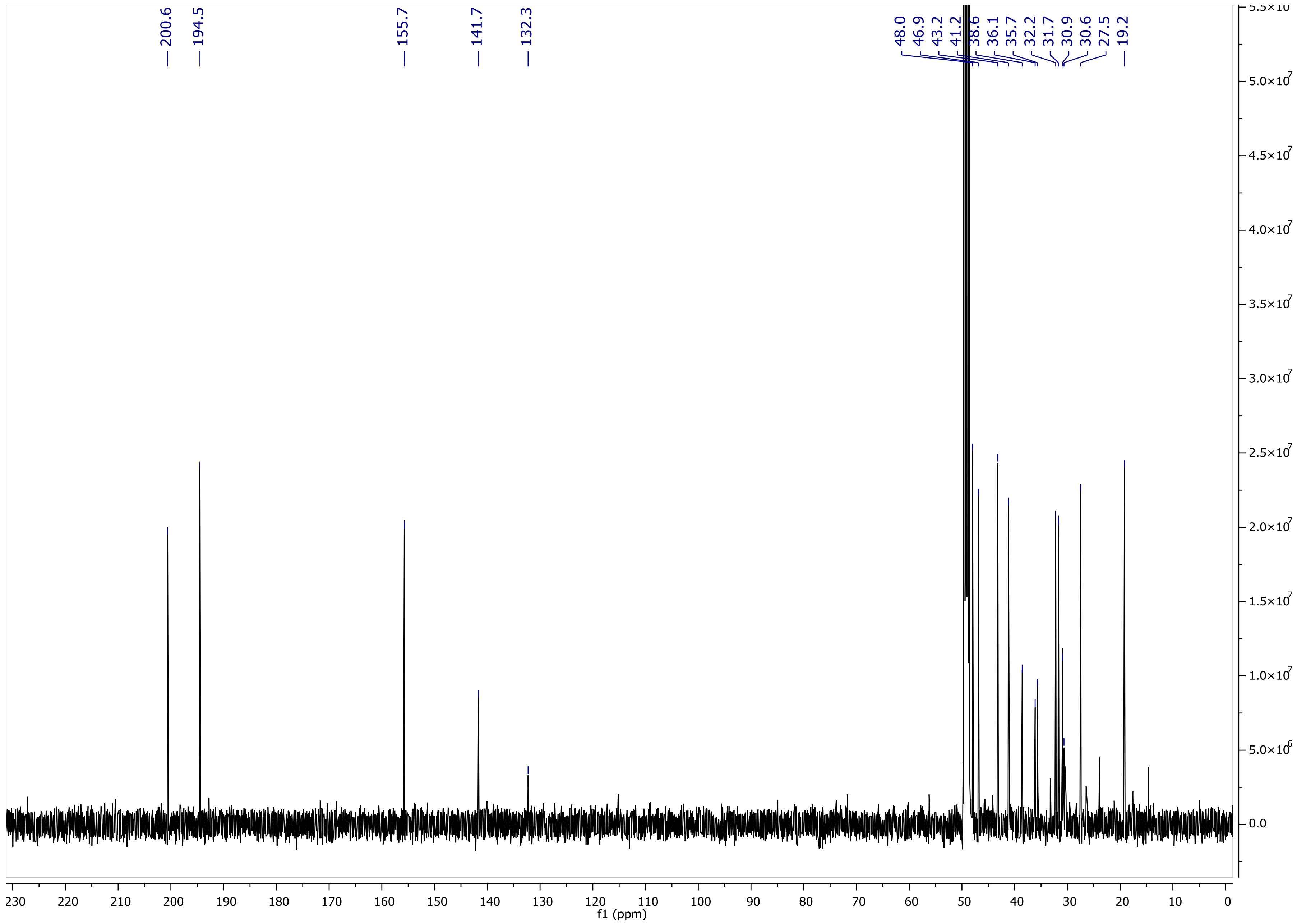

Figure S60. ^{13}C NMR spectrum of **8** in methanol-*d₄* at 125 MHz.

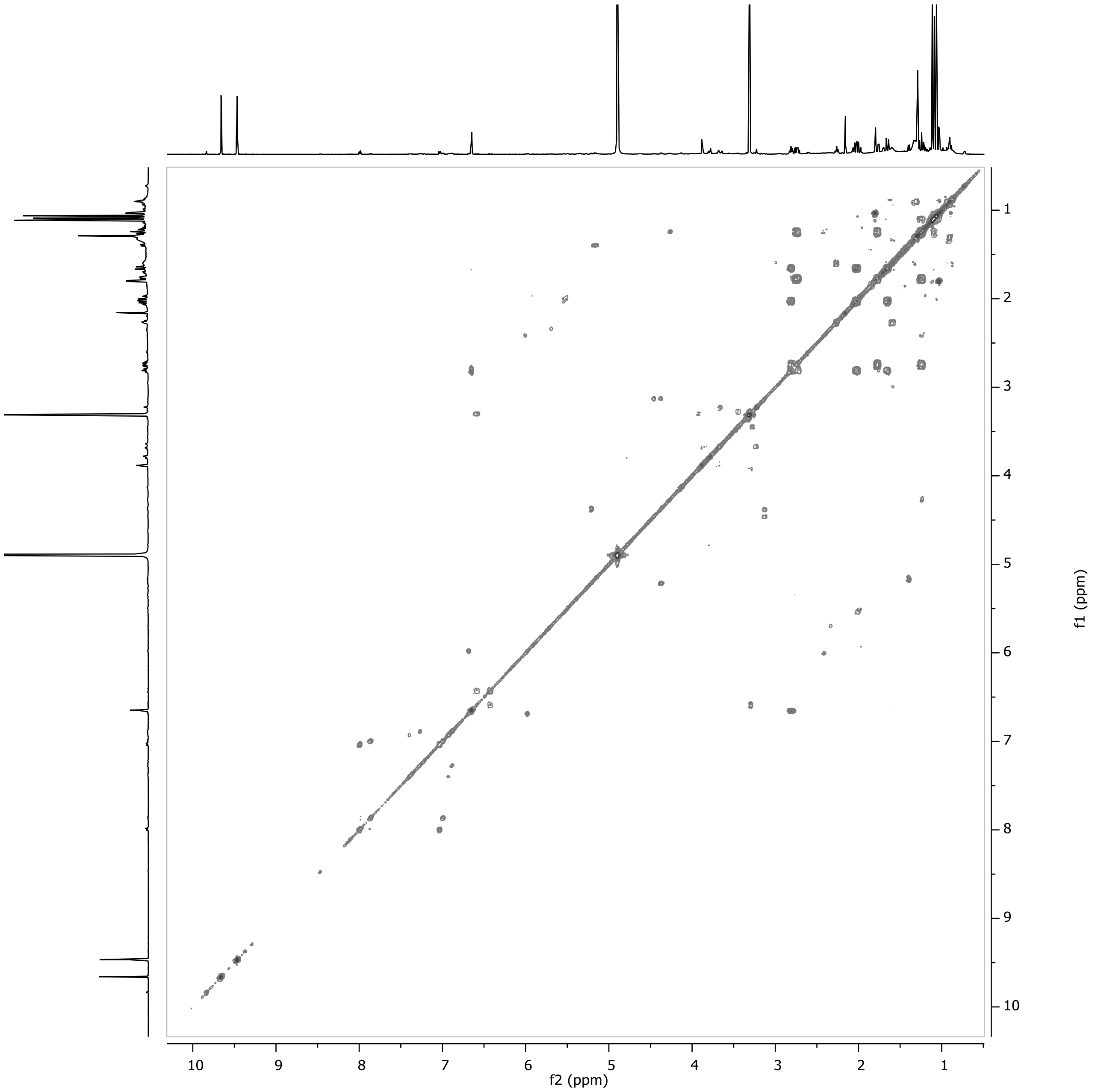

Figure S61. ^{1}H-^{1}H COSY spectrum of **8** in methanol-d_4 at 500 MHz.

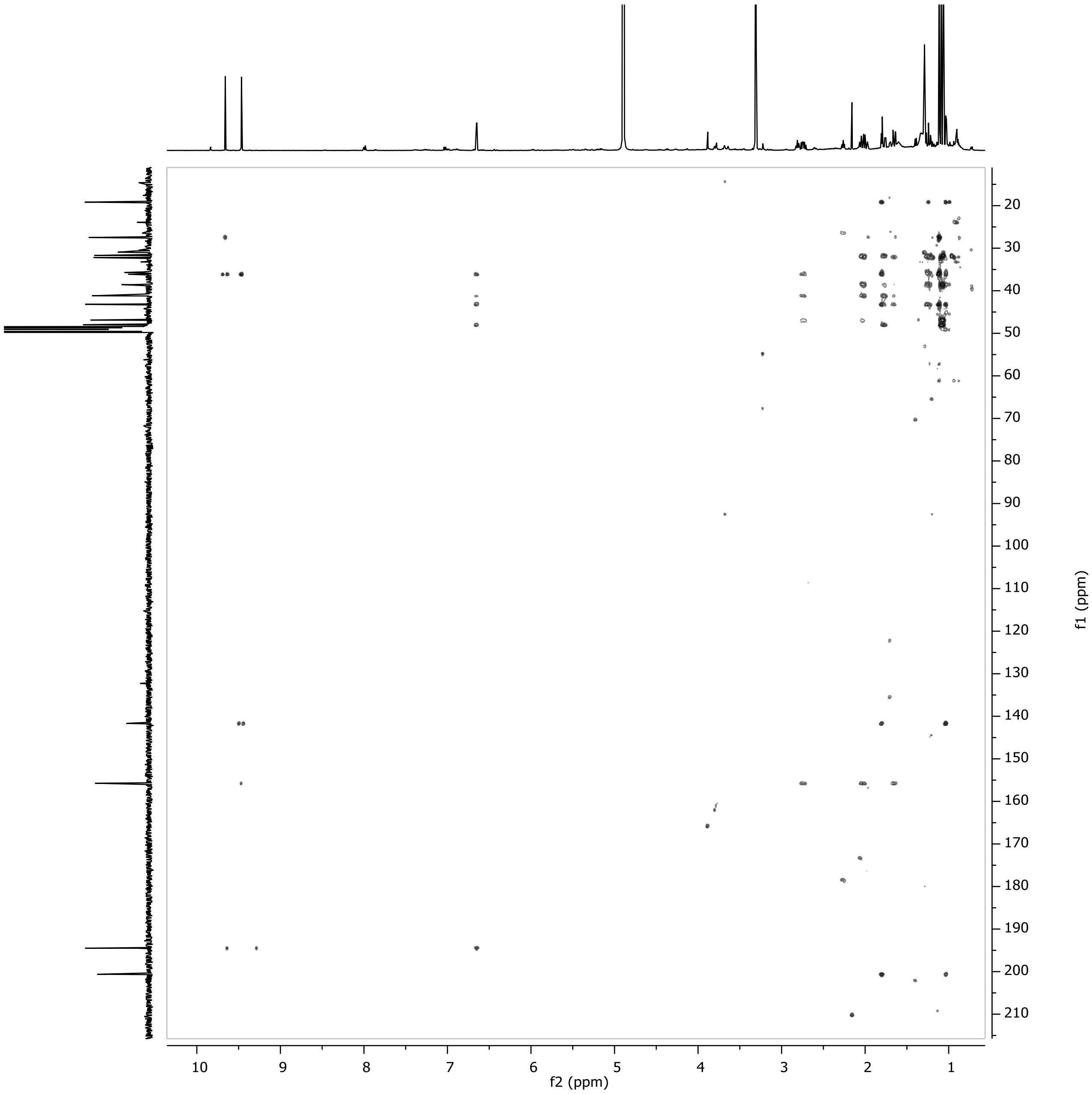

Figure S62. HMBC spectrum of **8** in methanol-d_4 at 500 MHz.

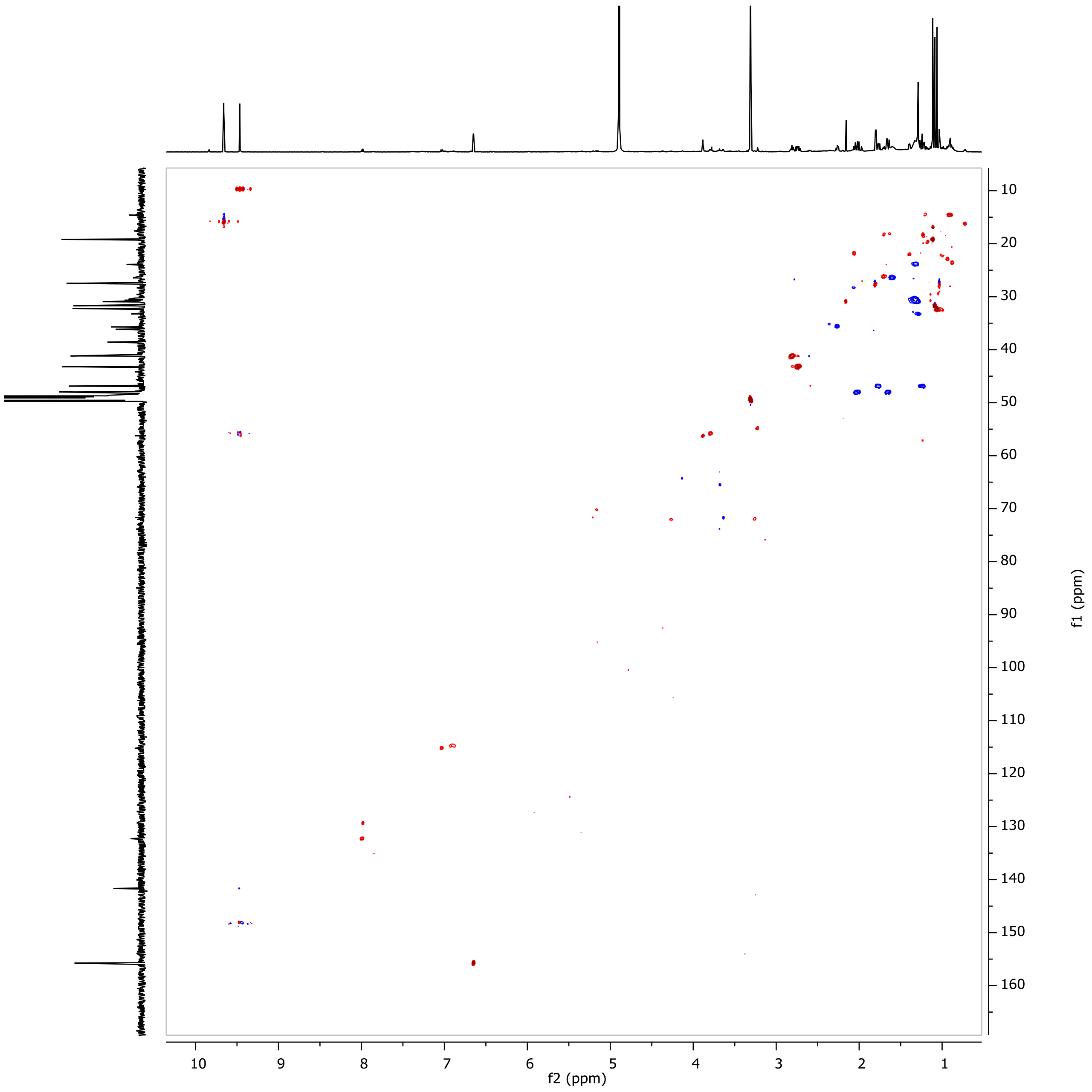

Figure S63. HSQC spectrum of **8** in methanol-d_4 at 500 MHz.

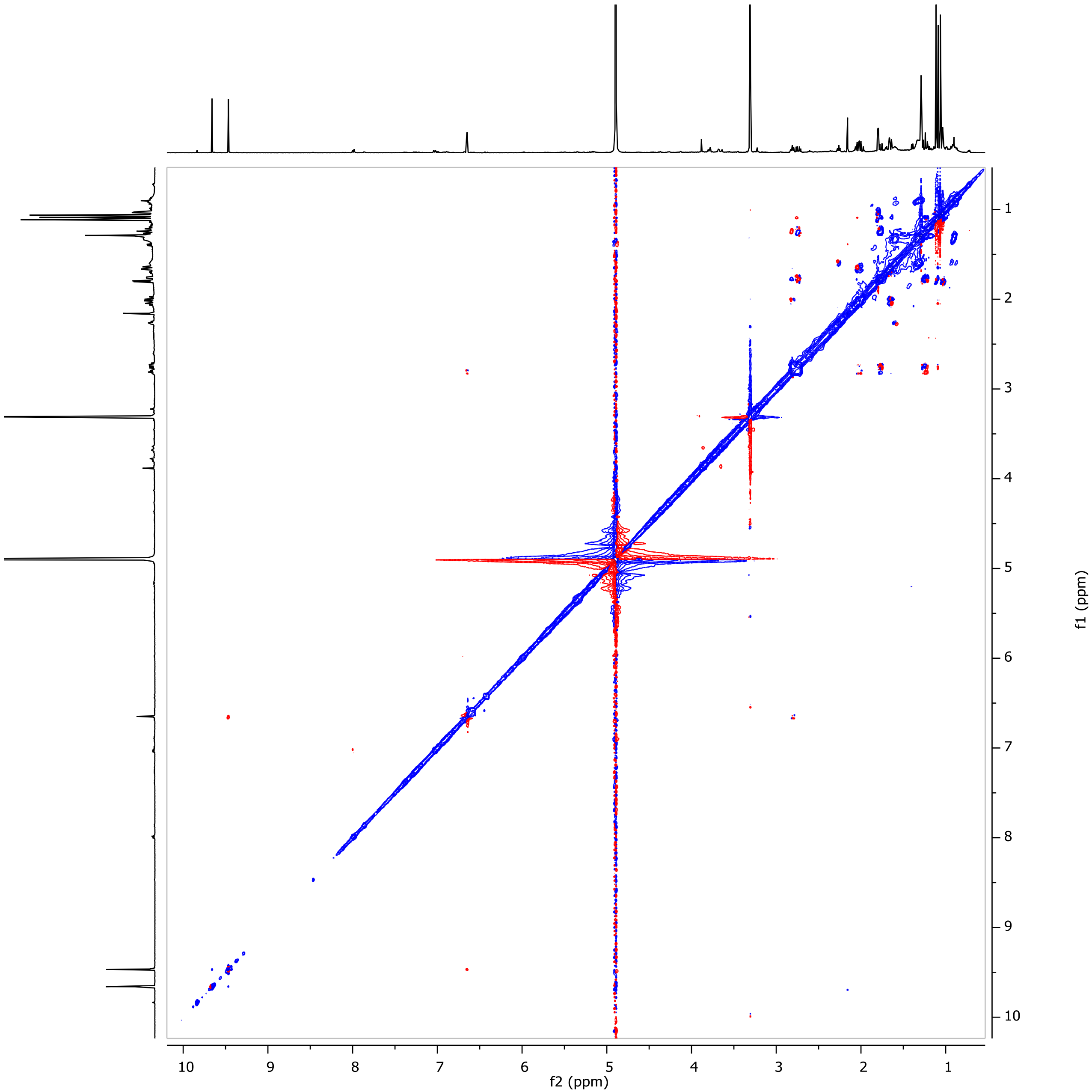

Figure S64. ROESY spectrum of **8** in methanol-d_4 at 500 MHz.

Generic Display Report

Analysis Info

Analysis Name	S:\PEOPLE\sel22_Sherif Elsayed\Bondarzewia\AmaZon\IHI 766R2F15_GE7_01_50349.d	Acquisition Date	02.09.2023 12:43:58
Method	50349.m	Operator	tti
Sample Name	IHI 766R2F15	Instrument	amaZon speed
Comment			

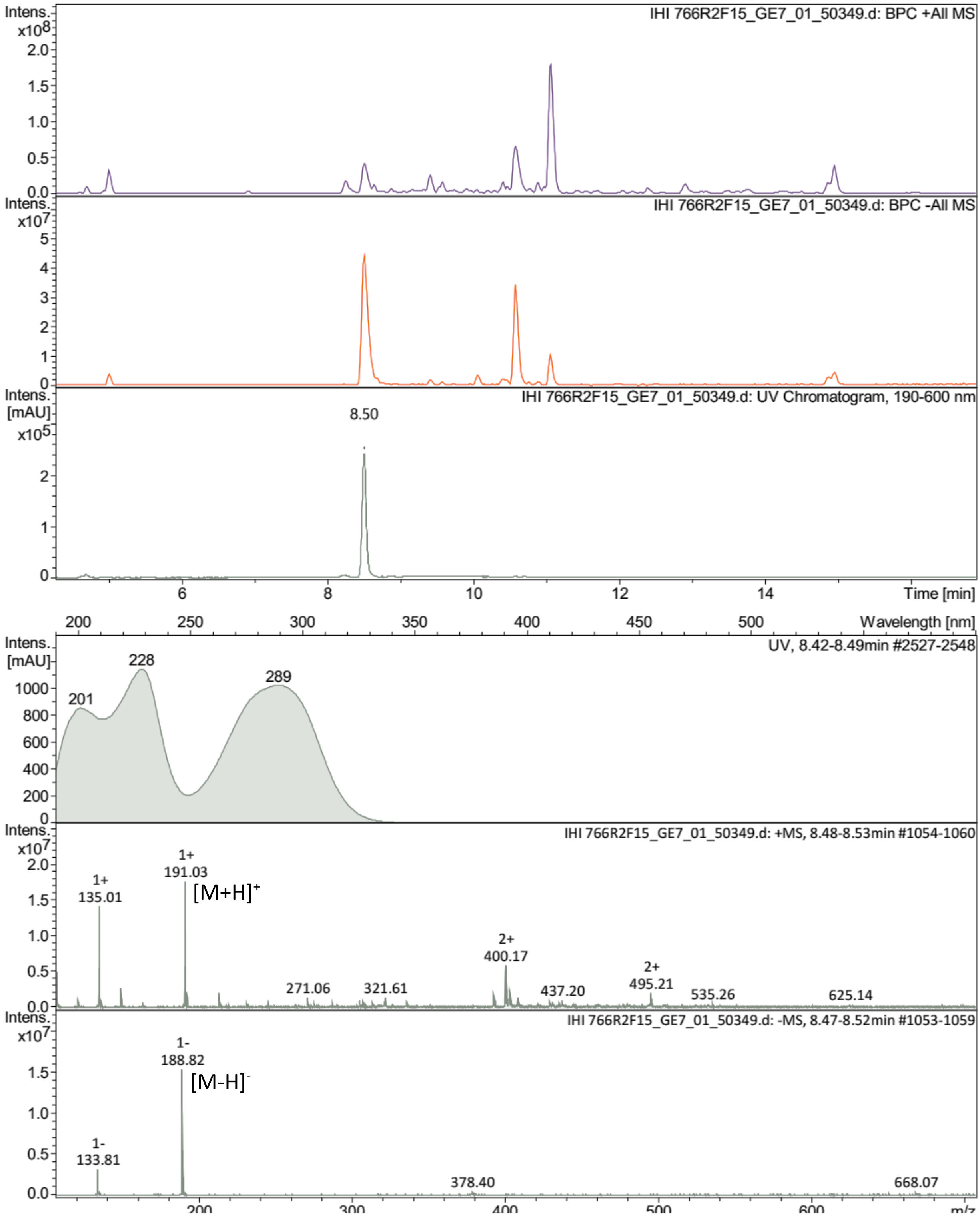

Bruker Compass DataAnalysis 4.4 printed: 11.10.2023 17:55:13 by: sel22 Page 1 of 1

Figure S65. LRESIMS of **9**.

Generic Display Report

Analysis Info

Analysis Name	F:\Volume D\HZI Projects\Winnie\8-Bondarzewia mesenterica\Bondarzewia\MaXis\IHI 766 R2F15_28_01_13124.d	Acquisition Date	05.09.2023 20:53:28
Method	pos_sauce_100_screening_ms_100_2500_line.m	Operator	ate06
Sample Name	IHI 766 R2F15	Instrument	maXis
Comment	Screening01<br>Waters Acquity UPLC BEH C_18 1,7um 2.1x50mm		

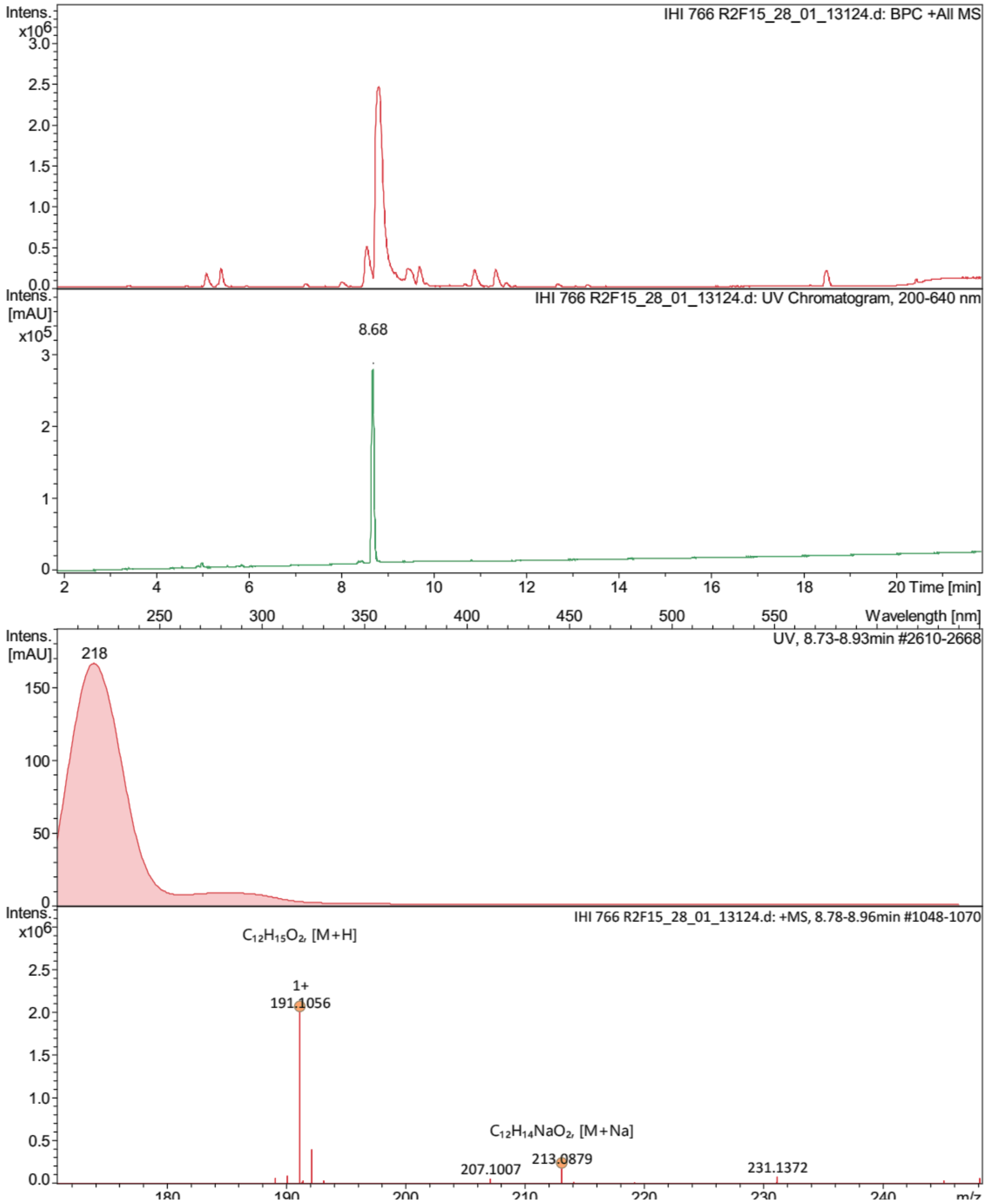

Figure S66. HRESIMS of **9**.

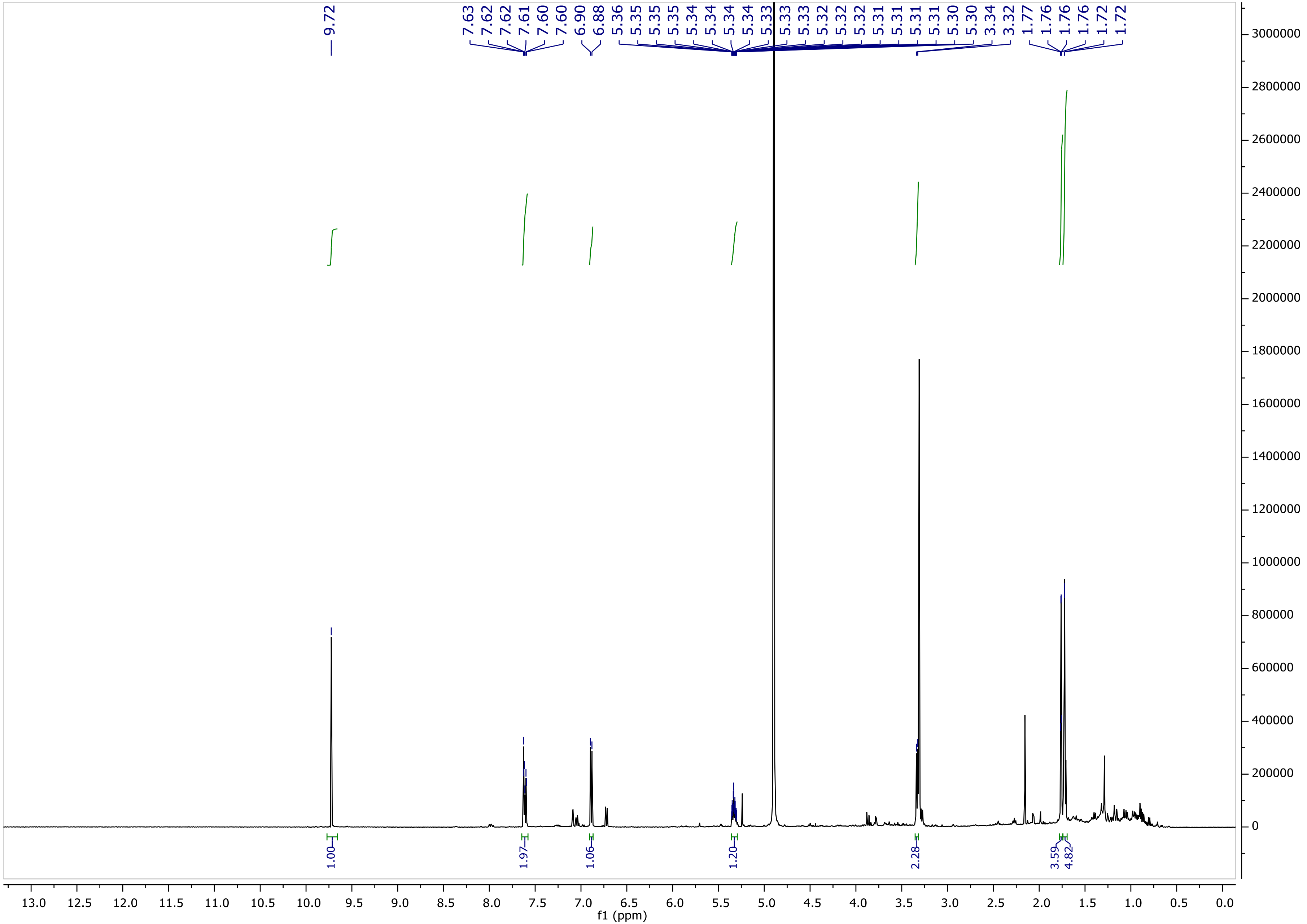

Figure S67. ^{1}H NMR spectrum of **9** in methanol-d_4 at 500 MHz.

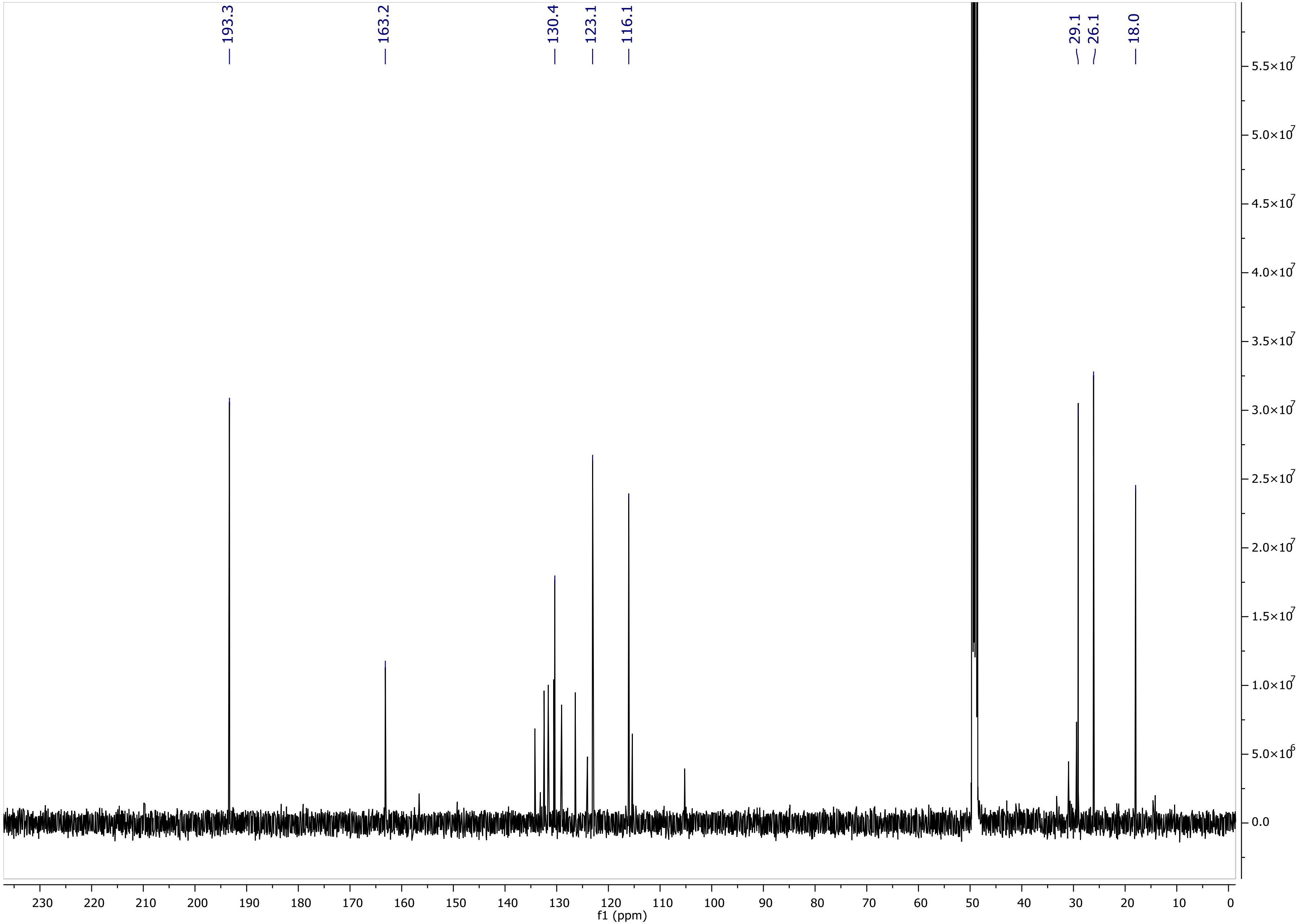

Figure S68. ^{13}C NMR spectrum of **9** in methanol-d_4 at 125 MHz.

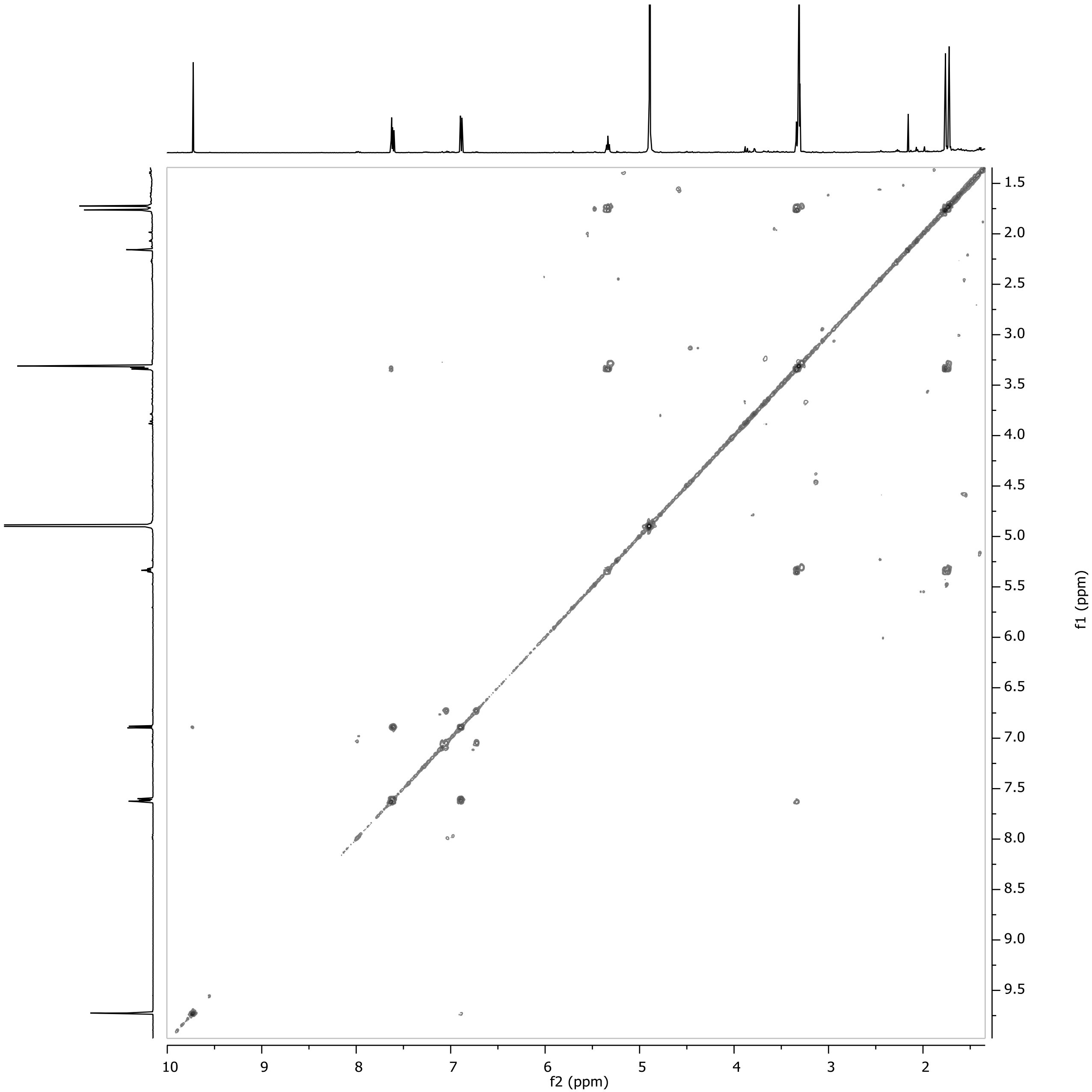

Figure S69. ^{1}H-^{1}H COSY spectrum of **9** in methanol-d_4 at 500 MHz.

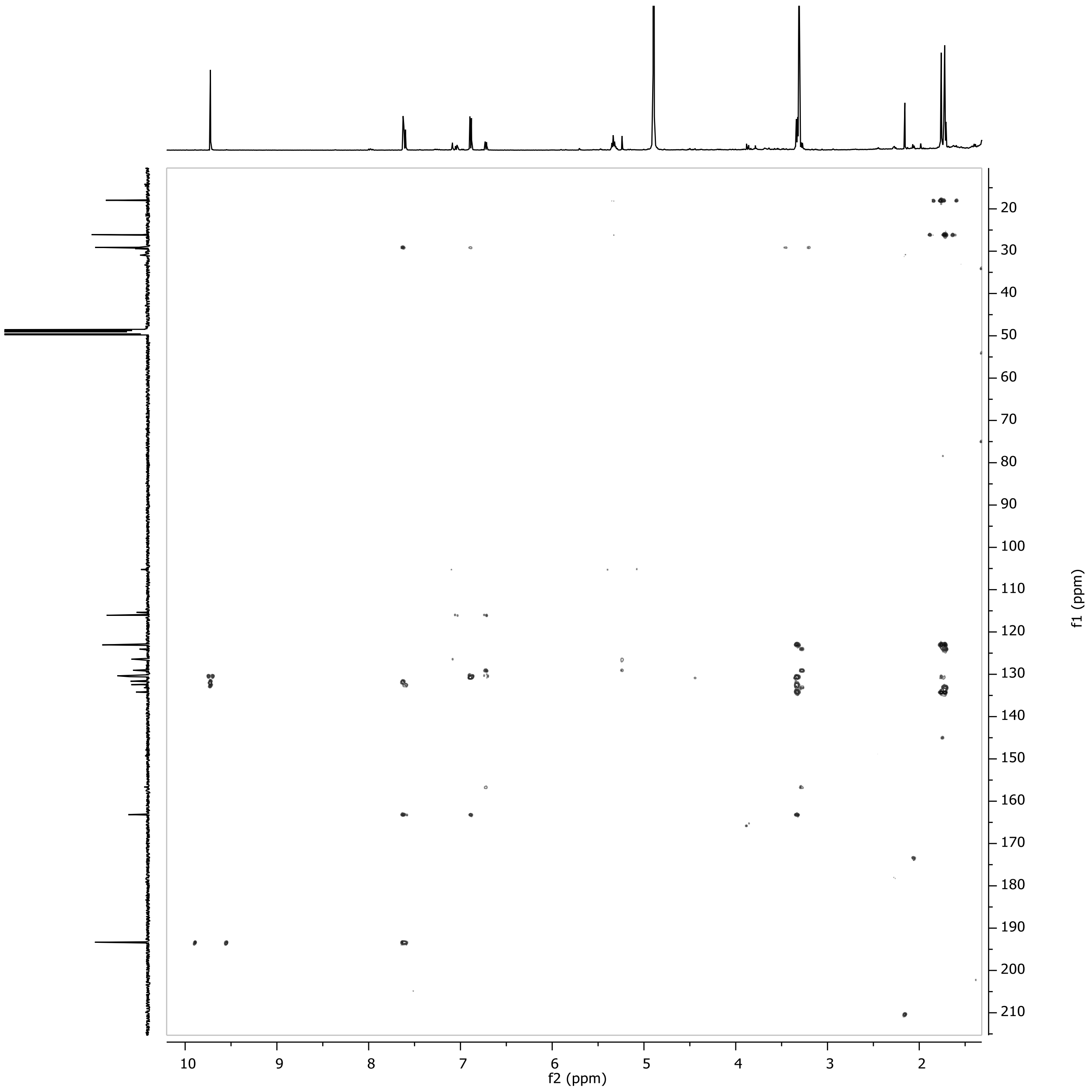

Figure S70. HMBC spectrum of **9** in methanol-d_4 at 500 MHz.

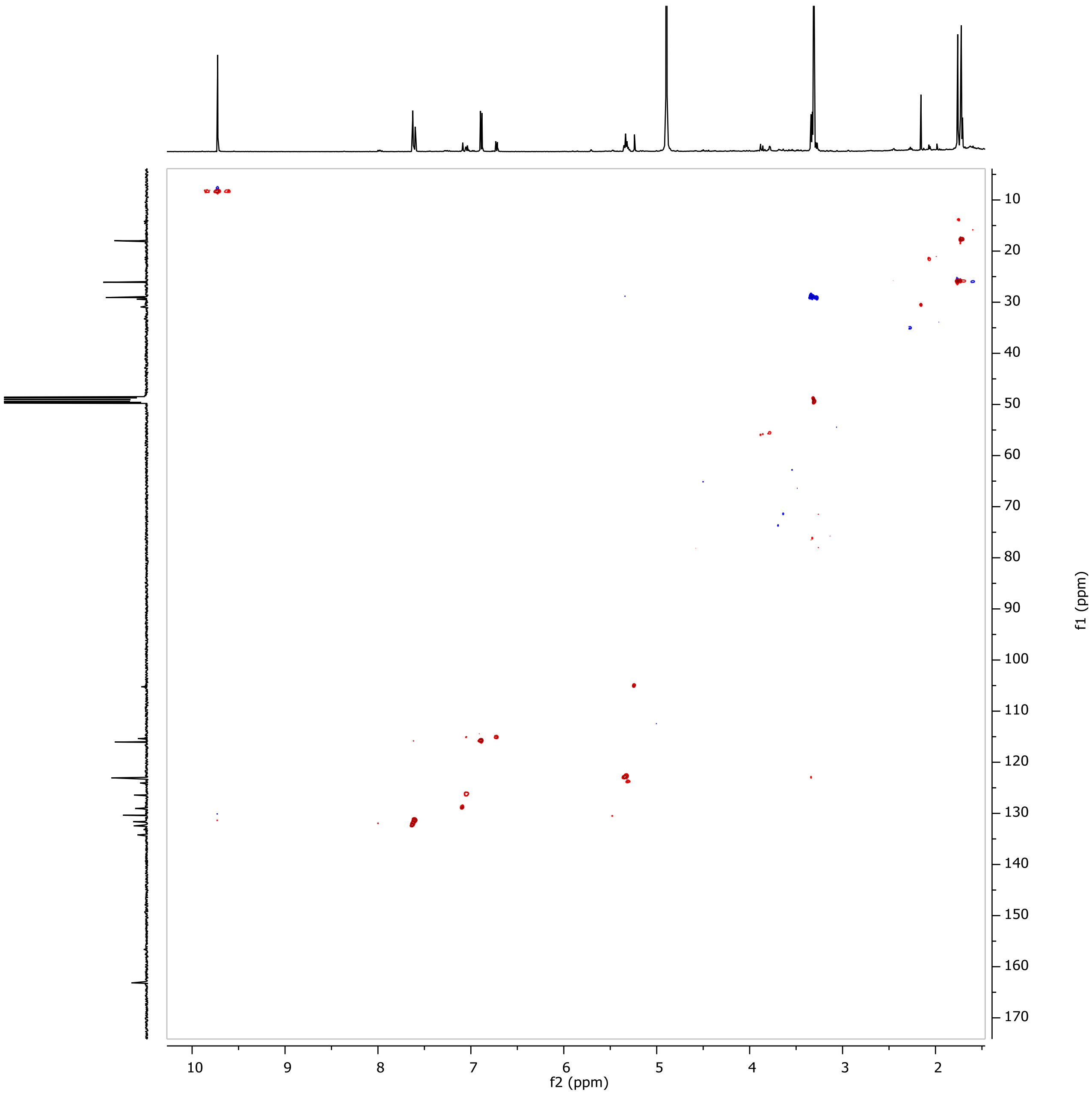

Figure S71. HSQC spectrum of **9** in methanol-d_4 at 500 MHz.

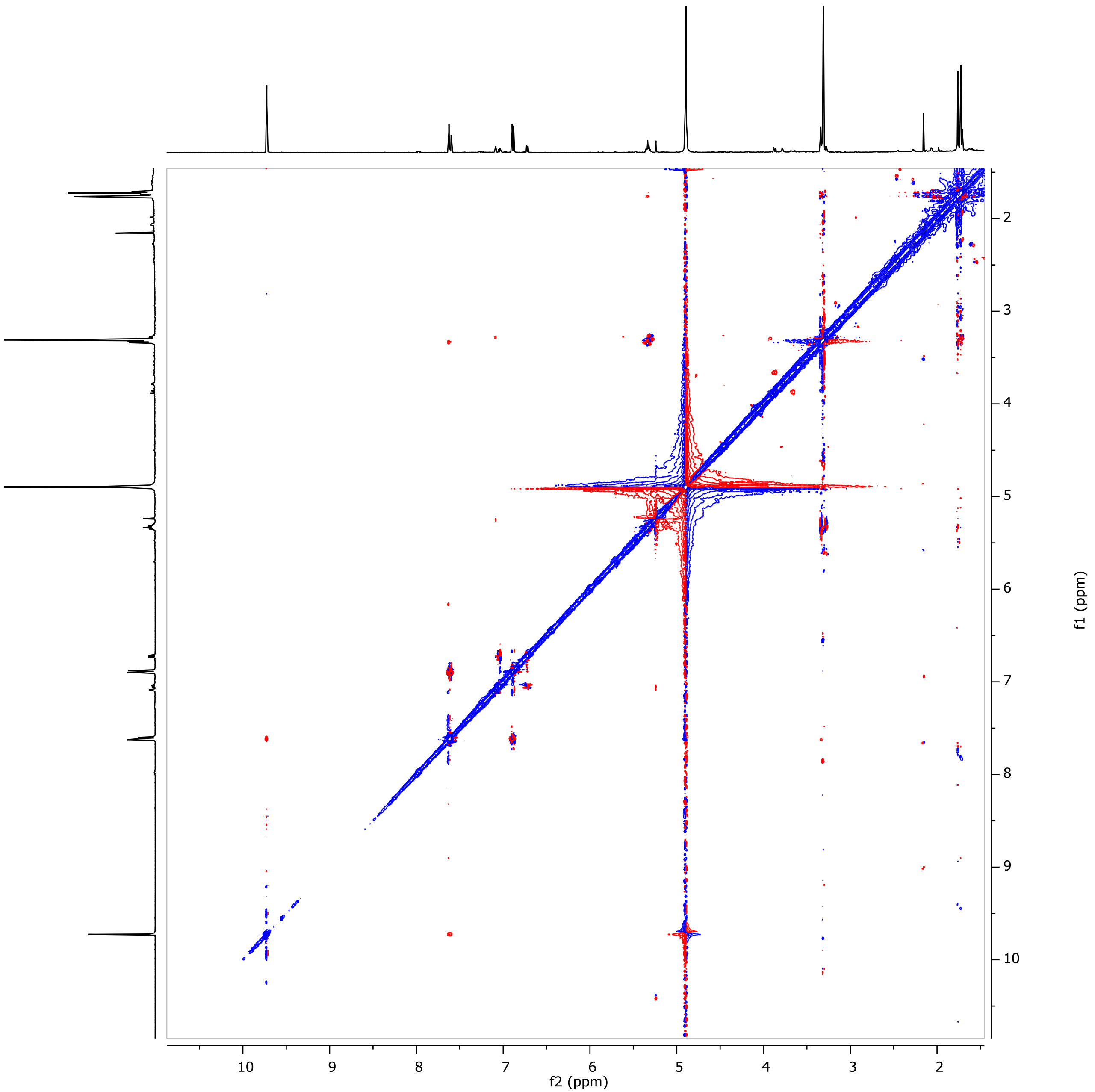

Figure S72. ROESY spectrum of **9** in methanol-d_4 at 500 MHz.

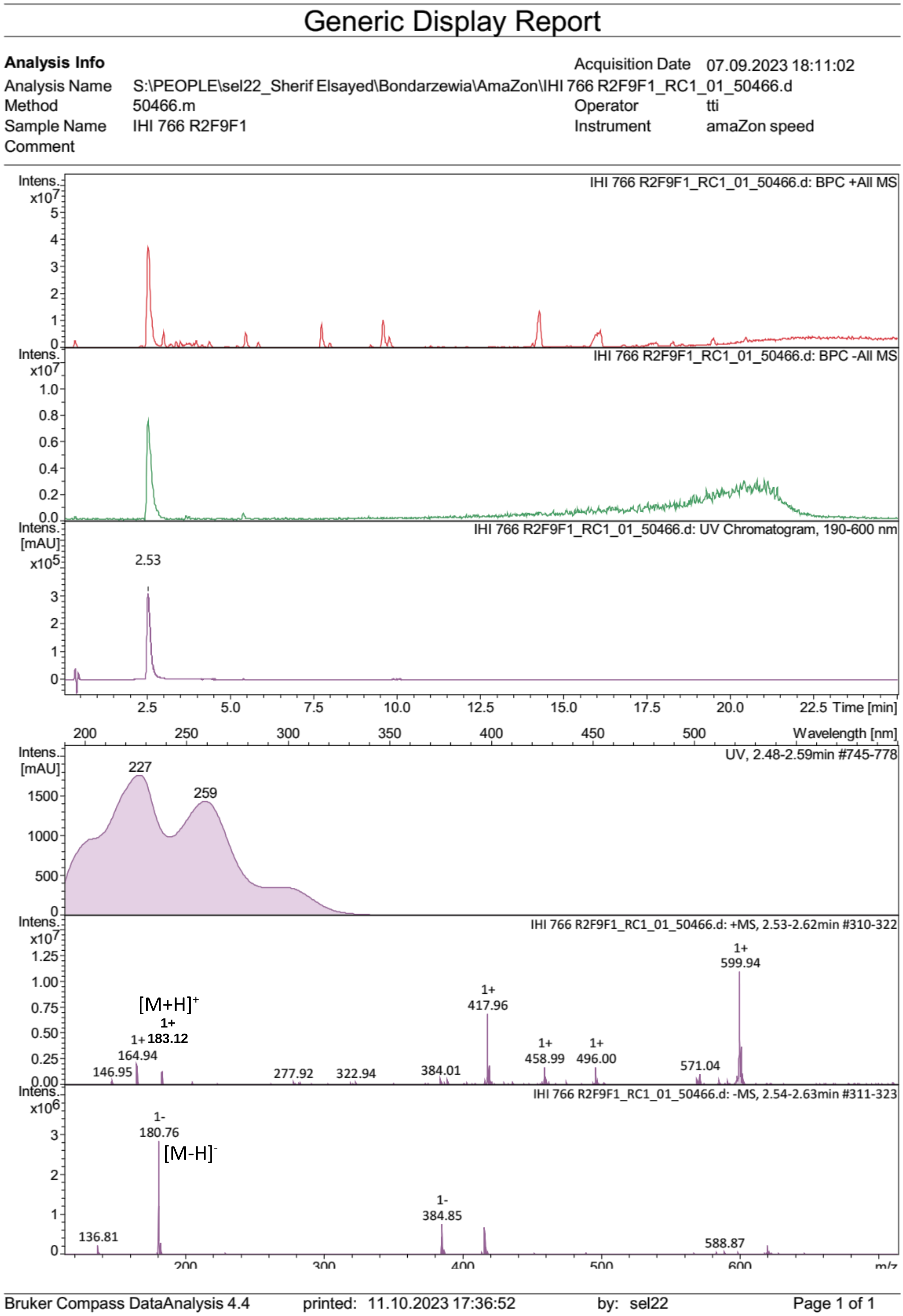

Figure S73. LRESIMS of **10**.

Generic Display Report

Analysis Info

		Acquisition Date	14.09.2023 13:31:25
Analysis Name	S:\PEOPLE\sel22_Sherif Elsayed\Bondarzewia\MaXis\IHI 766 R2F9F1_11_01_13174.d		
Method	pos_säure_10000_screening_ms_100_2500_line.m	Operator	ate06
Sample Name	IHI 766 R2F9F1	Instrument	maXis
Comment	Screening01<br>Waters Acquity UPLC BEH C_18 1,7um 2.1x50mm		

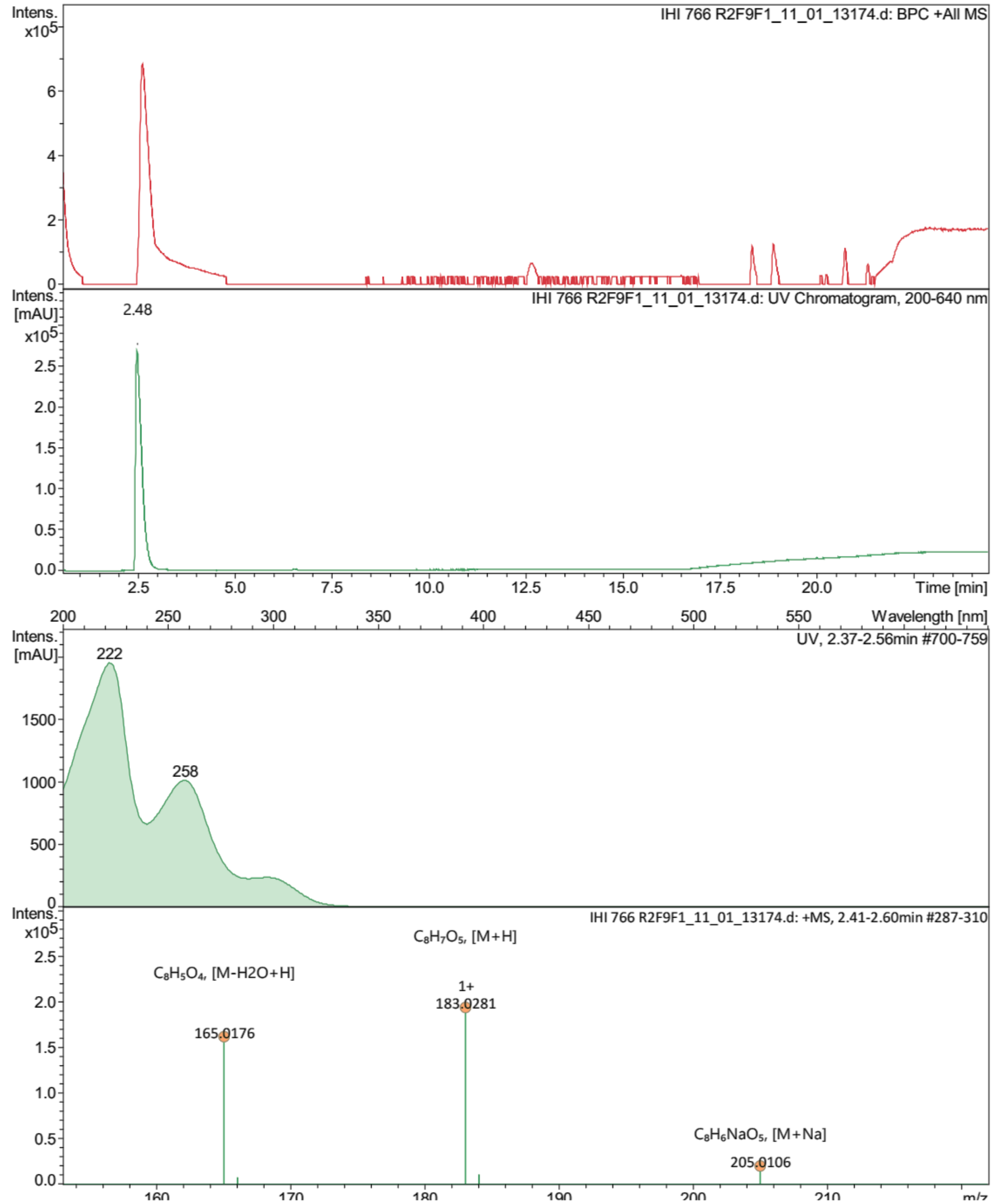

Bruker Compass DataAnalysis 4.4 printed: 11.10.2023 18:08:42 by: sel22 Page 1 of 1

Figure S74. HRESIMS of **10**.

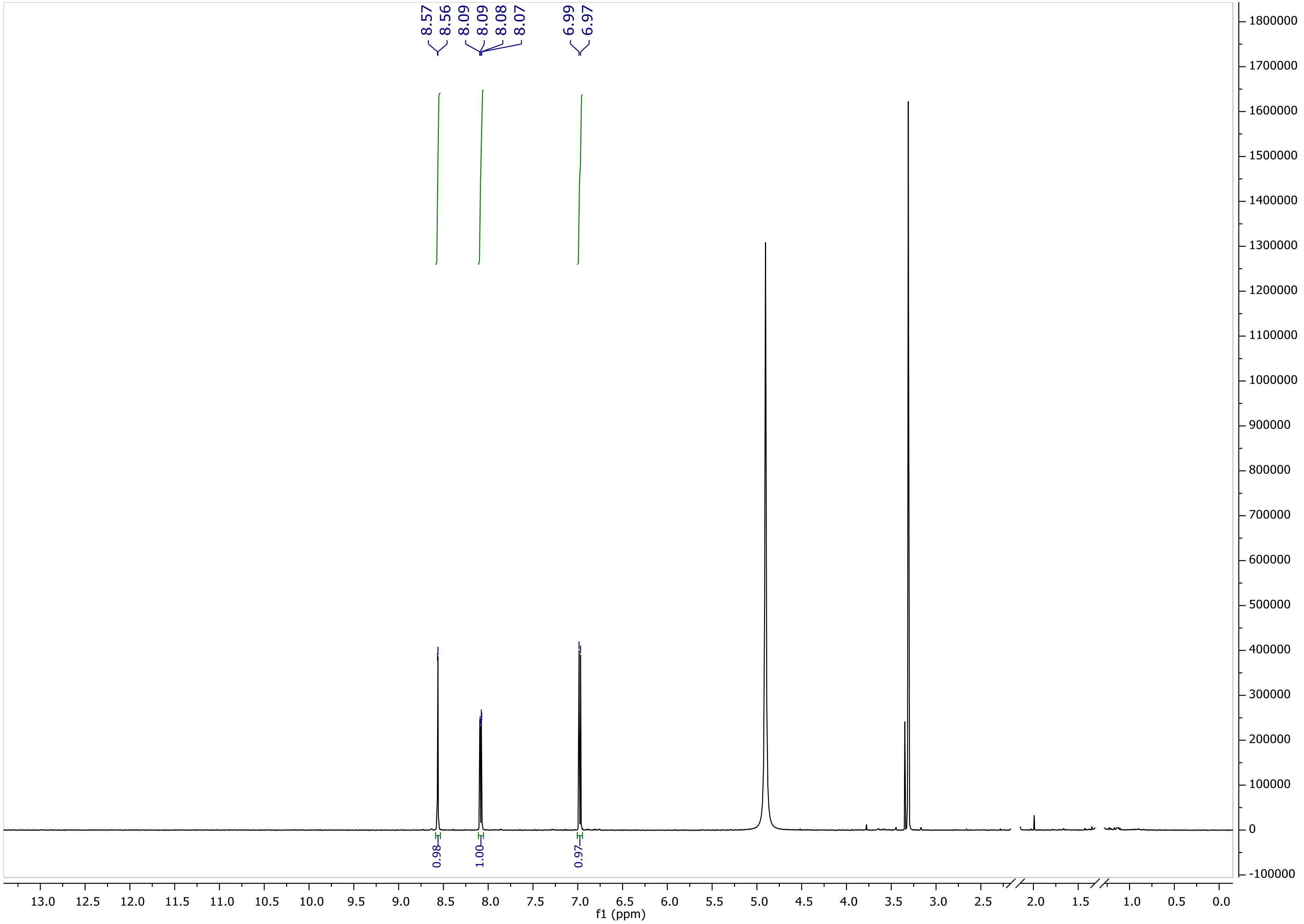

Figure S75. ^{1}H NMR spectrum of **10** in methanol-d_4 at 500 MHz.

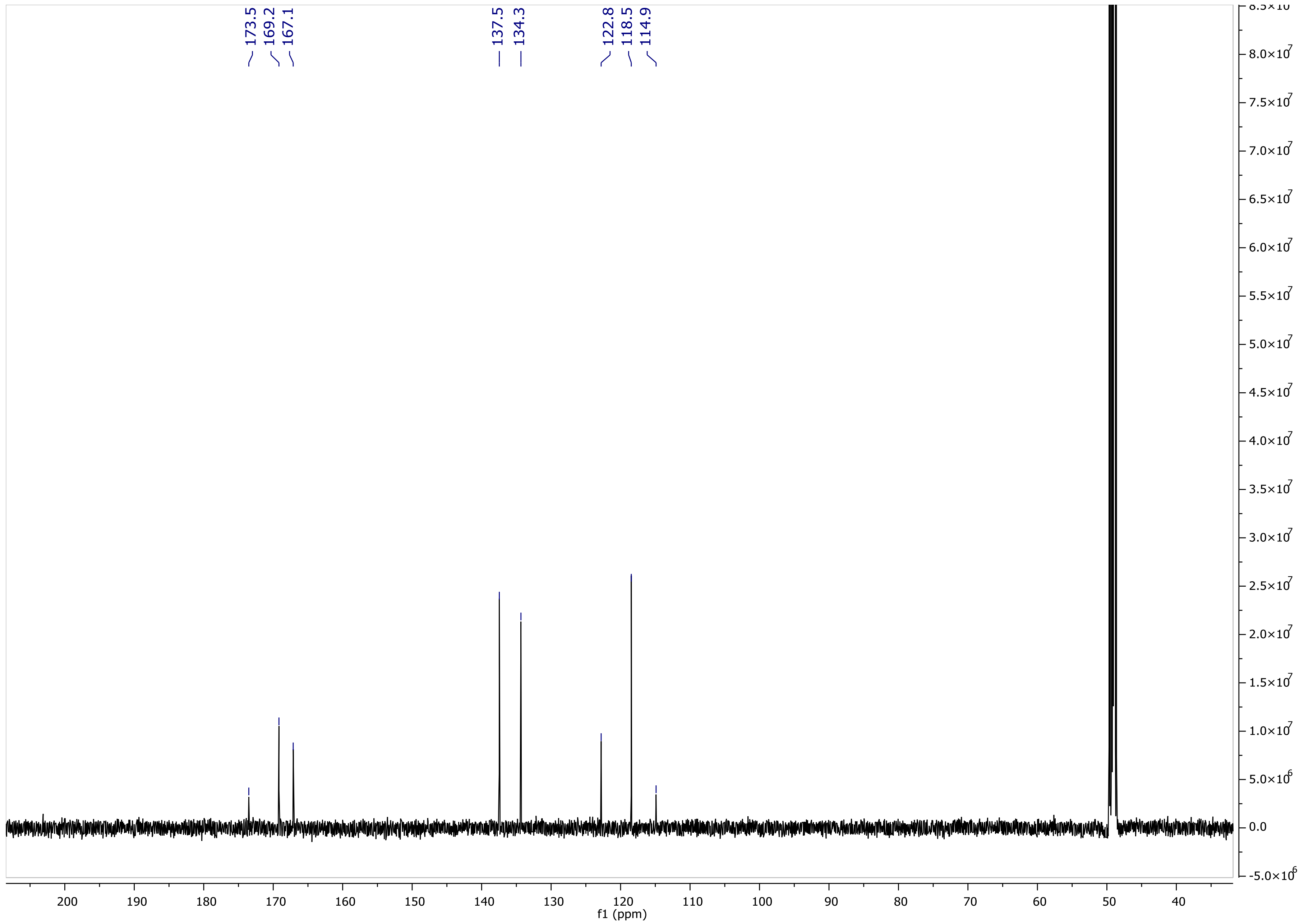

Figure S76. ^{13}C NMR spectrum of **10** in methanol-d_4 at 125 MHz.

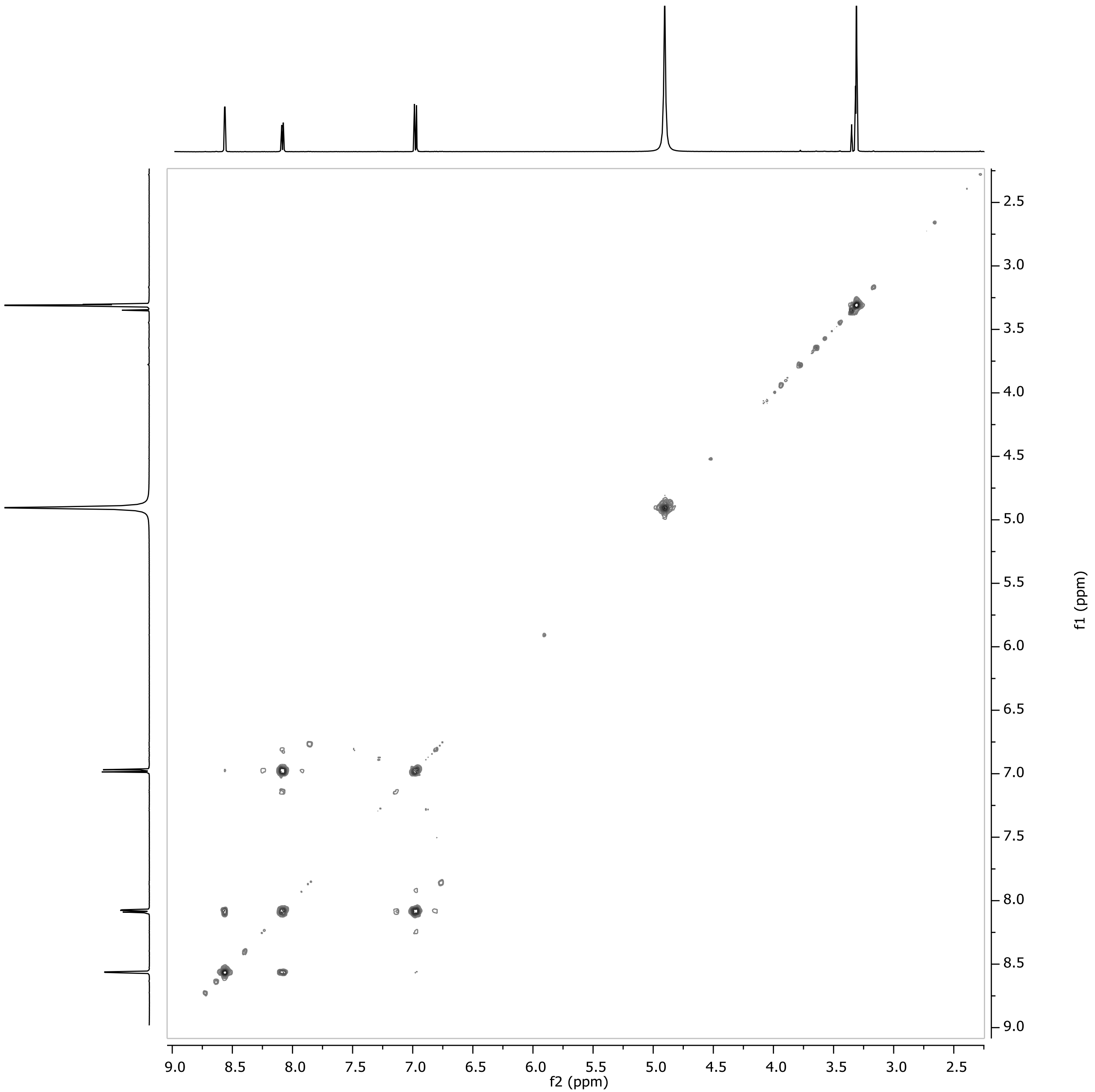

Figure S77. ^{1}H-^{1}H COSY spectrum of **10** in methanol-d_4 at 500 MHz.

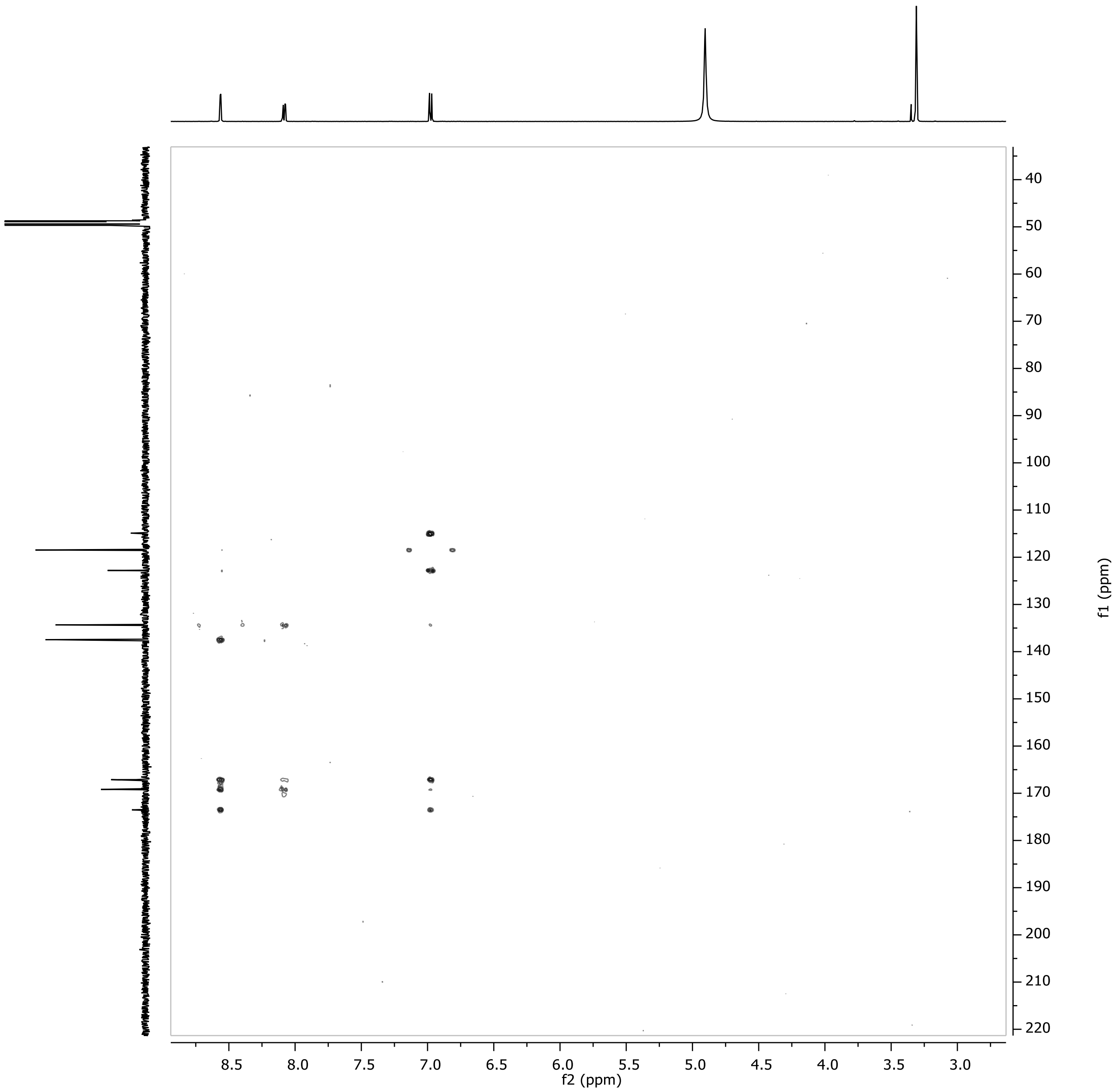

Figure S78. HMBC spectrum of **10** in methanol-d_4 at 500 MHz.

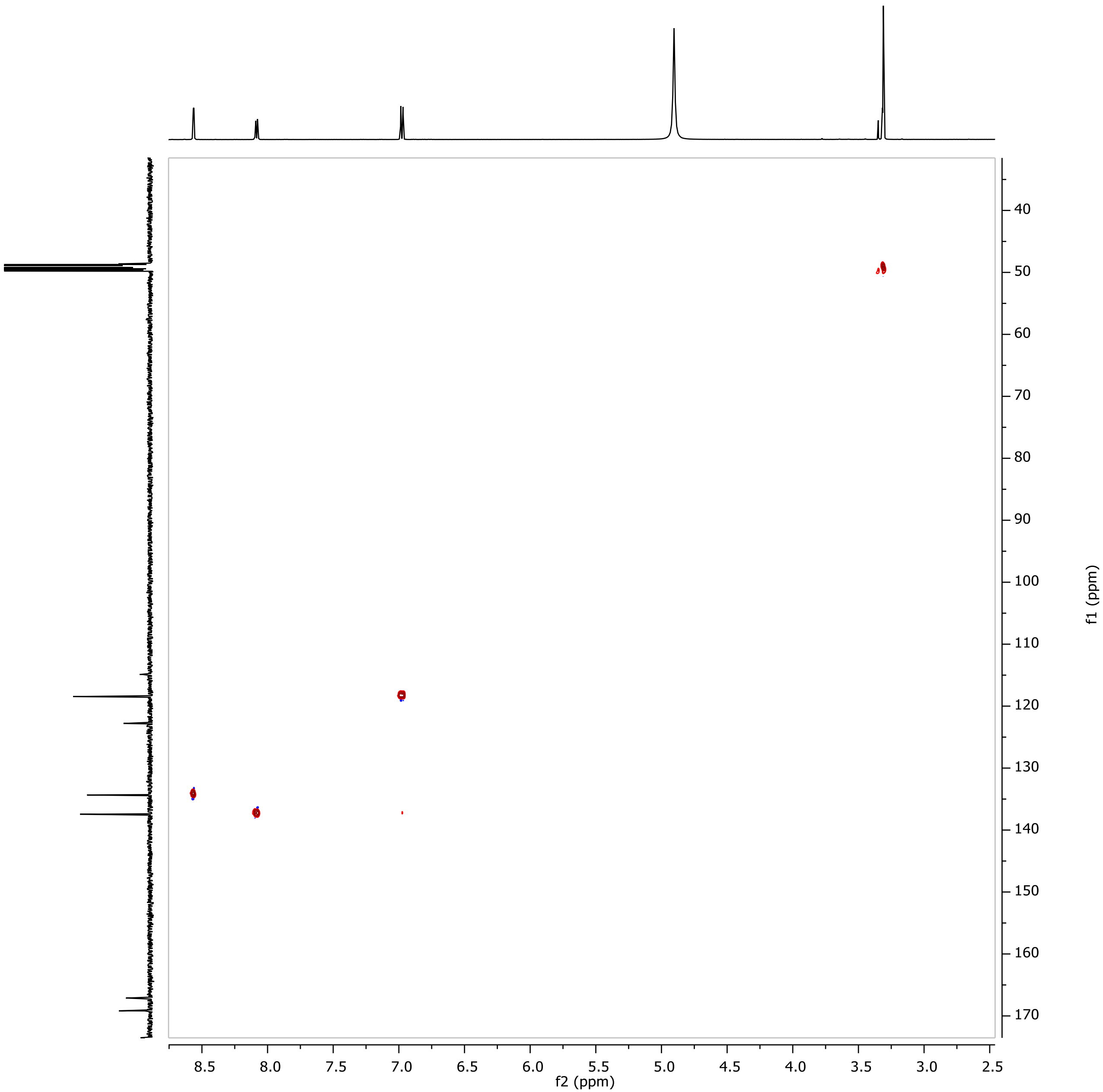

Figure S79. HSQC spectrum of **10** in methanol-d_4 at 500 MHz.

Generic Display Report

Analysis Info

Acquisition Date	02.09.2023 16:21:38
Analysis Name	S:\PEOPLE\sel22_Sherif Elsayed\Bondarzewia\AmaZon\IHI 766R2F19_GA6_01_50355.d
Method	50355.m
Operator	tti
Sample Name	IHI 766R2F19
Instrument	amaZon speed
Comment	

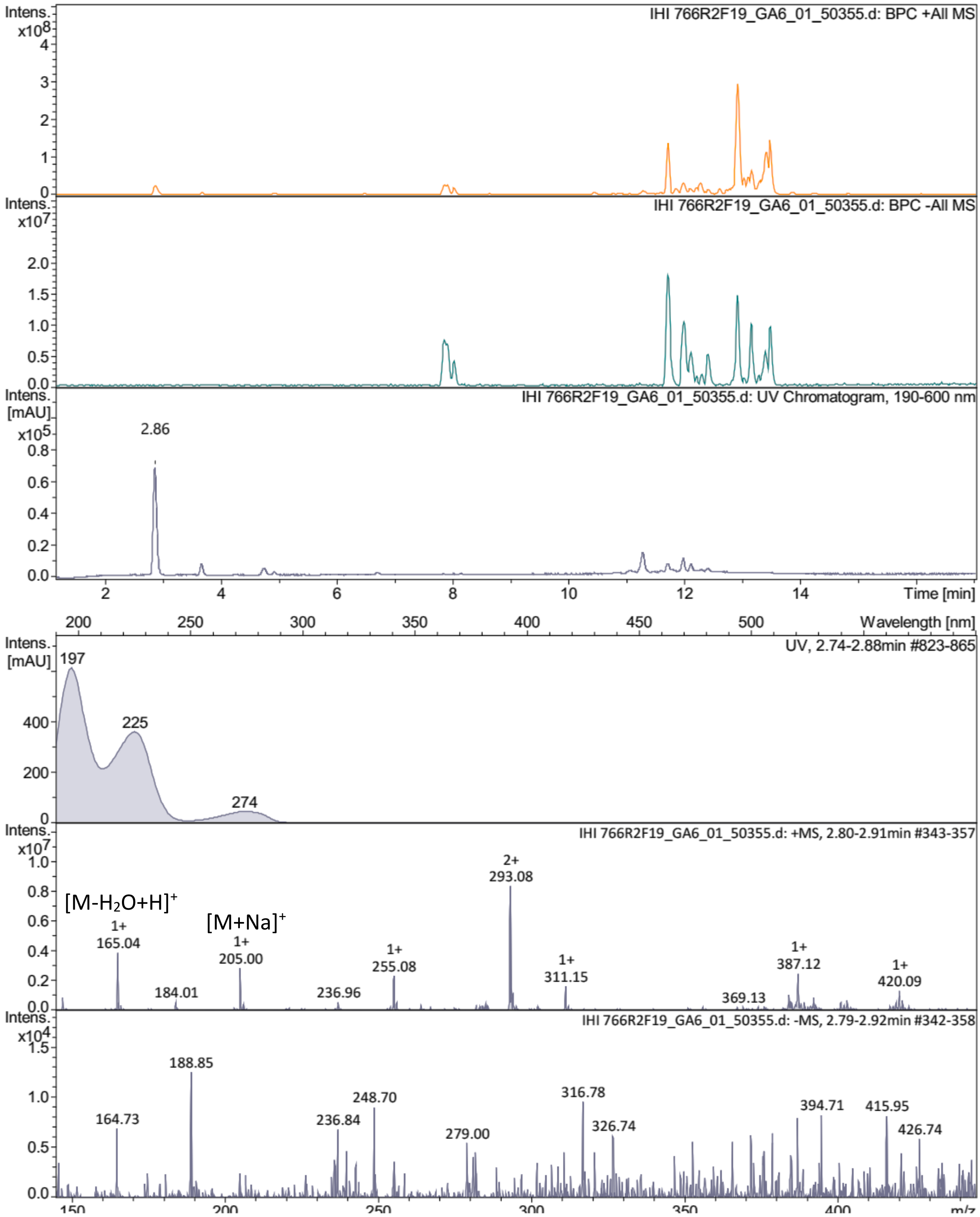

Bruker Compass DataAnalysis 4.4 printed: 11.10.2023 17:59:04 by: sel22 Page 1 of 1

Figure S80. LRESIMS of **11**.

Generic Display Report

Analysis Info

Analysis Name	F:\Volume D\HZI Projects\Winnie\8-Bondarzewia mesenterica\Bondarzewia\MaXis\IHI 766 R2F19_30_01_13126.d	Acquisition Date	05.09.2023 21:55:25
Method	pos_[illegible]screening_ms_100_2500_line.m	Operator	ate06
Sample Name	IHI 766 R2F19	Instrument	maXis
Comment	Screening01<br>Waters Acquity UPLC BEH C_18 1,7um 2.1x50mm		

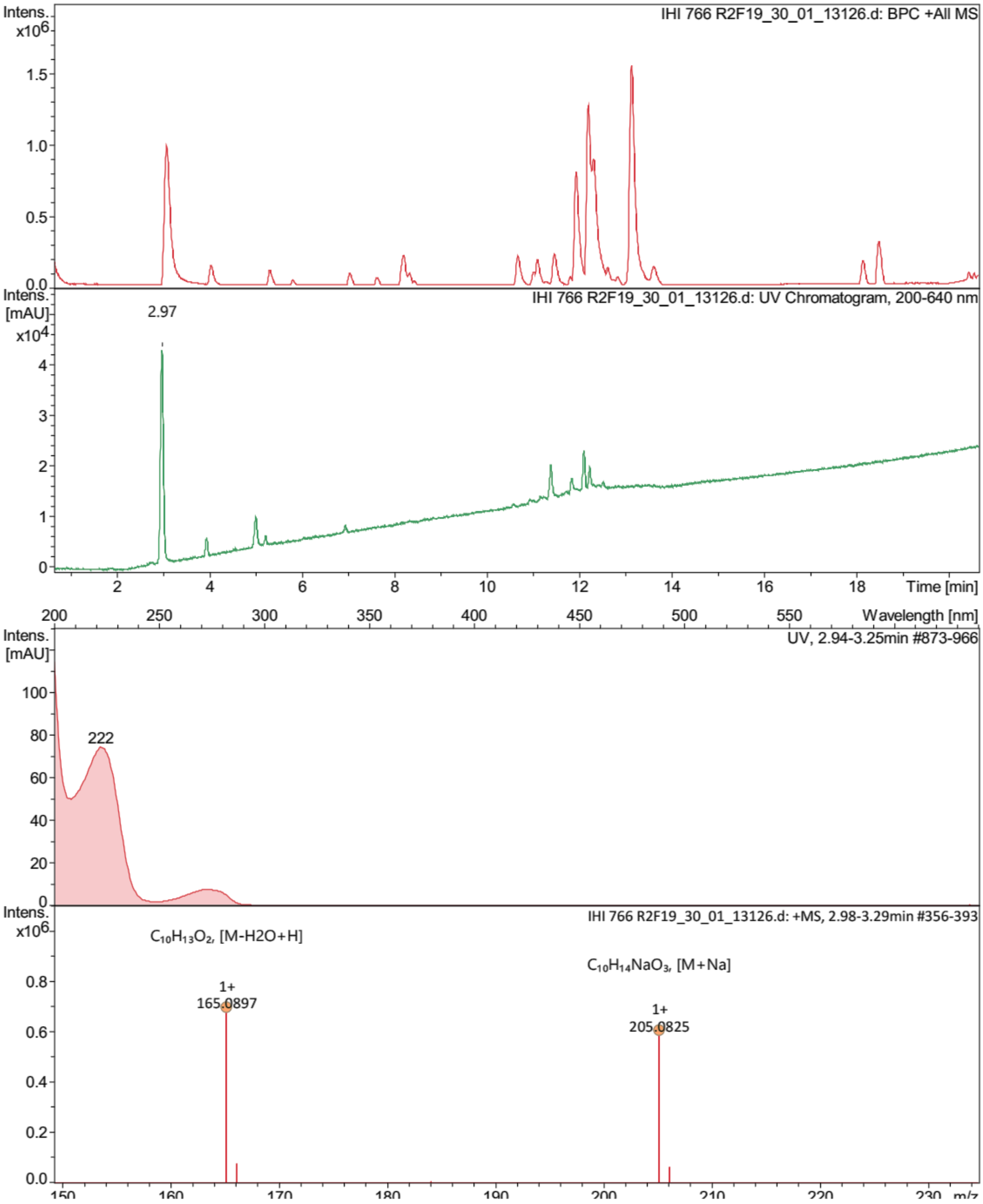

Bruker Compass DataAnalysis 4.4 printed: 11.10.2023 16:28:45 by: sel22 Page 1 of 1

Figure S81. HRESIMS of **11**.

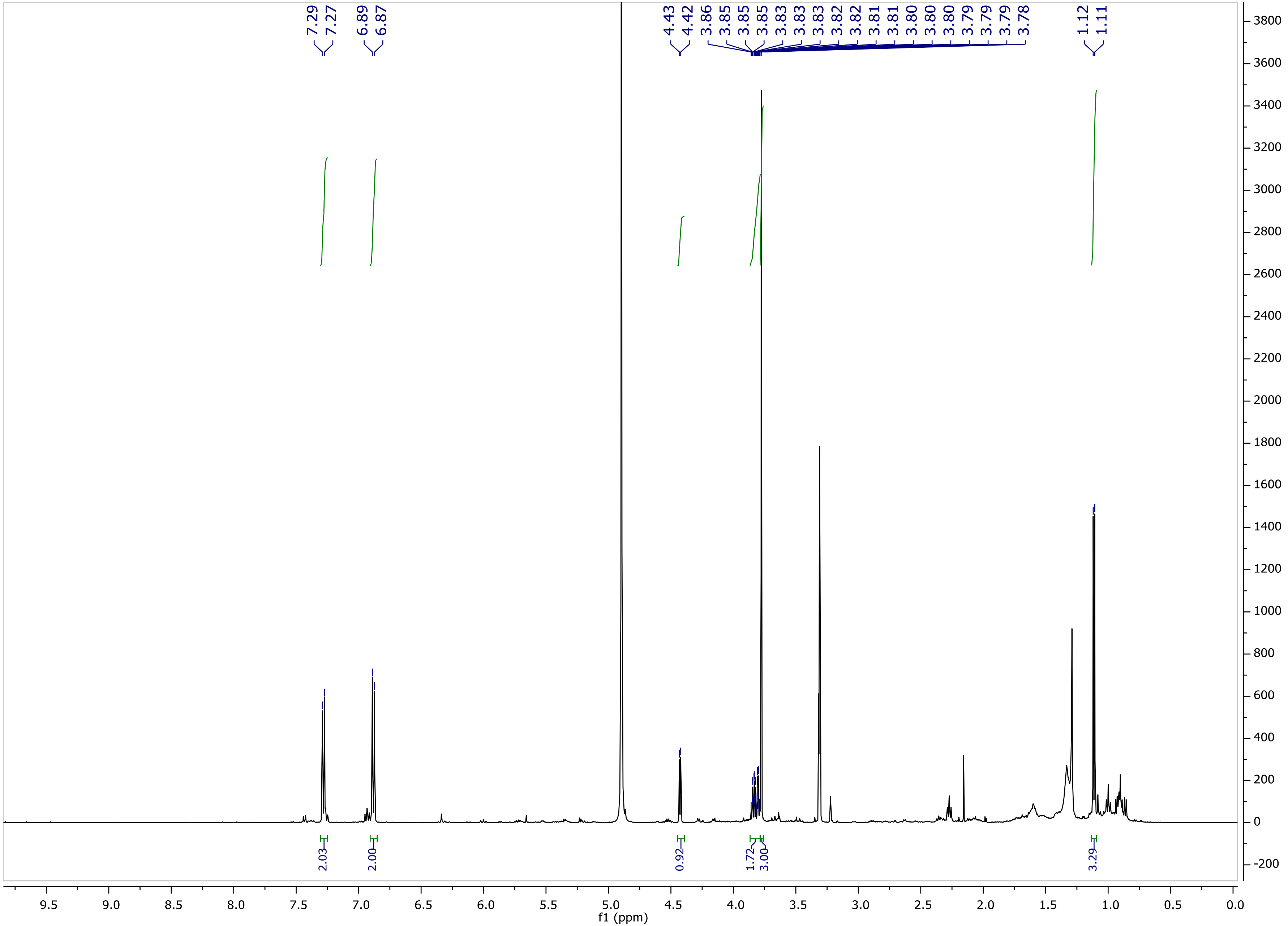

Figure S82. ^{1}H NMR spectrum of **11** in methanol-d_4 at 500 MHz.

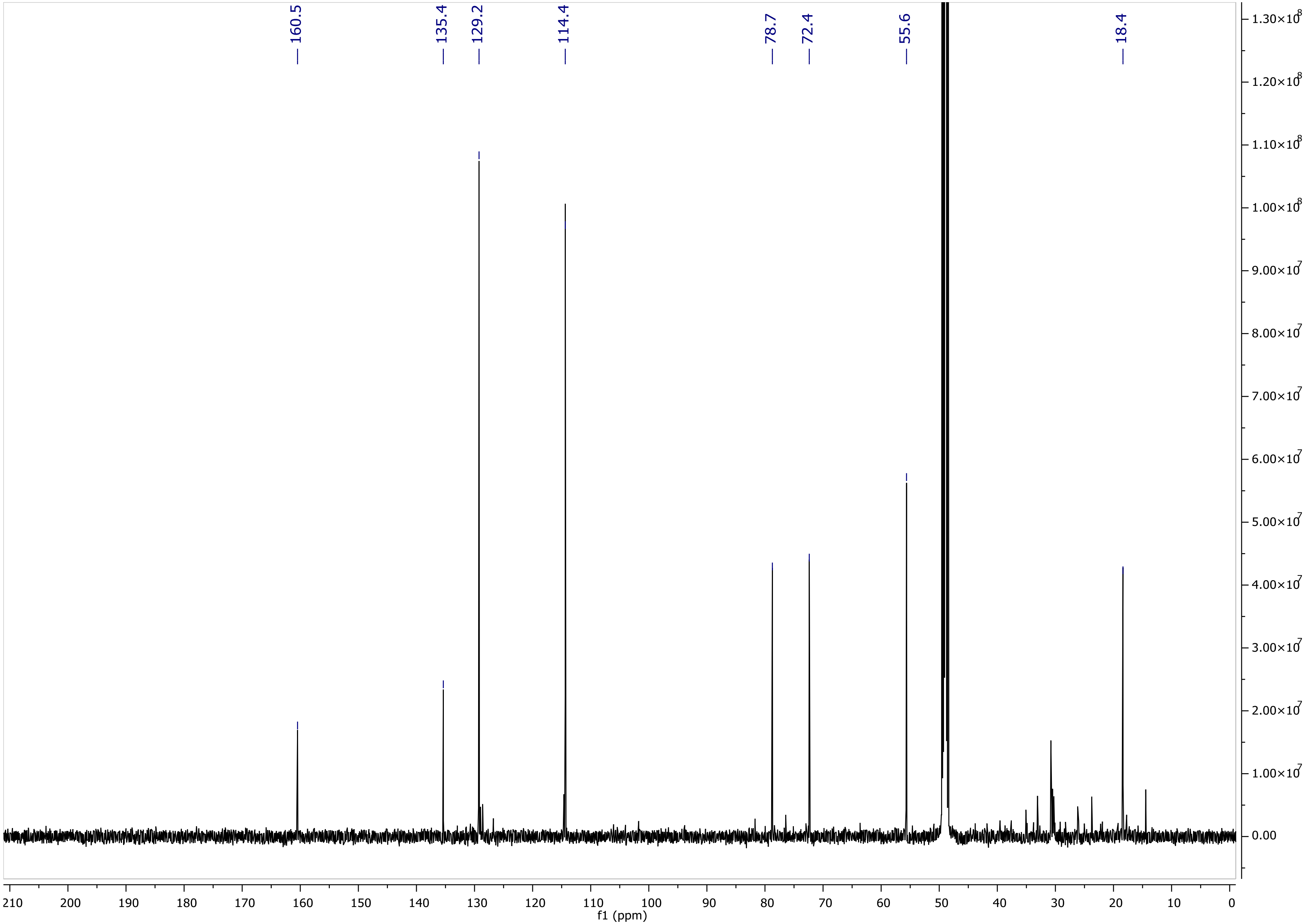

Figure S83. ^{13}C NMR spectrum of **11** in methanol-d_4 at 125 MHz.

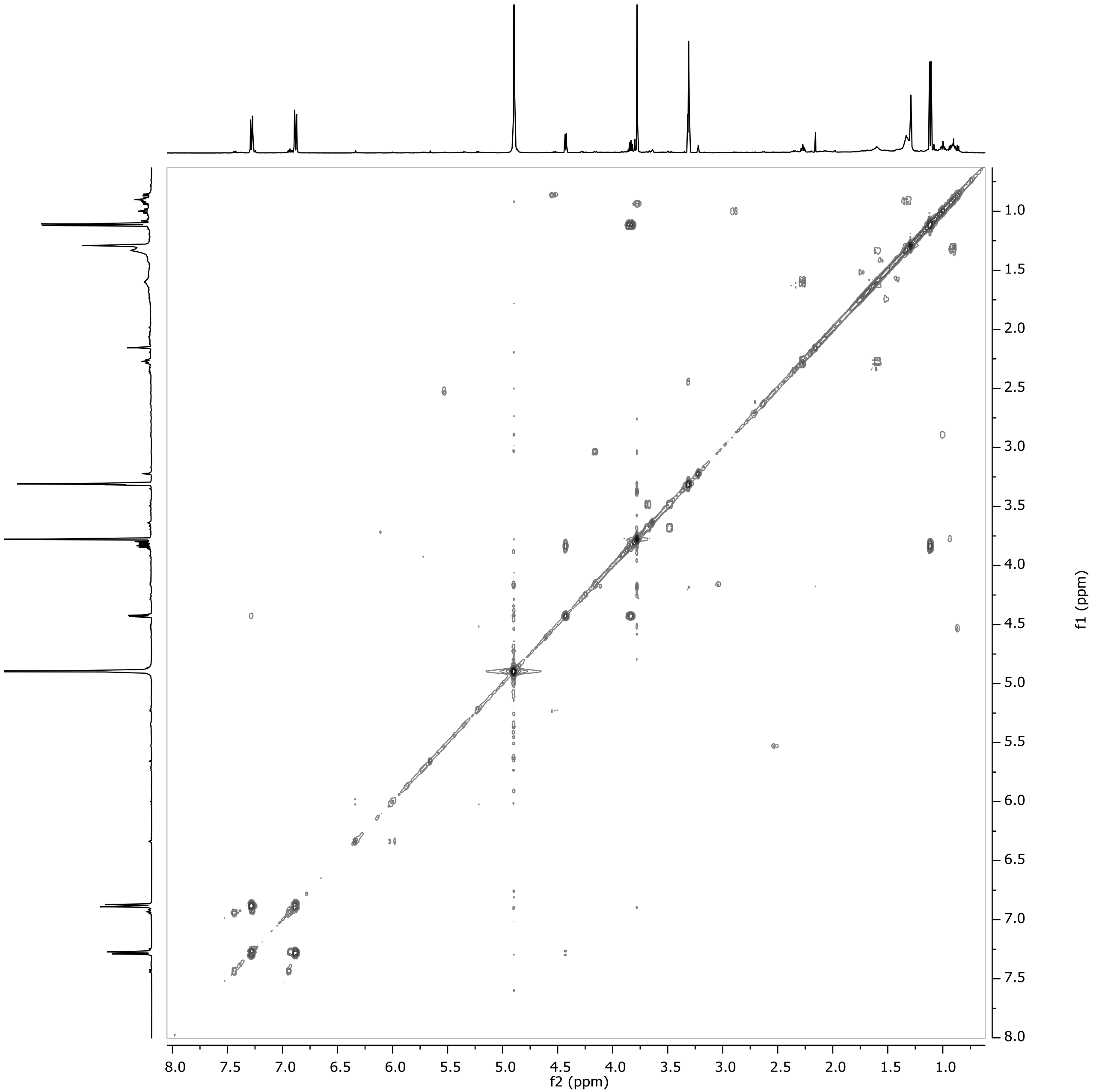

Figure S84. ^{1}H-^{1}H COSY spectrum of **11** in methanol-d_4 at 500 MHz.

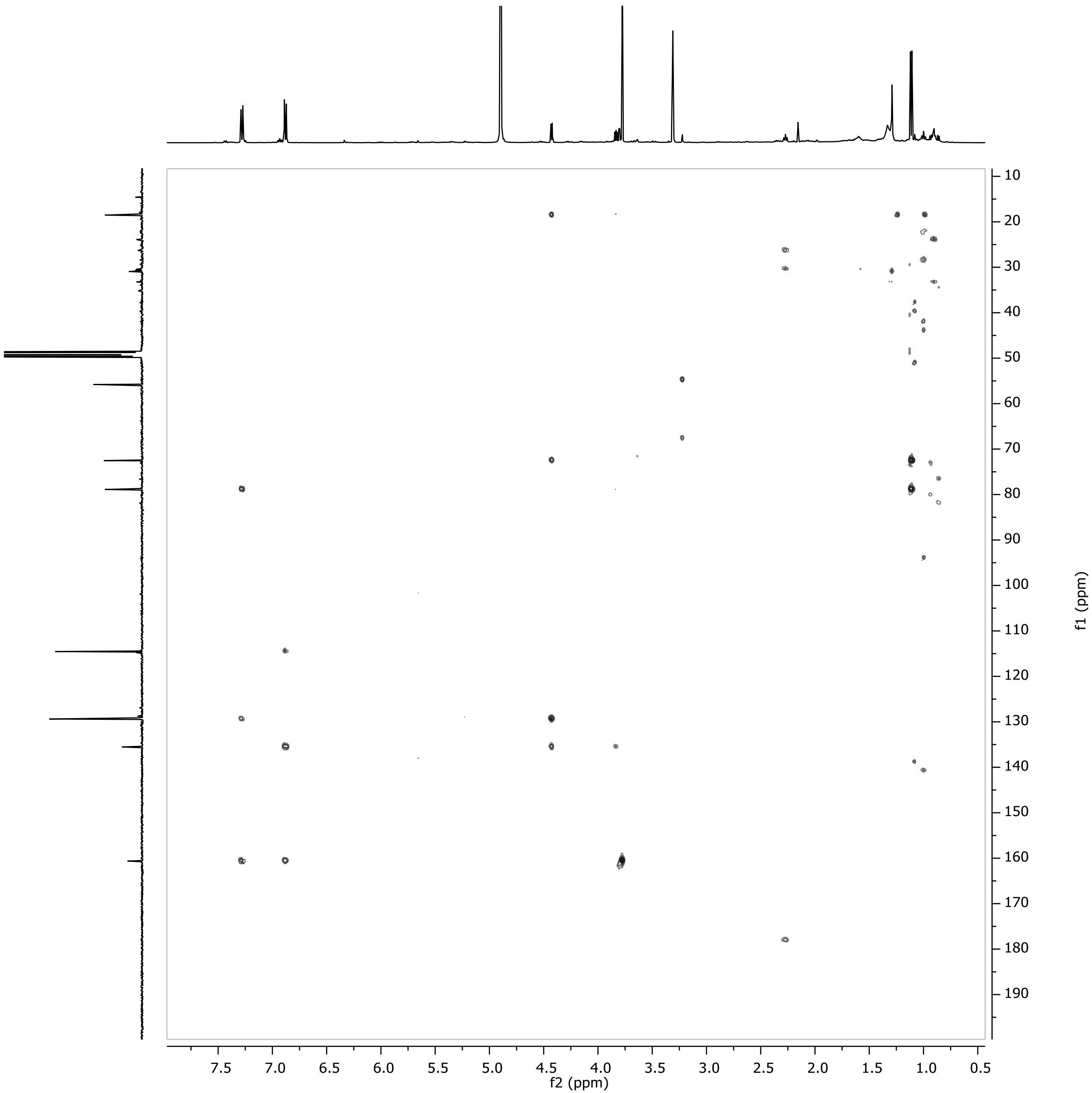

Figure S85. HMBC spectrum of **11** in methanol-d_4 at 500 MHz.

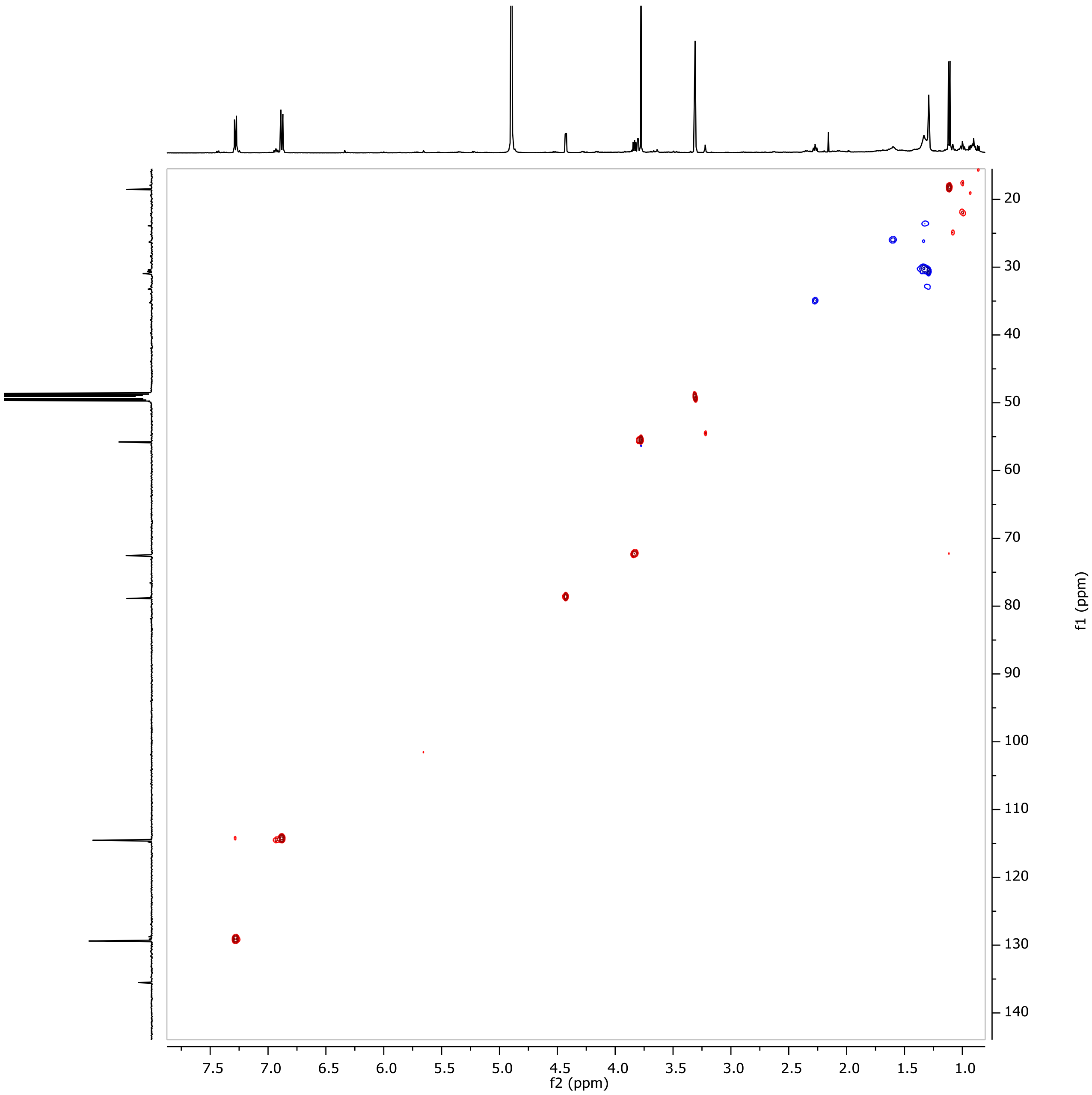

Figure S86. HSQC spectrum of **11** in methanol-d_4 at 500 MHz.

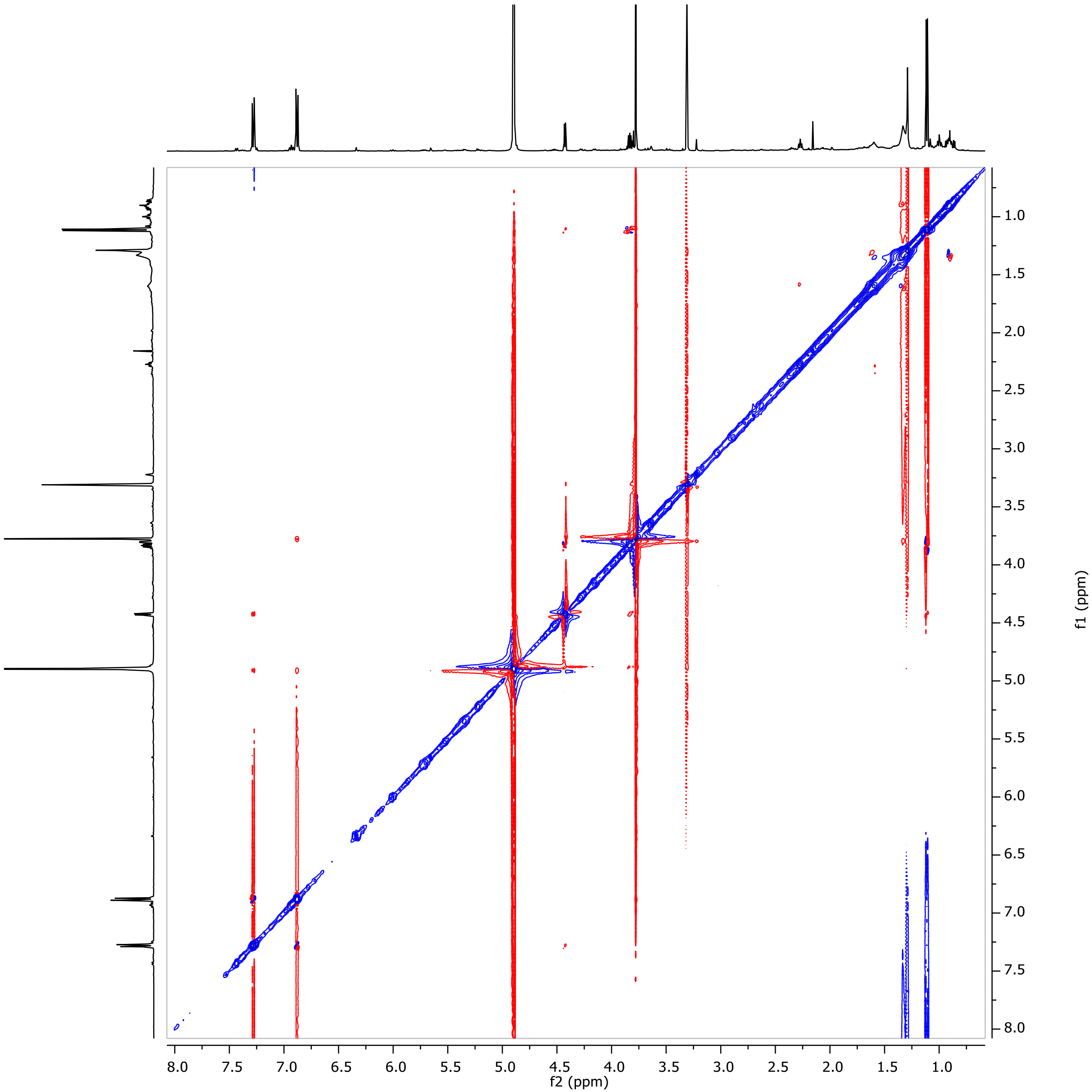

Figure S87. ROESY spectrum of **11** in methanol-d_4 at 500 MHz.

Generic Display Report

Analysis Info

Analysis Name	S:\PEOPLE\sel22_Sherif Elsayed\Bondarzewia\AmaZon\IHI 766R2F10_GE2_01_50344.d	Acquisition Date	02.09.2023 09:42:32
Method	50344.m	Operator	tti
Sample Name	IHI 766R2F10	Instrument	amaZon speed
Comment			

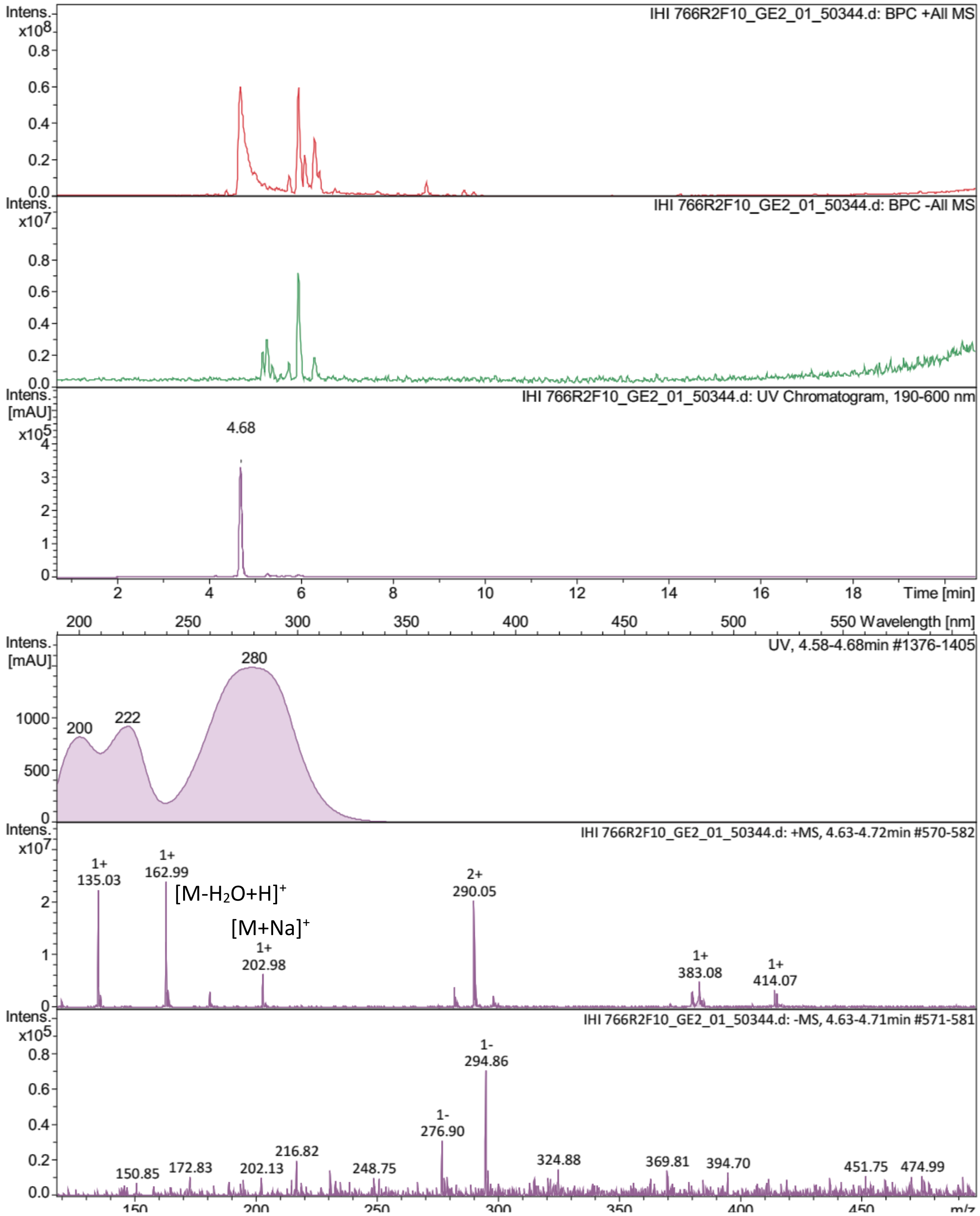

Bruker Compass DataAnalysis 4.4 printed: 11.10.2023 17:50:48 by: sel22 Page 1 of 1

Figure S88. LRESIMS of **12**.

Generic Display Report

Analysis Info

Analysis Name	F:\Volume D\HZI Projects\Winnie\8-Bondarzewia mesenterica\Bondarzewia\MaXis\IHI 766 R2F10_25_01_13121.d	Acquisition Date	05.09.2023 19:20:34
Method	[illegible]creening_ms_100_2500_line.m	Operator	ate06
Sample Name	IHI 766 R2F10	Instrument	maXis
Comment	Screening01<br>Waters Acquity UPLC BEH C_18 1,7um 2.1x50mm		

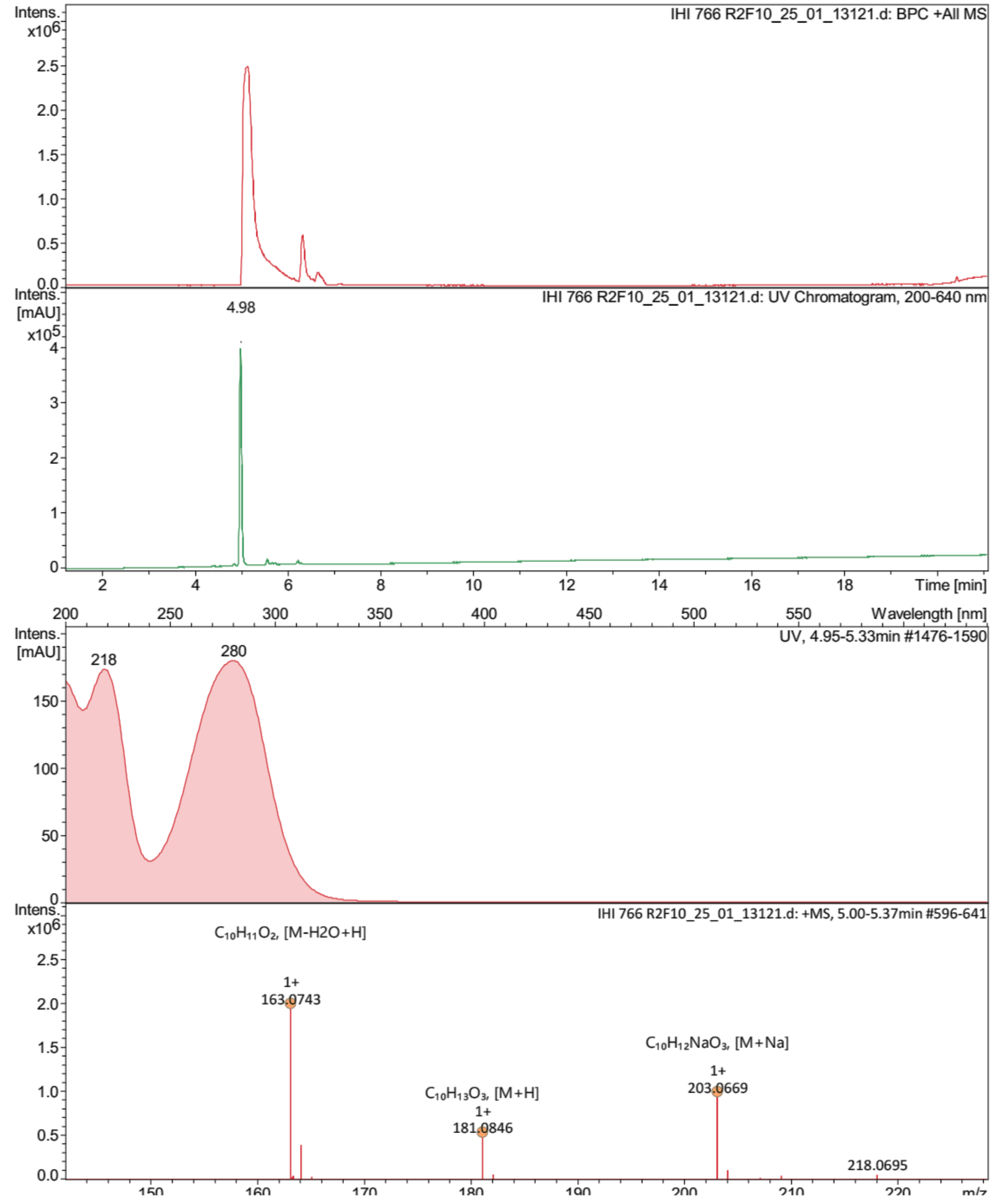

Figure S89. HRESIMS of **12**.

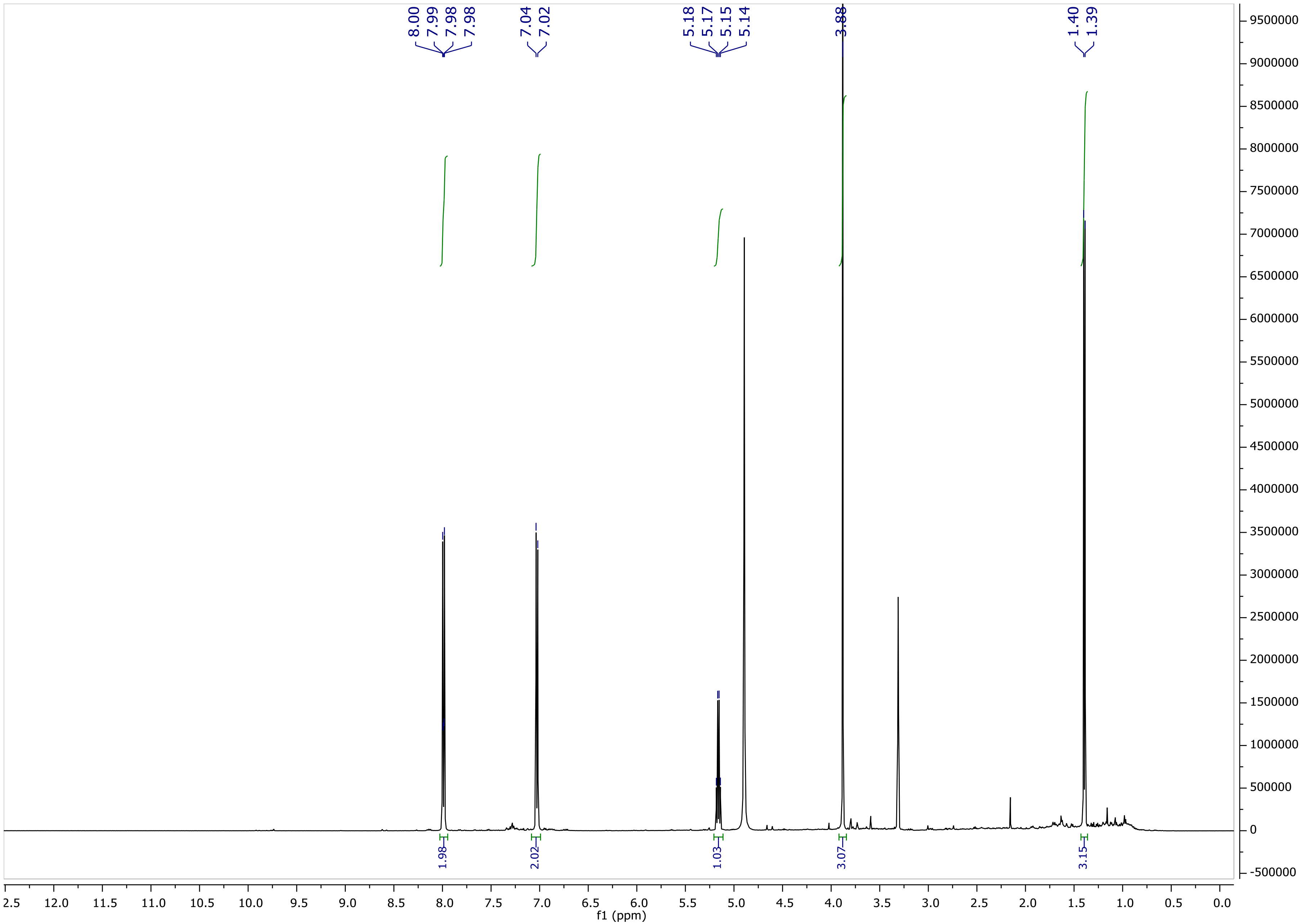

Figure S90. ^{1}H NMR spectrum of **12** in methanol-d_4 at 500 MHz.

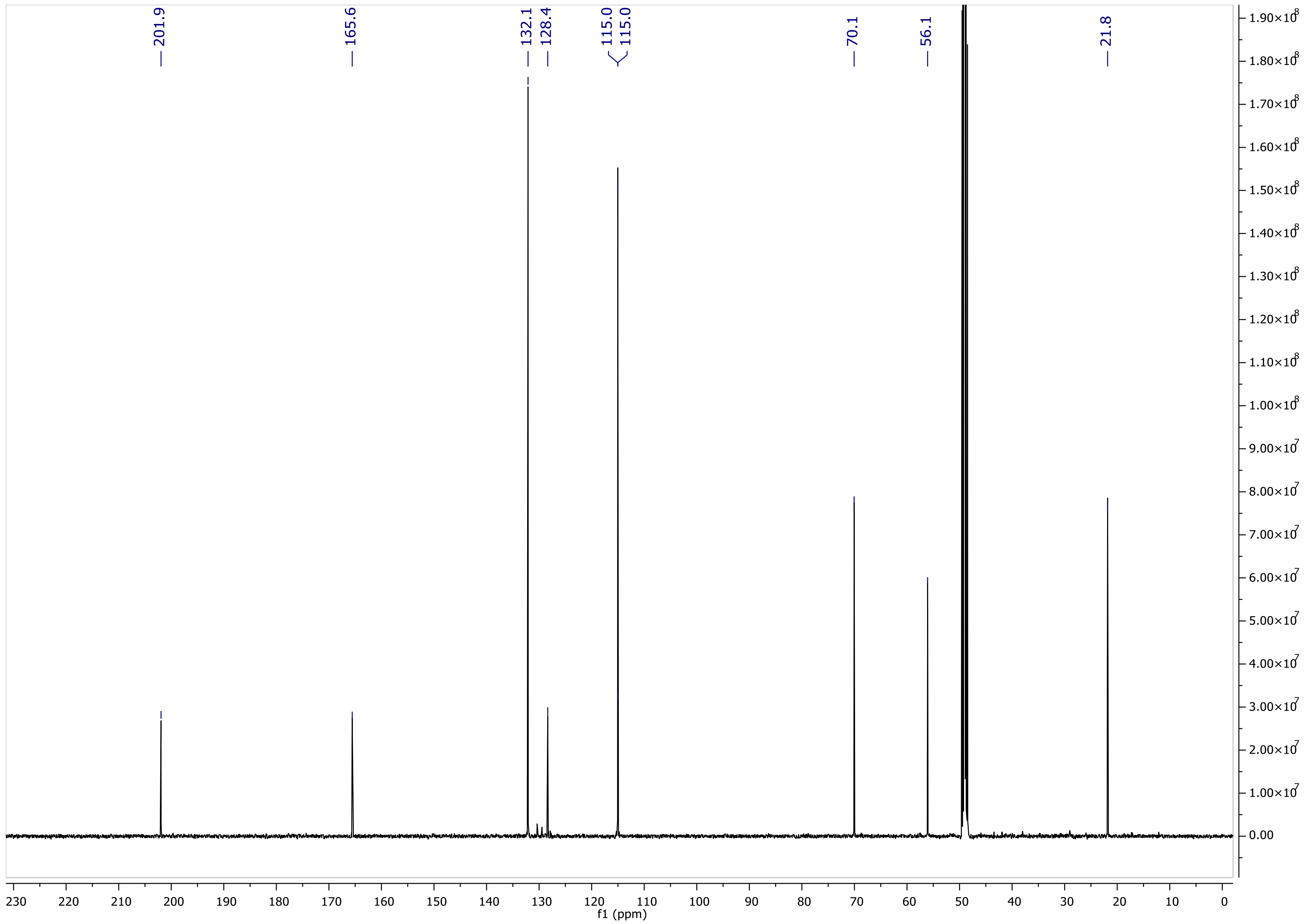

Figure 91. ^{13}C NMR spectrum of **12** in methanol-d_4 at 125 MHz.

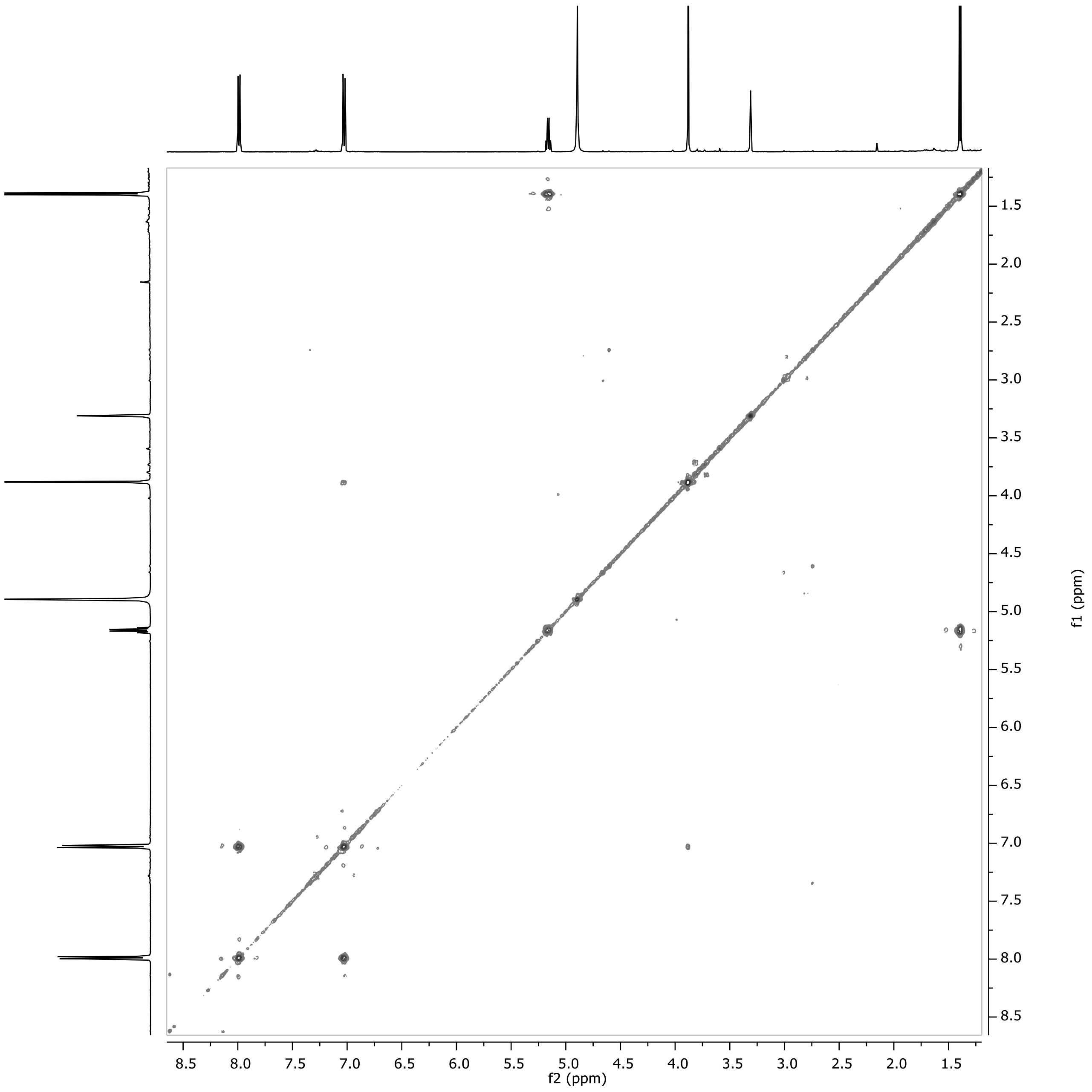

Figure S92. ^{1}H-^{1}H COSY spectrum of **12** in methanol-d_4 at 500 MHz.

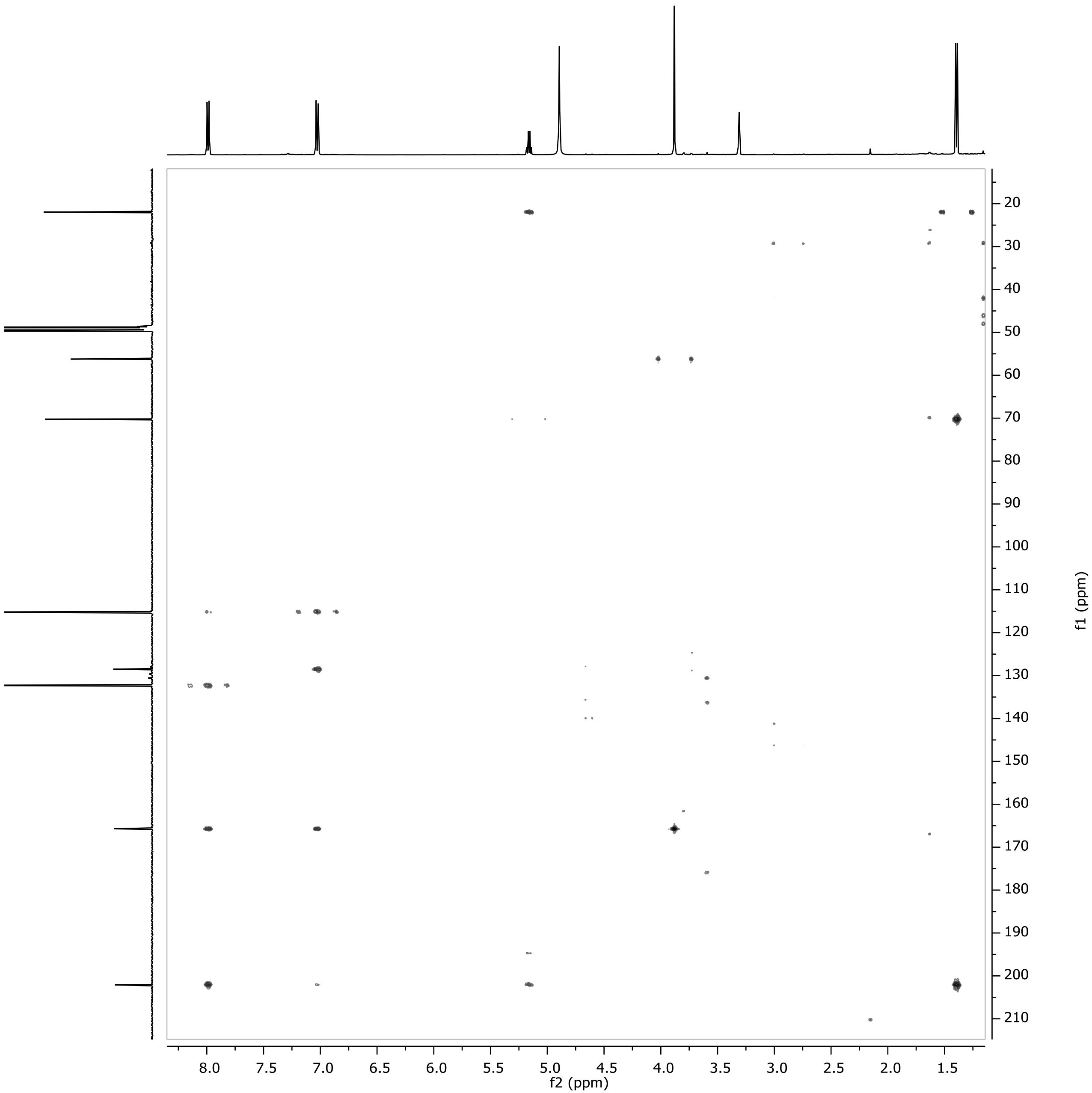

Figure S93. HMBC spectrum of **12** in methanol-d_4 at 500 MHz.

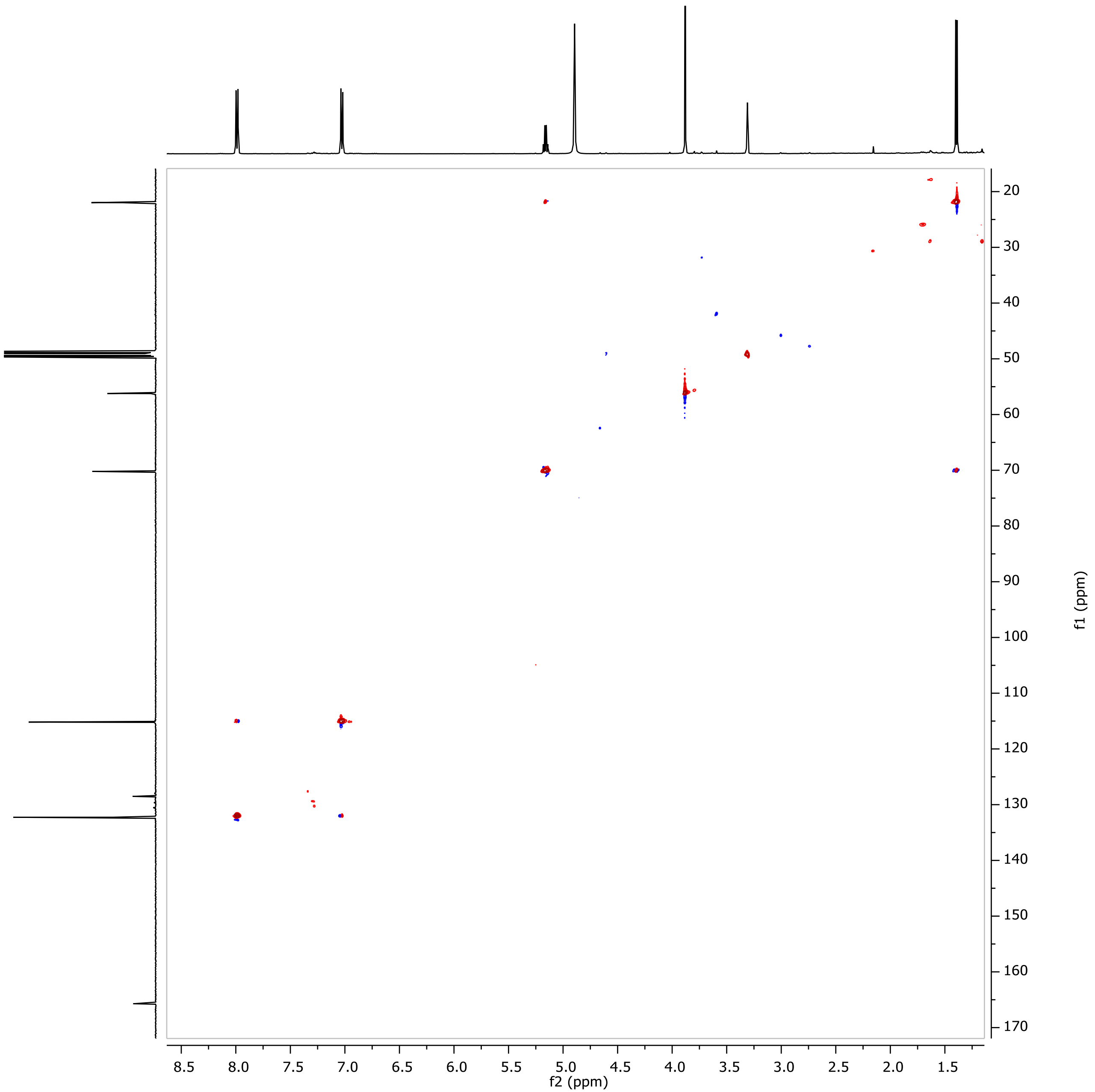

Figure S94. HSQC spectrum of **12** in methanol-d_4 at 500 MHz.

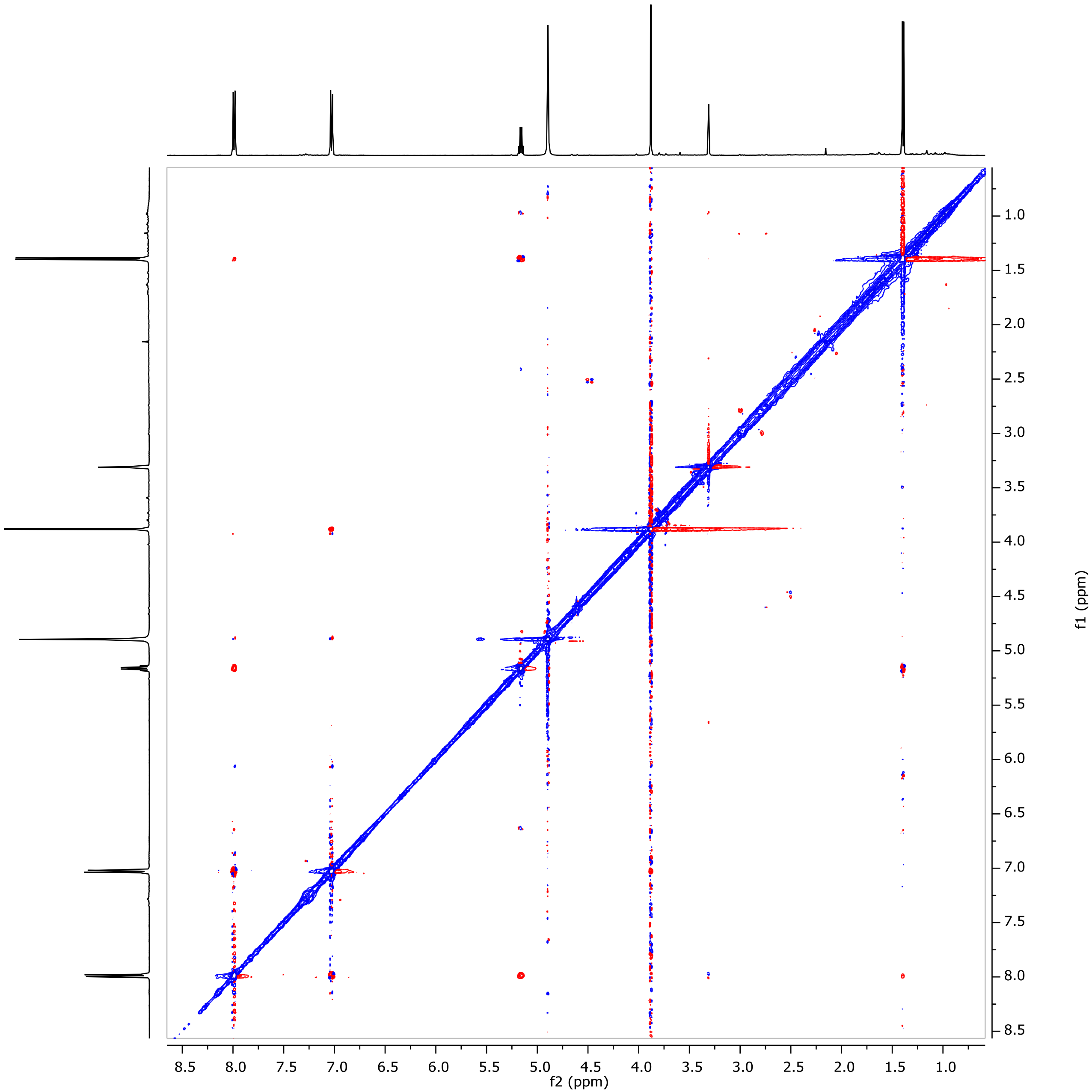

Figure 95. ROESY spectrum of **12** in methanol-d_4 at 500 MHz.
